# Human papillomavirus (HPV) type 16 and type 18 antibody concentrations after a single dose of bivalent HPV vaccine in girls aged 9–14 years compared with three doses of quadrivalent HPV vaccine in women aged 18–25 years in Costa Rica (PRIMAVERA): a non-randomised, open-label, immunobridging, non-inferiority trial

**DOI:** 10.1016/S1473-3099(25)00284-1

**Published:** 2025-12

**Authors:** Bernal Cortés, Rebeca Ocampo, Carolina Porras, Danping Liu, Mitchell H Gail, Monica S Sierra, Rolando Herrero, Douglas R Lowy, Loretto J Carvajal, Troy J Kemp, Romain Fantin, John Schussler, Allan Hildesheim, Joshua N Sampson, Ligia A Pinto, John T Schiller, Aimée R Kreimer

**Affiliations:** a Agencia Costarricense de Investigaciones Biomédicas (ACIB-FUNIN), San José, Costa Rica; b Division of Cancer Epidemiology and Genetics, National Cancer Institute, Rockville, MD, USA; c Center for Cancer Research, National Cancer Institute, Bethesda, MD, USA; d Frederick National Laboratory, Frederick, MD, USA; e Information Management Services, Silver Spring, MD, USA

## Abstract

**Background:**

In 2022, WHO recommended single-dose human papillomavirus (HPV) vaccination as an alternative schedule to multidose regimens. To provide evidence to support approval of a single-dose indication for the AS04-adjuvanted bivalent HPV vaccine (Cervarix, GlaxoSmithKline), we investigated whether the immune response to a single dose of the bivalent vaccine in girls aged 9–14 years was non-inferior to the immune response to three doses of the quadrivalent HPV vaccine (Gardasil-4, Merck) in women aged 18–25 years, a dose and population combination with demonstrated efficacy.

**Methods:**

This non-randomised, open-label, immunobridging trial enrolled girls aged 9–14 years and women aged 18–25 years in Guanacaste Province, Costa Rica. Healthy girls aged 9–14 years received one dose of bivalent HPV vaccine, whereas healthy women aged 18–25 years received three doses of quadrivalent HPV vaccine at 0, 2, and 6 months. The primary endpoint was geometric mean concentrations (GMCs) of HPV-specific serum antibodies measured by a validated virus-like-particle-based ELISA assay at 36 months. The per-protocol cohort included participants who received the correct number of doses within the predefined vaccination windows, had blood collected at the 36-month study visit for the final analysis, were seronegative at baseline for the specified HPV type, and did not receive additional HPV vaccine doses outside the study. Non-inferiority was declared when the lower bound of the 96% CI for the GMC ratio was greater than or equal to 0·67 for HPV-16 and HPV-18. Seropositivity was a secondary objective. Safety was analysed in the total vaccinated population. This trial is registered with ClinicalTrials.gov, NCT03728881, and is complete.

**Findings:**

Between April 1 and Aug 16, 2019, 620 girls and 620 women were enrolled and received their first HPV vaccination. After exclusions, 539 girls and 366 women were HPV-16 seronegative at enrolment and were included in the HPV-16 per-protocol cohort; 523 girls and 373 women were HPV-18 seronegative at enrolment and were included in the HPV-18 per-protocol cohort. At 36 months, the HPV-16 GMC was 21·4 international units (IU)/mL (95% CI 19·7–23·3) in girls in the single-dose bivalent vaccine group and 42·9 IU/mL (95% CI 38·9–47·3) in women in the three-dose quadrivalent vaccine group, resulting in a GMC ratio of 0·50 (96% CI 0·44–0·57); the HPV-18 GMC was 8·0 IU/mL (95% CI 7·4–8·8) in girls in the single-dose bivalent vaccine group and 7·2 IU/mL (95% CI 6·4–8·1) in women in the three-dose quadrivalent vaccine group, resulting in a GMC ratio of 1·11 (96% CI 0·95–1·29). At 36 months, 538 (99·8%, 95% CI 99·1–100) of 539 girls in the single-dose bivalent vaccine group were seropositive for HPV-16 compared with 366 (100%, 99·2–100) of 366 women in the three-dose quadrivalent vaccine group (p=1·00). The proportion of participants who were seropositive for HPV-18 was higher in the single-dose bivalent vaccine group (517 [98·9%, 95% CI 97·6–99·5] of 523 girls) than in the three-dose quadrivalent vaccine group (358 [96·0%, 93·6–97·6] of 373 women; p=0·0065). Two serious adverse events were reported in 620 girls and 13 serious adverse events were reported in 620 women; all serious adverse events were deemed to be unrelated to HPV vaccination.

**Interpretation:**

Non-inferior antibody responses for the single-dose bivalent HPV vaccine were seen for HPV-18 but not HPV-16, which would be insufficient evidence to motivate regulatory change, even though seropositivity approached 100% in the follow-up phase and the observed antibody concentrations were similar to protective levels seen in previous trials. Trials that directly evaluate protection afforded by single-dose HPV vaccination against persistent HPV infection will definitively address the level of protection afforded by single-dose HPV vaccination.

**Funding:**

National Cancer Institute, Cancer Research UK, and the Gates Foundation.

**Translation:**

For the Spanish translation of the abstract see Supplementary Materials section.

## Introduction

Cervical cancer could be largely prevented if girls received prophylactic human papillomavirus (HPV) vaccination and women received quality cervical cancer screening based on HPV detection, with treatment of women identified as having cervical precancer. Even though HPV vaccines were first licensed nearly 20 years ago, currently, only 21% of age-eligible girls have received at least one dose of the HPV vaccines; this proportion is even lower in countries that bear the greatest burden of cervical cancer and where high-quality screening is rarely available.^1^ Thus, the world is not close to meeting the targets set in WHO's global strategy to accelerate the elimination of cervical cancer as a public health problem.^2^ Substantial, evidence-based programmatic changes are needed to achieve the targets of vaccinating 90% of age-eligible girls worldwide, screening 70% of eligible women, and treating 90% of women with cervical precancer and cancer.^2^

HPV vaccines were initially licensed as three-dose regimens predicated on the dogma that subunit vaccines require a prime–prime–boost approach. Immunobridging trials led to regulatory changes in the schedules from three doses to two doses for adolescents,^3^ but not adults. In 2011, non-randomised data from the Costa Rica HPV Vaccine Trial (CVT) provided proof of concept that a single dose of an AS04-adjuvanted bivalent vaccine (Cervarix, GlaxoSmithKline [GSK]) administered to women aged 18–25 years might confer adequate protection against incident HPV-16 and HPV-18 infections that persist for 12 months or longer.^4^ Data from the non-randomised extension of CVT^5^, ^6^ and the India HPV vaccine trial,^7^ as well as direct evidence from randomised controlled trials of one-dose efficacy in Kenya^8^ and one-dose immunogenicity in Tanzania,^9^ motivated an update to the WHO HPV vaccine recommendations in 2022, allowing for either a one-dose or two-dose recommendation for girls and young women up to age 20 years.^10^ Although at least 50 countries have moved to a single-dose schedule for girls, and some include HPV vaccination for both sexes,^11^ some country authorities have suggested that more data are needed to modify country-level vaccine policy.

**Research in context**

**Evidence before this study**

We searched PubMed for articles published in English between database inception and Dec 31, 2024, using the terms “single dose HPV vaccination” or “one dose HPV vaccination” and “clinical trial”. From this search we identified two randomised clinical trials of multidose human papillomavirus (HPV) vaccination in India and Costa Rica in which some trial participants received only a single dose of the multidose regimen. Results from these studies suggested that a single dose of a subunit vaccine, and more specifically the HPV vaccine, could provide adequate protection against persistent HPV infection and ultimately cervical disease. As immunobridging trials have led to regulatory changes in the HPV vaccine schedule, such as decreasing the number of doses from three to two, we hypothesised that a single HPV vaccine dose of a more immunogenic vaccine—ie, the AS04-adjuvanted bivalent HPV vaccine (Cervarix, GlaxoSmithKline [GSK])—in a population with stronger immune response (ie, 9–14-year-old girls) would induce an immune response that was non-inferior, or maybe even superior, to that for the three-dose regimen of the quadrivalent HPV vaccine (Gardasil-4, Merck) given to women aged 18–25 years, a vaccine regimen that has been shown to be nearly 100% efficacious against HPV-16 and HPV-18 persistent infection and related cervical precancer. This is the first study to attempt to use immunobridging to expedite approval of a single dose of the HPV vaccines.

**Added value of this study**

In this non-randomised, open-label clinical trial, we aimed to show the non-inferior immunogenicity of a single dose of the AS04-adjuvanted bivalent HPV vaccine in girls aged 9–14 years compared with the immunogenicity of three doses of the quadrivalent HPV vaccine, administered at 0, 2, and 6 months, in women aged 18–25 years, the population in which clinical efficacy was shown. We found that 3 years after vaccination, HPV-16 and HPV-18 seropositivity approached 100% in both trial groups. The geometric mean of the antibody concentration for girls was non-inferior for HPV-18 but inferior for HPV-16. A post-hoc analysis showed immunogenicity similar to that affording protection to women in an external clinical trial (the Costa Rica HPV Vaccine Trial), suggesting that girls who received a single dose of the bivalent HPV vaccine in our trial are presumably protected against HPV infection and subsequent disease.

**Implications of all the available evidence**

The PRIMAVERA trial along with immunogenicity bridging to an external clinical trial provides evidence that subunit vaccines with repetitive structures elicit antibody responses likely sufficient to confer protection. Because we were unable to show single-dose non-inferiority in the immune response to HPV-16, we will need to focus on trials that directly evaluate single-dose efficacy of the HPV vaccines, such as the KENSHE (NCT03675256), ESCUDDO (NCT02834637), and PRISMA (NCT05237947) trials, each of which are testing the GSK bivalent and Merck nonavalent HPV vaccines. The accumulated evidence that one dose is efficacious continues to support the current WHO recommendation, with public health potential to facilitate global HPV vaccination and achieve cervical cancer elimination goals.

On the basis of the literature published up to 2018,^12^, ^13^, ^14^, ^15^ we postulated that a single HPV vaccine dose of a more immunogenic vaccine (ie, the AS04-adjuvanted bivalent vaccine) in a population with stronger immune response (ie, 9–14-year-old girls) would induce an immune response that was non-inferior, or maybe even superior, to that for the three-dose regimen of the quadrivalent HPV vaccine (Gardasil-4, Merck), a vaccine regimen that, despite inducing lower antibody concentrations in adult women compared with adolescent girls, has been shown to be efficacious against HPV infection and cervical precancer, with 100% efficacy against clinical endpoints attributed to the vaccine types in women.^16^ If successful, we viewed this as a potentially accelerated pathway to a single-dose vaccine indication for the bivalent vaccine, in view of the precedent set by a previous HPV vaccine immunobridging trial.^3^ To investigate this hypothesis, we launched the PRIMAVERA trial.

## Methods

### Study design and population

PRIMAVERA was a non-randomised, open-label, immunobridging trial done in Guanacaste Province, Costa Rica. The study aimed to show the non-inferior immunogenicity of a single-dose of the AS04-adjuvanted bivalent HPV vaccine in girls aged 9–14 years compared with the immunogenicity of three doses of the quadrivalent HPV vaccine, administered at 0, 2, and 6 months, in women aged 18–25 years, the population in which clinical efficacy has been shown.^16^ The primary assessment was 36 months after initial vaccination, in the antibody plateau phase.^6^, ^9^, ^12^

The PRIMAVERA trial was predicated on the following geometric mean concentration (GMC) ratios from the published literature: one dose to three doses 0·11 (ie, 1/9·1);^12^ bivalent HPV vaccine to quadrivalent HPV vaccine 5·8, because of the AS04 adjuvant in the bivalent vaccine;^13^ and 9–14-year-old girls to 18–25-year old women 1·8 (see appendix 2 pp 37–41 for more information on assumptions). We assumed these would have joint (multiplicative) effects and therefore predicted that the ratio of the GMC of the single-dose bivalent vaccine in girls to the GMC of the three-dose quadrivalent vaccine in women would be at least 1·14 at 24 months, and even higher in the plateau phase at 36 months.

The trial was approved by the Institutional Review Boards of Instituto Costarricense de Investigaciones Clínicas in Costa Rica on March 25, 2019 (approval number CEC-HCB-E008-2024), and the National Cancer Institute (NCI; Bethesda, MD, USA) on March 27, 2019 (19-C-N009). An independent data and safety monitoring board approved the statistical analysis plan on Dec 10, 2018, and met regularly to review field progress and adverse events. No major protocol modifications were made after study start. See appendix 2 (pp 25–84) for the study protocol and statistical analysis plan.

Community-based outreach and enrolment occurred between April 1 and Aug 16, 2019. Girls and women in selected study regions were invited according to a convenience approach (see appendix 2 pp 48–50 for further details). Eligible individuals were generally in good health, had no contraindications for receiving the HPV vaccine, did not report previous HPV vaccination, had a negative urine-based pregnancy test result (if 12 years or older), and lived in the catchment area within selected districts of the Guanacaste Province of Costa Rica. Key exclusion criteria were conditions where vaccination may be contraindicated, allergy to vaccine components, previous HPV vaccination, unwilling to provide a blood sample or permit export of blood samples to the USA, and evidence of pregnancy (appendix 2 p 49). Enrolment visits were done at study clinics. Women signed a written informed consent form, and for all girls because they were minors, consent was signed by a legal guardian. Girls aged 12 years and older signed written assent forms. For girls younger than 12 years, a study information document was administered and their verbal agreement to participate was required. Girls who became 12 years old during follow-up signed assent forms.

2 months after the first participant was vaccinated in the trial, the Costa Rican National Immunization Program initiated their HPV vaccine programme targeting schoolgirls aged 10 years with two doses of the quadrivalent HPV vaccine. We stopped enrolling 9–10-year-old girls 30 days before the start date of the national HPV vaccination campaign, to avoid overlap with the national programme. To ensure complete ascertainment of vaccination status, we actively queried external HPV vaccination status at all study visits. Because we gave the bivalent HPV vaccine to girls in PRIMAVERA while the national programme administered the quadrivalent HPV vaccine, we incorporated HPV-6 and HPV-11 serological testing as a biomarker for external HPV vaccination; an algorithm based on risk of external HPV vaccination was used to select participants for additional testing (see appendix 2 pp 4–5 for rationale and approach). Girls who were age eligible for the national programme were encouraged to obtain the additional doses by study staff. At the end of study, all girls were offered a second HPV vaccine dose.

This trial is registered with ClinicalTrials.gov, NCT03728881.

### Vaccines

The two study vaccines have been approved and recommended for use globally for more than a decade. The bivalent HPV vaccine is produced by GSK (Cervarix) and contains 20 μg each of HPV-16 and HPV-18 L1 virus-like particle (VLP). The quadrivalent HPV vaccine is produced by Merck (Gardasil-4) and contains 40 μg of HPV-16 antigen, 20 μg of HPV-18 antigen, 20 μg of HPV-6 protein, and 40 μg of HPV-11 protein. Besides the differences in HPV genotypes included, the bivalent vaccine has a proprietary adjuvant called AS04, consisting of monophosphoryl lipid A (MPL) and aluminium hydroxide. MPL is a detoxified bacterial lipopolysaccharide which is a Toll-like receptor 4 agonist involved in activation of innate and adaptive immune responses. The quadrivalent vaccine uses amorphous aluminium hydroxyphosphate sulfate as the adjuvant.

### Procedures

Eligible participants had a prevaccination blood sample collected. Participants aged 9–14 years received one dose of the bivalent HPV vaccine while participants aged 18–25 years received the first dose of the quadrivalent HPV vaccine. Women in the three-dose quadrivalent vaccine group returned to the study clinics 2 months and 6 months later to receive the second and third doses of the quadrivalent vaccine, respectively. After each 0·5 mL intramuscular vaccine dose, participants were monitored for 15 min for reactogenicity and adverse events. Following each vaccination, participants were told to report to study staff any serious problems they had in the month after each vaccine administration. Girls in the single-dose bivalent vaccine group were contacted at 6 months to inquire about vaccine reactions and any other adverse events; women in the three-dose quadrivalent vaccine group were queried at the study visits that followed each vaccine administration. These queries constituted the active adverse events monitoring phase. Thereafter, adverse events reported spontaneously to the study were also documented. All adverse events were assessed, followed to resolution, and reported according to Costa Rican and NCI regulations.

Three annual follow-up study visits occurred at 12 months, 24 months (interim analysis), and 36 months (final analysis) after initial vaccination and were done at study clinics or participants' homes, according to their preference. At each visit, a blood sample was collected for primary endpoint ascertainment, transported in cold boxes to the biorepository for serum separation and aliquoting, freezing, and subsequent shipment to the US NCI Central Repository (Frederick, MD).

To reduce the potential for batch effects, serum samples were tested in two batches, one for the interim analysis (batch 1), including all 0-month, 12-month, and 24-month samples, and one for the final analysis (batch 2), including all 12-month, 24-month, and 36-month samples. All samples were tested in NCI's Frederick National Laboratory for Cancer Research, HPV Serology Laboratory (Frederick, MD, USA).^17^, ^18^ HPV-16 and HPV-18 serum antibody concentrations were measured by a validated VLP-based ELISA (appendix 2 pp 6–7), as described elsewhere.^17^ Prespecified seropositivity cutoffs were at least 1·41 international units (IU)/mL for HPV-16 and at least 1·05 IU/mL for HPV-18;^19^, ^20^, ^21^ results below the lower limit used half the cutoff value (0·70 IU/mL for HPV-16 and 0·52 IU/mL for HPV-18). Each HPV genotype has unique international standards and inference cannot be drawn by absolute comparisons by type. HPV serological testing was highly reproducible on the basis of duplicate testing of 5% of randomly selected samples (appendix 2 pp 9–10). HPV-16 and HPV-18 ELISA results were a valid proxy for neutralisation potential, as shown by pseudovirion-based neutralisation assay testing in 200 samples randomly selected from the 36-month serum samples (appendix 2 p 11). HPV-6 and HPV-11 antibody concentrations were assessed using the previously described ELISA method. Seropositivity cutoffs were 25·3 ELISA units (EU)/mL for HPV-6 and 8·9 EU/mL for HPV-11.

### Outcomes

The four endpoints for the primary objective were HPV-16 and HPV-18 specific antibody results obtained from blood specimens (serum) collected at the month 24 (interim) and month 36 (primary) visit (ie, four endpoints=two HPV types × two timepoints). Two additional timepoints of HPV-16 and HPV-18 antibody concentrations measured from serum collected at the 1-month visit and the 1-year visit were assessed in secondary objectives. Seropositivity, defined by dichotomised antibody concentrations, was considered a secondary outcome.

### Statistical analysis

We predicted that we would need to enrol 520 women aged 18–25 years and 520 girls aged 9–14 years (including 347 aged 11–14 years and 173 aged 9–10 years) to have 65% power to demonstrate non-inferiority at 24 months and 84% power to demonstrate non-inferiority at 36 months. The power calculation assumed that 300 women would be available for the analytical sample due to 20% not receiving all three doses, 20% lost to follow-up, and 10% seronegative at baseline, and that 264 girls would be available, resulting from 100% of 9–10-year-old girls (ie, worst case scenario) receiving vaccination outside the study, 20% lost to follow-up, and 5% of 11–14-year-old girls seropositive at baseline. The calculation also assumed that the SD of antibody concentration (on log scale) was 1·38, the common true GMC ratio for HPV-16 and HPV-18 was 1·0, and the non-inferiority threshold was 0·67. Power calculations also considered the GMC ratios of 0·8 and 1·2, with results shown in the study protocol (appendix 2 pp 61–62).

The primary analytical cohort (per-protocol cohort) included participants who received the correct number of doses within the predefined vaccination windows (applies to women in the quadrivalent vaccine group only, who had to receive the second vaccine dose 1–3 months after the initial dose, and the third vaccine dose 3 months to 1 year after the first dose; see appendix 2 p 59 for details), were ELISA seronegative at baseline for the specified HPV type, had blood collected at the 36-month study visit for the final analysis (and the 24-month visit for the interim analysis), and did not receive additional HPV vaccine doses outside the study before the 36-month blood collection for the final analysis (and the 24-month blood collection for the interim analysis) on the basis of either self-report or HPV-6 and HPV-11 serological testing. A secondary analytical cohort was restricted to girls outside the age that routine HPV vaccination was introduced (ie, aged 11–14 years at enrolment) to minimise potential for external HPV vaccination; women in this secondary cohort were defined the same as in the per-protocol cohort. Prespecified sensitivity analyses were done in additional analytical cohorts: the seropositive per-protocol cohort, which used the same definition as the per-protocol cohort except that the participants were seropositive at baseline; the serocombined per-protocol cohort, with the same definition as the per-protocol cohort except that the participants could be seropositive or seronegative at baseline; and the modified intention-to-treat cohort, which included all participants receiving the correct number of doses (as part of the trial, excluding those who received external HPV vaccine doses), those whose enrolment serology status was positive, negative, or unknown, those who received the correct number of doses but did not have the correct timing between doses, and those who had blood collected during the follow-up window.

Non-inferiority was based on the GMC ratio of the single-dose bivalent vaccine group to the three-dose quadrivalent vaccine group, and defined a priori according to the WHO Expert Committee on Biological Standardization^22^ as the lower bound of the CI for the GMC ratio greater than or equal to 0·67 for HPV-16 and HPV-18. Non-inferiority was met in the interim analysis at 24 months if the lower bound of the two-sided 99% CI of the GMC ratio was greater than or equal to 0·67, and in the final analysis at 36 months if the lower bound of the two-sided 96% CI of the GMC ratio was greater than or equal to 0·67, for both HPV-16 and HPV-18 antibodies. Therefore, the overall type 1 error rate for declaring non-inferiority for both HPV-16 and HPV-18 at either timepoint was controlled at 0·025. While the non-inferiority of the one-dose regimen was defined as both the HPV-16 and HPV-18 endpoints meeting the non-inferiority criterion, we also assessed whether the criterion was met for each endpoint separately.

The CI of the GMC ratio was calculated using generalised estimating equations with robust SEs that accounted for clustering of the participants from the same household. The regression model used the individual antibody concentration (log-transformed) as the outcome and trial group as the predictor, with an identity link function and working independence structure to account for clustering by household. p values for assessing non-inferiority were calculated using the Wald test. At 36-month final analysis, p values <0·02 were considered statistically significant, and at 24-month interim analysis, p values <0·005 were considered statistically significant.

To investigate the secondary objectives of the trial, we plotted the cumulative distribution functions of HPV-16 and HPV-18 antibody concentration by group, computed HPV-16 and HPV-18 seropositivity in the two trial groups with exact binomial CIs,^19^ and compared the proportions by group using p values from Fisher's exact test, with p values <0·05 considered statistically significant. These analyses were repeated with restriction to girls aged 11–14 years at enrolment, to account for potential external HPV vaccination as part of the national vaccination programme and then compared with findings for girls aged 9–10 years. We investigated whether baseline variables (ie, age, enrolment date, and district) affected trial results. As an exploratory analysis to visualise the antibody trajectories, we plotted the GMC of HPV-16 and HPV-18 antibodies at 12, 24, and 36-months after initial HPV vaccination in the two trial groups, stratified by HPV serostatus at the time of first HPV vaccination. Analyses were done with SAS (version 9.4).

Serious adverse events and relatedness to HPV vaccination were reported by trial group in all girls and women enrolled and vaccinated in PRIMAVERA; safety data were actively collected 6 months after each HPV vaccine dose (referred to as the active monitoring phase).

To inform results obtained in PRIMAVERA, in a post-hoc analysis, we compared HPV-16 and HPV-18 antibody concentrations in girls in the single-dose bivalent vaccine group in the PRIMAVERA trial with those in non-randomised recipients of the single-dose bivalent HPV vaccine in CVT, the trial that reported the first evidence that a single dose of the AS04-adjuvanted bivalent HPV vaccine administered to women aged 18–25 years might confer adequate protection.^4^ On the basis of available data that had used the same serological assay tested in the same NCI laboratory, we compared antibody GMCs 24 months after initial HPV vaccination in recipients of single-dose HPV vaccine from PRIMAVERA and CVT, who were HPV-16 seronegative (for the HPV-16 analysis) or HPV-18 seronegative (for the HPV-18 analysis) at the time of initial HPV vaccination.

We did an additional post-hoc investigation comparing the results of the PRIMAVERA trial with the published literature. We abstracted published data and compared the GMCs of HPV-16, HPV-18, and their ratio (HPV-16 to HPV-18) observed in DoRIS,^9^ KENSHE,^8^ and PRIMAVERA, under different combinations of age, dose, and vaccine type (note, ratios of HPV-16 to HPV-18 GMCs can be informative because the same standardised assays were performed by the same laboratory for the three studies).

### Role of the funding source

The NCI and Costa Rica Investigators were responsible for all aspects of the trial, including study design and conduct, data collection, study management, data analysis, data interpretation, and writing of the report.

## Results

**Figure 1:**
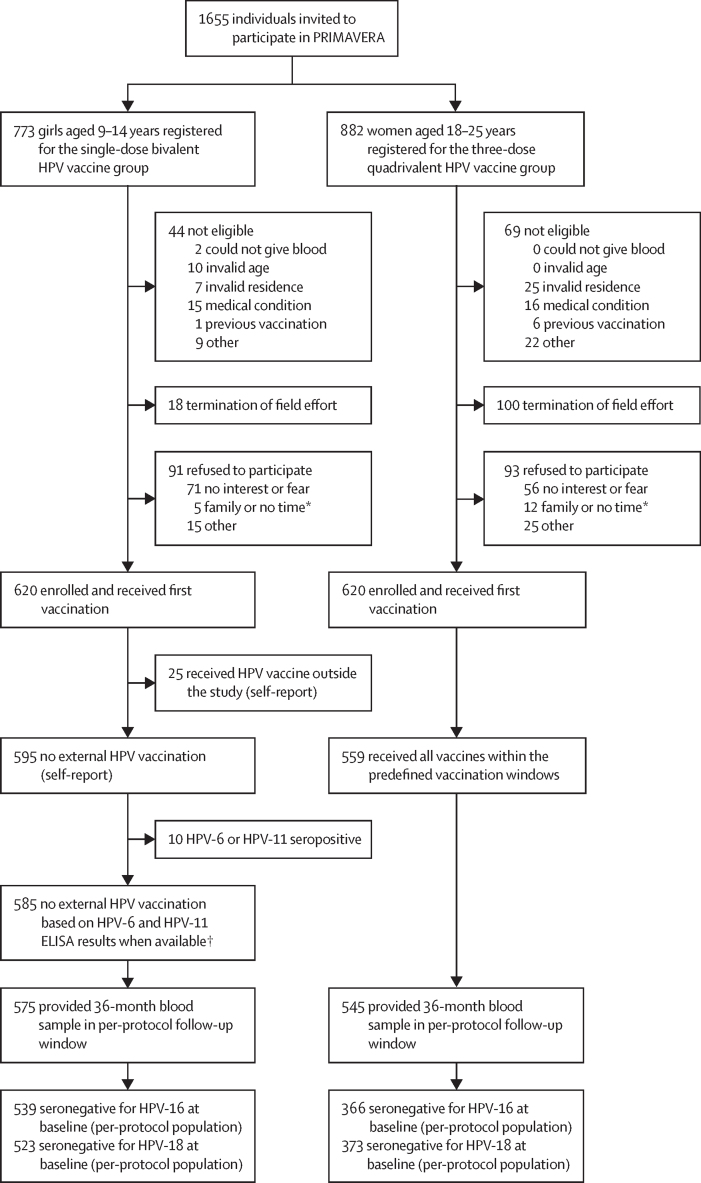
Trial profile HPV=human papillomavirus. *Family or no time indicates that the family member or potential participant refused participation because they did not have sufficient time or motivation to participate. †ELISA HPV-6 and HPV-11 results available for 116 girls aged 9–10 years at enrolment (age-eligible group for the Costa Rica national HPV vaccine programme) and 22 girls aged 11–14 years at enrolment (13 whose HPV-16 or HPV-18 titre ratio increased by at least four times between study visits, comparing the titre at month 12, 24, or 36 to that at month 1, and nine who did not have titre results at month 1). For details on the selection algorithm and methods see appendix 2 (pp 4–6).

Between April 1 and Aug 16, 2019, 773 girls aged 9–14 years and 882 women aged 18–25 years were invited to participate in the trial, of whom 620 girls and 620 women were enrolled and received their first HPV vaccination (figure 1); 559 women received the full three-dose quadrivalent HPV vaccine regimen, with the 6-month vaccination visits completed on March 5, 2020. Three annual follow-up study visits occurred 12, 24, and 36 months after the initial vaccination, and the last follow-up visit was completed on Oct 4, 2022. After cohort exclusions (figure 1), 539 girls and 366 women were HPV-16 seronegative at enrolment and were included in the HPV-16 per-protocol cohort, and 523 girls and 373 women were HPV-18 seronegative at enrolment and were included in the HPV-18 per-protocol cohort. In the HPV-16 per-protocol cohort, median age at enrolment was 12·0 years (SD 1·5) for girls and 21·0 years (2·2) for women, and similar proportions of participants were enrolled in calendar time and from coastal versus mountainous regions; the descriptive characteristics of the HPV-18 per-protocol cohort were similar (table 1).

**Table 1:** Descriptive characteristics of participants in the HPV-16 and HPV-18 per-protocol cohorts

		**Girls (single-dose bivalent HPV vaccine)**	**Women (three-dose quadrivalent HPV vaccine)**
**HPV-16 per-protocol cohort**			
Number		539	366
Enrolment age (years)		12·0 (1·5)	21·0 (2·2)
Enrolment month			
	April–May	195 (36%)	145 (40%)
	June–August	344 (64%)	221 (60%)
Enrolment region			
	Coastal	250 (46%)	169 (46%)
	Mountainous	289 (54%)	197 (54%)
**HPV-18 per-protocol cohort**			
Number		523	373
Enrolment age (years)		12·0 (1·5)	21·1 (2·3)
Enrolment month			
	April–May	184 (35%)	154 (41%)
	June–August	339 (65%)	219 (59%)
Enrolment region			
	Coastal	244 (47%)	163 (44%)
	Mountainous	279 (53%)	210 (56%)

Data are mean (SD) or n (%), unless otherwise stated. HPV=human papillomavirus.

In the 24-month formal interim analysis in which the α level of rejecting the null hypotheses for both HPV-16 and HPV-18 was controlled at 0·5%, non-inferiority in antibody responses was seen for HPV-18 but not HPV-16, motivating the final analysis at 36 months (table 2).

**Table 2:** HPV-16 and HPV-18 ELISA results at 36 months (final analysis) and 24 months (interim analysis) in the per-protocol cohort

		**Number seropositive/number in cohort**	**Seropositive, % (95% CI)**	p value*	**GMC, IU/mL (95% CI)**	**GMC ratio (96% CI or 99% CI)**†	**p value**‡
**36-month final analysis**							
HPV-16							
	Girls (single-dose bivalent vaccine)	538/539	99·8% (99·1–100)	1·00	21·4 (19·7–23·3)	0·50 (0·44–0·57)	1·00
	Women (three-dose quadrivalent vaccine)	366/366	100% (99·2–100)	..	42·9 (38·9–47·3)	..	..
HPV-18							
	Girls (single-dose bivalent vaccine)	517/523	98·9% (97·6–99·5)	0·0065	8·0 (7·4–8·8)	1·11 (0·95–1·29)	<0·0001
	Women (three-dose quadrivalent vaccine)	358/373	96·0% (93·6–97·6)	..	7·2 (6·4–8·1)	..	..
**24-month interim analysis**§							
HPV-16							
	Girls (single-dose bivalent vaccine)	546/547	99·8% (99·1–100)	1·00	20·6 (19·0–22·4)	0·42 (0·36–0·50)	1·00
	Women (three-dose quadrivalent vaccine)	371/371	100% (99·2–100)	..	48·8 (44·4–53·7)	..	..
HPV-18							
	Girls (single-dose bivalent vaccine)	521/530	98·3% (96·9–99·2)	0·48	7·9 (7·3–8·6)	0·90 (0·75–1·08)	<0·0001
	Women (three-dose quadrivalent vaccine)	367/376	97·6% (95·7–98·8)	..	8·8 (7·9–9·9)	..	..

GMC=geometric mean concentration. HPV=human papillomavirus. IU=international unit.

* p value (Fisher's exact test) for comparison of seropositivity between groups; p values <0·05 were considered statistically significant.

† The variance was adjusted to account for the correlation in antibody responses within families (full sisters) because there were clusters. We used generalised estimating equations with robust SEs based on families as the independent units. A 96% CI for the GMC ratio is used for the 36-month analysis to allow one-sided α spending of 0·02 each for HPV-16 and HPV-18. Likewise, for the 24-month analysis, we used 99% CIs to allow one-sided α spending of 0·005 each for HPV-16 and HPV-18. Thus, the total α was controlled at 0·05. We would reject the null hypothesis at 36 months if the lower bound of the 96% CI was greater than or equal to 0·67 for both HPV-16 and HPV-18. We would reject the null hypothesis at 24 months if the lower bound of the 99% CI was greater than or equal to 0·67 for both HPV-16 and HPV-18.

‡ One-sided p value (Wald test) for non-inferiority of the GMC ratio. At 36-month final analysis, p values <0·02 were considered statistically significant, and at 24-month interim analysis, p values <0·005 were considered statistically significant.

§ 24-month samples were retested along with 36-month samples to reduce batch effects often associated with HPV serological analyses. In the final presentation of 24-month data, the result generated in the final round of serological testing at 36 months was used.

In the HPV-16 final analysis 36 months after initial HPV vaccination, the HPV-16 GMC was significantly lower in girls in the single-dose bivalent vaccine group (21·4 IU/mL, 95% CI 19·7–23·3) than in women in the three-dose quadrivalent vaccine group (42·9 IU/mL, 95% CI 38·9–47·3), resulting in a GMC ratio of 0·50 (96% CI 0·44–0·57), which did not meet our criteria to demonstrate non-inferiority (table 2). The proportion of participants who were seropositive for HPV-16 at 36 months did not differ significantly between the single-dose bivalent vaccine group (538 [99·8%, 95% CI 99·1–100] of 539 girls) and the three-dose quadrivalent vaccine group (366 [100%, 99·2–100] of 366 women; Fisher's exact test p=1·00; table 2).

In the HPV-18 final analysis 36 months after initial HPV vaccination, the HPV-18 GMC was 8·0 IU/mL (95% CI 7·4–8·8) in girls in the single-dose bivalent vaccine group, which did not differ significantly from that for women in the three-dose quadrivalent vaccine group (7·2 IU/mL, 95% CI 6·4–8·1), resulting in a GMC ratio of 1·11 (96% CI 0·95–1·29), which met our non-inferiority criteria by the lower bound of the CI being greater than or equal to 0·67 (table 2). The proportion of participants who were seropositive for HPV-18 at 36 months was significantly higher in the single-dose bivalent vaccine group (517 [98·9%, 95% CI 97·6–99·5] of 523 girls) than in the three-dose quadrivalent vaccine group (358 [96·0%, 93·6–97·6] of 373 women; Fisher's exact test p=0·0065; table 2).

**Figure 2:**
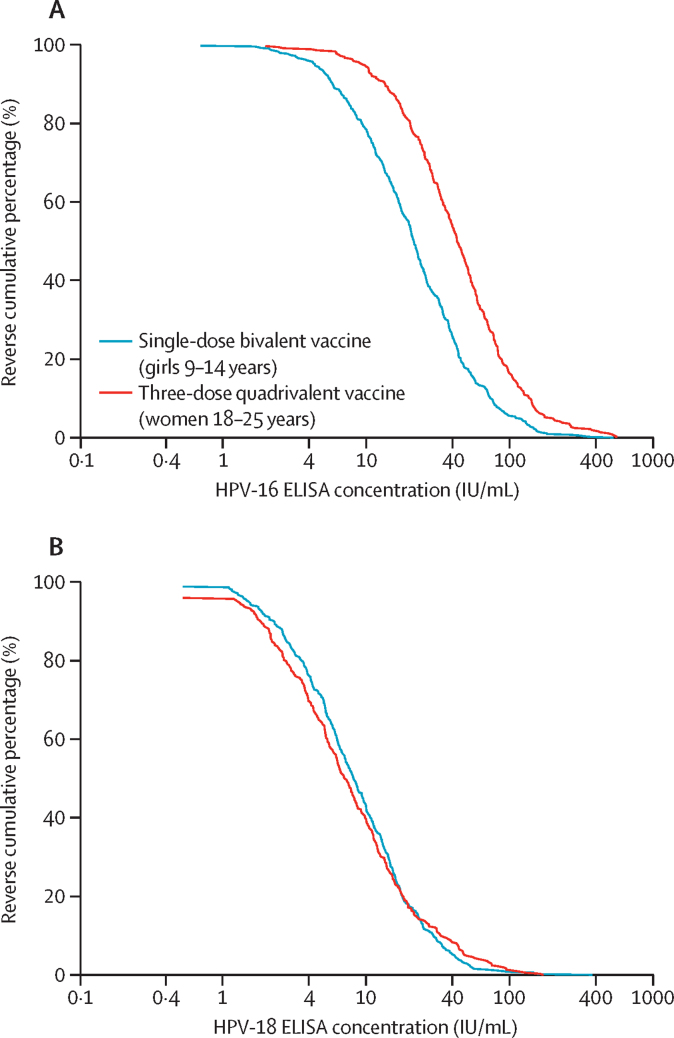
Reverse cumulative distribution plots of HPV-16 and HPV-18 concentrations One minus the cumulative distribution curves of HPV-16 (A) and HPV-18 (B) log antibody concentration at month 36 for girls aged 9–14 years who received the single-dose bivalent vaccine and women aged 18–25 years who received three doses of the quadrivalent vaccine group (per-protocol cohort). IU=international unit.

For HPV-16, 3 years after HPV vaccination, the distribution of the immune response in women in the three-dose quadrivalent vaccine group showed consistently higher antibody concentrations compared with those in girls in the single-dose bivalent vaccine group (figure 2A). For HPV-18, 3 years after initial HPV vaccination, the cumulative distribution curves nearly overlapped for girls in the single-dose bivalent vaccine group and women in the three-dose quadrivalent vaccine group (figure 2B). Similar results were seen in the interim analysis 24 months after initial HPV vaccination (appendix 2 p 12).

The post-hoc comparison of HPV-16 and HPV-18 antibody responses between girls in the PRIMAVERA trial versus women in the CVT trial^4^ (in which one dose of bivalent HPV vaccine was highly efficacious against incident HPV-16 and HPV-18 infection that persisted 12 months or longer over 4 years of follow-up), 24 months after initial HPV vaccination (given the availability of data), showed that antibody responses did not differ significantly between CVT participants and PRIMAVERA participants who received one dose of the bivalent vaccine (GMC ratio for HPV-16 of 1·24 [95% CI 0·96–1·61] and for HPV-18 of 1·09 [0·87–1·38]; table 3).

**Table 3:** Post-hoc comparison of PRIMAVERA versus CVT results

	**Number seropositive/number in cohort**	**Seropositive at 24 months, % (95% CI)**	**GMC, IU/mL (95% CI)**	**GMC ratio (95% CI)**
**HPV-16**				
Girls (PRIMAVERA)	540/541	99·8% (99·1–100)	20·6 (19·0–22·4)	1·24 (0·96–1·61)
Women (CVT)	85/86	98·8% (94·4–99·9)	16·6 (13·0–21·3)	..
**HPV-18**				
Girls (PRIMAVERA)	515/524	98·3% (96·9–99·2)	7·9 (7·3–8·6)	1·09 (0·87–1·38)
Women (CVT)	81/82	98·8% (94·1–99·9)	7·2 (5·8–9·0)	..

Post-hoc comparison of ELISA results at 24 months for girls who received a single dose of the bivalent HPV vaccine in the PRIMAVERA trial (per-protocol population) versus an efficacy population of women aged 18–25 years who received a single dose of the bivalent HPV vaccine in CVT.^4^ CVT=Costa Rica HPV Vaccine Trial. GMC=geometric mean concentration. HPV=human papillomavirus. IU=international unit.

HPV-16 and HPV-18 antibody concentrations 12, 24, and 36 months after initial HPV vaccination in the two trial groups, stratified by HPV serostatus at time of first HPV vaccination, are given in appendix 2 (pp 14–15).

In the secondary cohort restricted to girls outside the age that routine HPV vaccination was introduced (ie, aged 11–14 years at enrolment), girls in the single-dose bivalent vaccine group had a GMC roughly half that of women in the three-dose quadrivalent vaccine group for HPV-16, and a similar GMC for HPV-18 at 36 months of follow-up (appendix 2 p 17). Girls aged 9–10 years at vaccination had significantly higher HPV-16 and HPV-18 GMCs compared with girls aged 11–14 years at vaccination, for both HPV-16 and HPV-18 measured 1 month and 12 months after the first dose (appendix 2 p 18). Results were robust by age in finer categories (appendix 2 p 19), enrolment month (appendix 2 p 20), or enrolment region (appendix 2 p 21). Sensitivity analyses that evaluated trial outcomes in modified analytical cohorts (ie, the serocombined per-protocol cohort and modified intention-to-treat cohort) were consistent with the primary analysis (appendix 2 pp 22–23).

In the published data,^8^, ^9^ with restriction to GMCs measured using ELISA assay on 24-month samples (to ensure comparability), we found that the GMC of HPV-16 is consistently about 2·5 times higher than that for HPV-18, for both vaccine products and across age bands. The DoRIS trial^9^ showed that the HPV-16 to HPV-18 ratio increased to approximately three to four in two-dose and three-dose recipients for both vaccines, suggesting that the additional doses boost HPV-16 antibodies more effectively than HPV-18 antibodies. For recipients of the three-dose quadrivalent vaccine, the HPV-16 to HPV-18 ratio observed in participants aged 18–25 years in PRIMAVERA was 5·5, which is higher than the ratio of 3·7 seen in participants aged 9–14 years in the DoRIS trial (appendix 2 p 24).

Serious adverse events were monitored for these licensed vaccines: two serious adverse events were reported in 620 girls and 13 serious adverse events were reported in 620 women; all serious adverse events were deemed to be not related to HPV vaccination (appendix 2 p 16).

## Discussion

We conducted the PRIMAVERA trial as a potentially accelerated pathway to a single-dose vaccine indication for the bivalent HPV vaccine, in view of the precedent set by a previous HPV vaccine immunobridging trial.^3^ On the basis of the GMCs seen in the two study groups in this trial, antibody concentrations were non-inferior for HPV-18 but not HPV-16. Our findings showed that 3 years after HPV vaccination, HPV-16 seropositivity was very high in both trial groups, whereas HPV-18 seropositivity was slightly but statistically significantly higher in the single-dose bivalent vaccine group compared with the three-dose quadrivalent vaccine group. To ensure the validity and generalisability of the PRIMAVERA results, we did a post-hoc analysis to benchmark antibody concentrations observed in the single-dose HPV vaccine group in PRIMAVERA to recipients of single-dose HPV vaccine in CVT, the study that provided the first evidence of single-dose efficacy using an endpoint of incident HPV-16 and HPV-18 that persisted for 12 months or longer.^4^ We noted that the girls in the single-dose bivalent vaccine group of PRIMAVERA had similar HPV-16 and HPV-18 antibody concentrations to women who received a single dose of bivalent HPV vaccine in CVT, giving reassurance that the concentrations observed in PRIMAVERA will presumably confer strong protection against HPV infection and subsequent diseases.

New data emerged while PRIMAVERA was ongoing that suggested the antibody ratios underpinning our initial hypothesis might not be accurate. Using a single validated serological assay in the DoRIS trial, the observed one dose to three dose ratio was 0·056 instead of 0·11 from our hypothesis,^9^ the DoRIS girls to CVT women ratio (based on immunobridging to CVT) was only 1·3 (instead of 1·8),^20^ and the bivalent to quadrivalent ratio was 3·5 (instead of 5·8).^9^ Indeed, when we recomputed the HPV-16 ratio of GMCs based on these new values (0·056 × 1·3 × 3·5), we obtained a new value of 0·25, which was substantially lower than the value of 0·42 observed in PRIMAVERA at 24 months. We did the same computation for HPV-18, in which the revised predicted HPV-18 GMC at 24 months based on new data from DoRIS and DoRIS–CVT immunobridging was 0·38 (0·093 × 1·23 × 3·3), which is obviously much lower than what was observed in the PRIMAVERA trial of 0·90. These new data emphasise the importance of drawing conclusions from a single clinical trial rather than relying on inferences based on datasets collected across multiple trials, as well as the potential caveats associated with making comparisons across assays.

Several key inferences were made from our assessment of the HPV-16 to HPV-18 ratios in the published literature (that were generated using the same assay). We now understand that relative priming of antibody responses to the HPV-16 and HPV-18 VLPs between the bivalent and quadrivalent vaccines is essentially the same, making it unlikely that our finding of non-inferiority for HPV-18, but not HPV-16, is due to superior intrinsic immunogenicity of the bivalent vaccine's HPV-18, but not HPV-16, VLPs. It is also apparent that the additional doses in the three-dose series boost HPV-16 antibodies more effectively than HPV-18 antibodies. We also observed the suggestion of an age–dose interaction, where participants aged 18–25 years had a greater relative boost in HPV-16 antibodies than participants aged 9–14 years. However, it is hard to explain why the age effect with boosting would be greater for HPV-16 than HPV-18.

One possibility is that more of the participants aged 18–25 years had previous exposure to HPV-16 than HPV-18, and therefore more often mounted a memory response to HPV-16, leading to higher mean plateau GMCs. A previous study showed that women aged 16–26 years who were seropositive and HPV DNA PCR negative who received three doses of nonavalent HPV vaccine (Gardasil-9, Merck) had substantially higher HPV-16 and HPV-18 antibody concentrations at 24 months and 36 months compared with the per-protocol study population.^21^ It seems possible that some women who tested seronegative in our study had generated memory B cells in response to previous infections that could be activated. However, the data supporting higher HPV-16 than HPV-18 infection rates in this population of young women are mixed. In CVT, where cervicovaginal HPV DNA was assessed in women aged 18–25 years from the same catchment area, the point prevalence for HPV DNA at entry was substantially higher for HPV-16 than HPV-18 (8·3% *vs* 3·2%); however, seroprevalence at entry in CVT was equal for the two types (23%).^23^ Seroprevalence in the young women in PRIMAVERA at entry was also similar for the two types. It is important to note that cutoff points for seropositivity are somewhat arbitrary depending on whether sensitivity or specificity are optimised, and the low-level antibody responses typically seen after natural infection are likely more subject to misclassification than the more robust antibody responses induced by vaccination.

We further considered why the results of the DoRIS trial^9^ failed to predict our findings and postulated that it might be related to vaccine product. Specifically, in PRIMAVERA we used a quadrivalent vaccine (containing 40 μg of HPV-16 antigen *vs* 20 μg of HPV-18 antigen, a two-fold difference), whereas a nonavalent vaccine (Gardasil-9) was used in DoRIS (containing 60 μg of HPV-16 *vs* 40 μg of HPV-18, a 1·5-fold difference). We also note that, compared with the bivalent vaccine, Gardasil-4 has a two times higher concentration of HPV-16 L1 VLP but an equal concentration of HPV-18 L1 VLP. Since we are looking at small differences in GMCs, relatively small differences in the VLP amount could contribute to the ability to boost effectively. Yet, this hypothesis is challenging to reconcile with the finding from DoRIS that the third dose of the nonvalent vaccine gave no boosting for HPV-16 (or HPV-18) while GMCs for the bivalent vaccine did increase with the additional boosting, even though the bivalent vaccine contained less antigen, leading one to hypothesise that an adjuvant effect was more essential than a dose effect. Additionally, the quadrivalent and nonavalent vaccines were previously shown to generate almost identical HPV-16 and HPV-18 antibody responses after three doses, despite the differences in VLP composition and doses. It is unlikely that our findings could be caused by assay performance, in view of the substantial quality control work done and documentation of high reproducibility and validity compared with in vitro neutralisation. In short, we extensively considered several explanations for the findings of differential antibody responses to HPV-16 and HPV-18 in relation to vaccination regimens and the age of recipients, and did not come up with a strongly defensible explanation supported by data.

Our findings highlight some of the limitations associated with immunobridging studies, especially those with complex designs that compare across vaccine products, age, and other issues such as geographical area, which complicate the set of comparisons. Antibody bridging studies have been used to generalise efficacy and justify wider use. However, there might be too much emphasis on GMCs and ratios of the GMCs. Our data from PRIMAVERA suggest that these measures might not be an appropriate equivalence to vaccine efficacy, especially where a quantitative immune correlate of protection has not been established, as in this case. Further, reviewing the distribution of the antibody response is essential, especially in cases where the tails of the distribution greatly affect the geometric mean. With our newly informed thinking, we could have prespecified different criteria for success, such as the percentage of participants who were antibody seropositive in the plateau phase, as used in the DoRIS trial,^9^ or the number or percentage of individuals observed to be above a particular threshold, despite the absence of a correlate of protection. In PRIMAVERA, nearly all trial participants were HPV-16 and HPV-18 seropositive following HPV vaccination. Furthermore, the distribution of the immune response largely overlapped between the two trial groups. For HPV-16, the mean was shifted but remained above the seropositive threshold and is likely still protective. Criteria for non-inferiority will need to be revisited as the field continues to advance towards a correlate of protection. Broad use of available international standards would also have alleviated the imperfect comparisons conducted across studies that used different assay methods, even if we use ratios to try to mitigate that bias.

Our study had strengths and limitations. The Costa Rica field team continues to conduct HPV vaccine epidemiological research and clinical trials with tremendous rigor, as evidenced by trial participation and compliance. Furthermore, the laboratory assays continue to perform exceptionally well, with high levels of reproducibility and validity. One might consider the follow-up to only 36 months a limitation, which is when we expected single-dose antibody concentrations to have essentially plateaued. However, concentrations in recipients of three vaccine doses continued to wane, although at a much slower rate than in the first year. This occurrence is illustrated in the increase (albeit non-significant) in the ratio of one-dose to three-dose antibody concentrations from 24 months to 36 months as well as in the 16-year immunological follow-up data from CVT.^24^ The difference in the kinetics of antibody waning is likely due to the induction of plasmablasts of variable life spans from the memory B cells induced by the boost, whereas the priming dose only activates naive cells which apparently die rather quickly if they do proceed to becoming long-lived plasma cells.^25^ Therefore, the ratio of the GMCs between the two PRIMAVERA study groups might have approached 1·0 for HPV-16 if enough follow-up time was permitted. Furthermore, the observed two-fold difference in antibody concentration between study groups is not likely to be meaningful, as vaccinologists typically consider orders of magnitude in differences to affect efficacy. We relied on antibody concentrations as the trial outcome; we cannot rule out that a well characterised correlate of protection might have achieved the threshold of non-inferiority. Cervical sample collection in women in the three-dose quadrivalent vaccine group would have facilitated assessment of HPV DNA status at time of vaccination and throughout follow-up, which might have informed questions around anamnestic boosting of prevaccine antibodies and during the follow-up phase; it is important to reiterate that local infection appears to have minimal impact on natural immunity^26^ and vaccine-induced immunity in the plateau phase.^21^

The PRIMAVERA trial provides additional evidence that subunit vaccines with repetitive structures elicit antibody responses that have been shown to confer protection and demonstrates the importance of conducting a clinical trial to evaluate non-inferiority of antibody responses. On the basis of results from multiple studies, we postulated that we could show immunological non-inferiority using an immunobridging approach based on differing immune responses by age, vaccine product, and number of doses. Because we were unable to show non-inferiority of the antibody response for HPV-16 by the single-dose bivalent vaccine, we will instead need to focus on trials that directly evaluate single-dose HPV vaccine efficacy, such as the KENSHE (NCT03675256),^8^ ESCUDDO (NCT02834637),^27^ and PRISMA (NCT05237947) trials, to evaluate and document protection afforded by single-dose HPV vaccines. The accumulated evidence that one dose is efficacious continues to support the current WHO recommendation, with enormous public health potential to facilitate global HPV vaccination and achieve cervical cancer elimination goals.

### Contributors

### Data sharing

We encourage collaborations with external investigators. Participant data can be shared with outside collaborators for research to understand more about HPV vaccination and the immune response to the vaccine. Outside collaborators can apply to access our protocols and data from the PRIMAVERA trial. For the trial summary, current publications, and contact information for data access see https://dceg.cancer.gov/research/cancer-types/cervix/primavera.

## Declaration of interests

RH declares honoraria for participating as faculty in a vaccinology course for Merck executives in the past 36 months. All other authors declare no competing interests.

## Supplementary Materials

Spanish translation of the abstract
