# Supplementary material for: Human papillomavirus (HPV) type 16 and type 18 antibody concentrations after a single dose of bivalent HPV vaccine in girls aged 9–14 years compared with three doses of quadrivalent HPV vaccine in women aged 18–25 years in Costa Rica (PRIMAVERA): a non-randomised, open-label, immunobridging, non-inferiority trial
Source: Lancet Infect Dis. 2025 Dec;25(12):1314–24. doi: 10.1016/S1473-3099(25)00284-1 (PMC12630073; doi:10.1016/S1473-3099(25)00284-1)
Supplement: Spanish translation of the abstract [file mmc1.pdf]

THE LANCET
Infectious Diseases

## Supplementary appendix 1

This translation in Spanish was submitted by the authors and we reproduce it as supplied. It has not been peer reviewed. *The Lancet*'s editorial processes have only been applied to the original in English, which should serve as reference for this manuscript.

Los autores nos proporcionaron esta traducción al español y la reproducimos tal como nos fue entregada. No la hemos revisado. Los procesos editoriales de *The Lancet* se han aplicado únicamente al original en inglés, que debe servir de referencia para este manuscrito.

Supplement to: Cortés B, Ocampo R, Porras C, et al. Human papillomavirus (HPV) type 16 and type 18 antibody concentrations after a single dose of bivalent HPV vaccine in girls aged 9–14 years compared with three doses of quadrivalent HPV vaccine in women aged 18–25 years in Costa Rica (PRIMAVERA): a non-randomised, open-label, immunobridging, non-inferiority trial. *Lancet Infect Dis* 2025; published online July 16. https://doi.org/10.1016/S1473-3099(25)00284-1.

Resumen

Antecedentes En el 2022, la OMS recomendó la vacunación con dosis única contra el virus del papiloma humano (VPH) como esquema alternativo a los regímenes multidosis. Para aportar evidencia que respaldara la aprobación de una indicación de dosis única para la vacuna contra el VPH bivalente adyuvada con AS04 (Cervarix, GlaxoSmithKline), investigamos si la respuesta inmune a una dosis única de la vacuna bivalente en niñas de 9 a 14 años no era inferior a la respuesta inmune a tres dosis de la vacuna tetravalente contra el VPH (Gardasil-4, Merck) en mujeres de 18 a 25 años, una combinación de dosis y población con eficacia demostrada.

Métodos En este ensayo de puente inmunológico no aleatorizado, abierto, se reclutaron niñas de 9 a 14 años y mujeres de 18 a 25 años de la provincia de Guanacaste, Costa Rica. Niñas sanas de 9 a 14 años recibieron una dosis de la vacuna bivalente contra el VPH, mientras que mujeres sanas de 18 a 25 años recibieron tres dosis de la vacuna tetravalente contra el VPH a los 0, 2 y 6 meses. El criterio de valoración principal fue la media geométrica de las concentraciones (MGC) de anticuerpos séricos específicos del VPH medidos a los 36 meses mediante un ensayo ELISA validado basado en partículas semejantes al virus. La cohorte según el protocolo para el análisis final del estudio incluyó a las participantes que recibieron el número correcto de dosis dentro de los intervalos predefinidos de vacunación, a las que se les extrajo sangre en la visita a los 36 meses, que eran seronegativas al inicio del estudio para el tipo de VPH especificado y que no recibieron dosis adicionales de la vacuna contra el VPH fuera del estudio. Se declaró la no inferioridad cuando el límite inferior del IC del 96% para la razón de la MGC era mayor o igual a 0.67 para el VPH-16 y el VPH-18. La seropositividad fue un objetivo secundario. La seguridad se analizó en la población total vacunada. Este ensayo está registrado en ClinicalTrials.gov, NCT03728881, y ya finalizó.

Hallazgos Entre el 1 de abril y el 16 de agosto de 2019, 620 niñas y 620 mujeres se incluyeron en el estudio y recibieron su primera vacunación contra el VPH. Después de las exclusiones, se incluyeron en la cohorte por protocolo de VPH-16, 539 niñas y 366 mujeres que eran seronegativas al VPH-16 al inicio del estudio; y se incluyeron en la cohorte por protocolo de VPH-18, 523 niñas y 373 mujeres que eran seronegativas al VPH-18 al inicio del estudio. A los 36 meses, la MGC del VPH-16 fue de 21.4 unidades internacionales (UI)/mL (IC del 95%: 19.7-23.3) en las niñas del grupo de dosis única de la vacuna bivalente y de 42.9 UI/mL (IC del 95%: 38.9-47.3) en las mujeres del grupo de tres dosis de la vacuna tetravalente, resultando en una razón de la MGC de 0.50 (IC del 96%: 0.44-0.57); la MGC del VPH-18 fue de 8.0 UI/mL (IC del 95%: 7.4-8.8) en las niñas del grupo de dosis única de la vacuna bivalente y de 7.2 UI/mL (IC del 95%: 6.4-8.1) en las mujeres del grupo de tres dosis de la vacuna tetravalente, resultando en una razón de MGC de 1.11 (IC del 96%: 0.95-1.29). A los

36 meses, 538 (99.8%, IC 95% 99.1-100) de las 539 niñas del grupo de dosis única de la vacuna bivalente eran seropositivas al VPH-16, en comparación con 366 (100%, 99.2-100) de las 366 mujeres del grupo de tres dosis de la vacuna tetravalente (p=1.00). La proporción de participantes seropositivas al VPH-18 fue mayor en el grupo de dosis única de la vacuna bivalente (517 [98.9%, IC 95% 97.6-99.5] de 523 niñas) que en el grupo de tres dosis de la vacuna tetravalente (358 [96.0%, 93.6-97.6] de 373 mujeres; p=0.0065). Se reportaron dos eventos adversos serios en las 620 niñas y 13 eventos adversos serios en las 620 mujeres; todos los eventos adversos serios no se estaban relacionados con la vacunación contra el VPH.

Interpretación Se observaron respuestas de anticuerpos no inferiores para la vacuna VPH bivalente en dosis única para el VPH-18 pero no para el VPH-16, lo que sería una evidencia insuficiente para motivar un cambio regulatorio, aunque la seropositividad se aproximó al 100% en la fase de seguimiento y las concentraciones de anticuerpos observadas fueron similares a los niveles de protección obtenidos en ensayos clínicos anteriores. Los ensayos que evalúan directamente la protección brindada por la vacunación de dosis única contra la infección persistente por VPH abordarán definitivamente el nivel de protección brindada por la vacunación de dosis única contra el VPH.
