# Supplementary material for: Human papillomavirus (HPV) type 16 and type 18 antibody concentrations after a single dose of bivalent HPV vaccine in girls aged 9–14 years compared with three doses of quadrivalent HPV vaccine in women aged 18–25 years in Costa Rica (PRIMAVERA): a non-randomised, open-label, immunobridging, non-inferiority trial
Source: Lancet Infect Dis. 2025 Dec;25(12):1314–24. doi: 10.1016/S1473-3099(25)00284-1 (PMC12630073; doi:10.1016/S1473-3099(25)00284-1)
Supplement: Supplementary appendix 2 [file mmc2.pdf]

THE LANCET
Infectious Diseases

Supplementary appendix 2

This appendix formed part of the original submission and has been peer reviewed. We post it as supplied by the authors.

Supplement to: Cortés B, Ocampo R, Porras C, et al. Human papillomavirus (HPV) type 16 and type 18 antibody concentrations after a single dose of bivalent HPV vaccine in girls aged 9–14 years compared with three doses of quadrivalent HPV vaccine in women aged 18–25 years in Costa Rica (PRIMAVERA): a non-randomised, open-label, immunobridging, non-inferiority trial. *Lancet Infect Dis* 2025; published online July 16. https://doi.org/10.1016/S1473-3099(25)00284-1.

PRIMAVERA Supplementary Methods and Results, Protocol, and SAP

CONTENTS

I. PRIMAVERA SUPPLEMENTAL MATERIALS

II. PRIMAVERA PROTOCOL

III. STATISTICAL ANALYSIS PLAN

Supplemental materials including supplementary methods, results, trial protocol and statistical analysis plan for the manuscript entitled: A non-randomized, open label immunobridging trial comparing HPV16/18 antibody levels in girls who received a single dose of Cervarix® vaccine compared to women who received three doses of Gardasil®: The PRIMAVERA Trial

Authors: Bernal Cortés[1*], PharmD, Rebeca Ocampo[1*], MD, Carolina Porras[1*], et al.

Supplemental methods: Rationale and Methods for HPV6/11 testing.

Rationale. In 2019, the Costa Rica National Vaccine Program provided two doses of the quadrivalent HPV vaccine (includes types HPV6, 11, 16, 18) to 10 years old girls in Costa Rica. During the PRIMAVERA trial, it is therefore likely that 10-year-old study participants were offered these doses in 2019, and that the subsequent year, girls aged nine at study enrollment aged into the program.

In consultation with the DSMB, we encouraged participation in the program. PRIMAVERA girl participants received a single dose of the bivalent HPV vaccine (includes HPV types 16/18). To determine if additional HPV vaccination was applied with the Gardasil-4® HPV vaccine in the girls who were vaccinated in our study with the bivalent HPV vaccine, we tested serum samples for HPV 6 and 11 antibodies as a biomarker of additional HPV vaccination. Women in PRIMAVERA received 3 doses of Gardasil-4® which contains antigens for 6 and 11. Thus, this approach could not be used to determine if the women received additional doses (note that women receiving additional HPV vaccine doses can only bias the study findings in favor of inferiority).

Methods. To detect external vaccination among girls who reported they did not receive additional doses, HPV 6 and 11 antibodies were tested among all girls aged 9 and 10 years at study entry, as they were eligible to receive additional doses in the National Program (N=116). Girls aged 11 and above at enrollment were deemed less likely to receive external HPV vaccine doses; being outside the age of the national program, they would have needed to actively seek out additional doses. Consequently, we tested for HPV 6 and 11 in girls aged 11 to 14 at enrollment whose HPV16 or HPV18 titer ratio between study visits increased by at least 4-fold, comparing the titer at month 12, 24 or 36 to that at month 1 (N=13) as well as girls aged 11 to 14 at enrollment who did not have titer results at month 1 (N=9). A

random sample of 20 women was tested to confirm the assay detects antibodies to HPV 6 and 11, which should be present because of Gardasil-4® vaccination.

All study visits including enrollment (pre-vaccination) and follow-up visits were tested in order to determine the putative timing of additional HPV vaccine doses and to confirm the absence of HPV6/11 antibodies at enrollment (prior to the start of the national program). Parallel timepoints were tested in the women to confirm assay sensitivity. Girls found to have HPV 6 and 11 vaccine-level antibodies were excluded from primary analysis as another form of reporting.

Results. All girls aged 9 and 10 at enrollment into PRIMAVERA were tested for HPV6/11 antibodies (n.b.: 28 out of 116 were deemed as outliers using the 4-fold ratio threshold). 27 girls were HPV6 and 11 seropositive and thus excluded from the primary analysis. Of those 27 girls, 19 had already been excluded for reporting external vaccination at one of their study visits. 22 out of 504 girls aged 11-14 showed a four-fold or greater increase in their GMC ratio comparing the titer at month 12, 24, or 36 to that at month 1. Of these, 2 were HPV6 and 11 seropositive and were thus excluded. There were also 2 girls who were positive for HPV6 but not HPV 11 and 1 girl who was positive for HPV 11 but not HPV 6 who were not excluded. Nineteen of the 20 women who were tested were HPV6 and 11 seropositive, the other was positive for HPV11 but not for HPV6.

Interpretation. Review of HPV16 and 18 antibody patterns in PRIMAVERA generated concerns that a very small number of girls who reported no external HPV vaccination (i.e.: as part of the national program) may have received additional HPV vaccine doses. To control for this potential threat to study validity, we investigated HPV vaccination external to the PRIMAVERA trial by testing for HPV6/11 antibodies, which should be present in Gardasil-4® vaccinees, the vaccine product used in the Costa Rica National Vaccination Program, but not among bivalent HPV vaccine recipients in PRIMAVERA.

Twentynine girls had antibody patterns associated with boosting, 10 of whom had not previously reported external vaccination. We additionally excluded those 10 girls from the ATP analytical study population.

Supplemental Laboratory Methods

Briefly, anti-HPV IgG antibodies are detected by an ELISA, with HPV16 (2.7 µg/mL) or 18 (2.0 µg/mL) VLP used as the coating antigen on microtiter plates (MaxiSorp, high binding; Nunc, Thermo Fisher Scientific, USA). The VLPs were produced in human 293TT cells, to reflect the native structure of the authentic virions. Plates were blocked in a blocking solution for 1.5 hours and then washed. Serum samples from participants were serially diluted in the blocking solution starting at 1:100 in two-fold increments. Serial dilutions of samples, standard and quality control specimens are performed in the microtiter plates and incubated for 1 hour at room temperature. Plates were washed, and HRP-conjugated anti-human IgG antibodies were added to the plate for one hour. Then the plates were washed, and a 3,3’,5,5’-Tetramethylbenzidine (TMB) solution was added for 25 minutes; the reaction was stopped with sulfuric acid. Absorbance was measured with a microtiter plate reader (Spectramax M5; Molecular Device, Sunnyvale, CA).

To evaluate the functional antibody response to HPV16 and 18 and correlation to ELISA responses, neutralizing antibody levels were measured on a subset of samples using the pseudovirion-based neutralization assay (SEAP-NA). The 293TT cells were seeded in 96-well plates (cell density of 3×10^4 cells/well) and incubated at 37° C and 5% CO_2 for ≥2 hours prior to the addition of controls and diluted samples. Serial dilutions of serum samples and controls were incubated with HPV16/18 VLPs. Then, the samples will be transferred to the 293TT cells and incubated at 37° C for 72 hours. Following incubation, supernatants will be clarified by centrifugation, then were transferred to 96-well plates and frozen at

−70° C until further testing. The Great EscAPe SEAP assay kit was used according to the manufacturer's protocol (BD Biosciences-Clontech Laboratories, Inc. Mountain View, CA). Neutralization titers were calculated by linear interpolation and defined as the reciprocal of the dilution that caused 50% reduction in SEAP activity compared to control wells. Neutralization titers reflect the mean value of duplicate testing for each sample. SEAP-NA titers below 21 for HPV16 and 16 for HPV18 were defined as seronegative.

At each study visit and each arm, we randomly selected 5% samples to be tested a second time by ELISA to evaluate the assay reproducibility. We computed the intraclass correlation coefficient (ICC) using the maximum likelihood method (Supplemental figure 1): in batch 1, the ICC for HPV 16 was 0.986 (95% CI 0.981 to 0.989) and the coefficient of variability (CV) was 3.7% (95%CI 3.3 to 4.2%) and the HPV 18 ICC was 0.987 (95%CI 0.982 to 0.990) and the CV was 4.5% (95%CI 3.9 to 5.1%). In batch 2, for HPV16, the ICC was 0.997 (95%CI 0.996 to 0.998) and the CV was 1.8% (95%CI 1.6 to 2.0%); for HPV18, the ICC was 0.996 (95%CI 0.995 to 0.997) and the CV was 2.7% (95%CI 2.4 to 3.0%). High reproducibility was confirmed by Bland-Altman plots (1) of the difference versus mean of the first and second ELISA measurements (Supplemental Figure 2).

To confirm the validity of the HPV16 and 18 ELISA results as a proxy for neutralization potential, pseudovirion-based neutralization assay testing was conducted on 200 samples randomly selected from the 36-month serum samples among 100 girls and 100 women who were HPV16 and 18 seronegative at study enrollment. The Deming slope for HPV16 was 0.928 (95%CI 0.867 to 0.989) and for HPV18 was 0.865 (95%CI 0.814 to 0.916).

Reference

1. Mansournia MA, Waters R, Nazemipour M, Bland M, Altman DG. Bland-Altman methods for comparing methods of measurement and response to criticisms. Global Epidemiology. 2021 Nov 1;3:100045.

Supplemental Figure 1. Reproducibility analysis for HPV16 and 18 based on 5% randomly selected duplicates (by study visit and arm) from the samples included in the final analysis (batch 2). Inter-cluster correlation (ICC), coefficient of variation (CV), and their corresponding 95% confidence intervals are calculated.

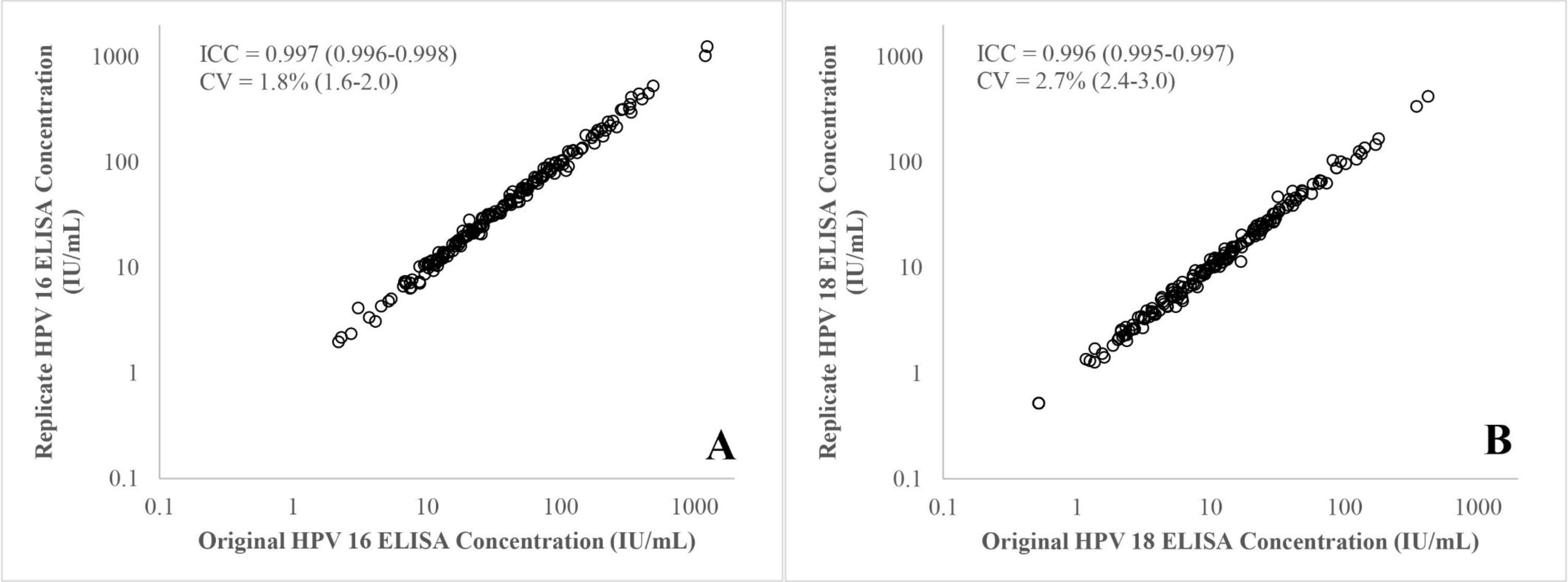

Supplemental Figure 2. Reproducibility analysis for HPV16 and 18 based on 5% randomly selected duplicates (by study visit and arm) from the samples included in the final analysis (batch 2): Bland-Altman plots of the difference versus the mean of the first and second ELISA measurements.

(A)

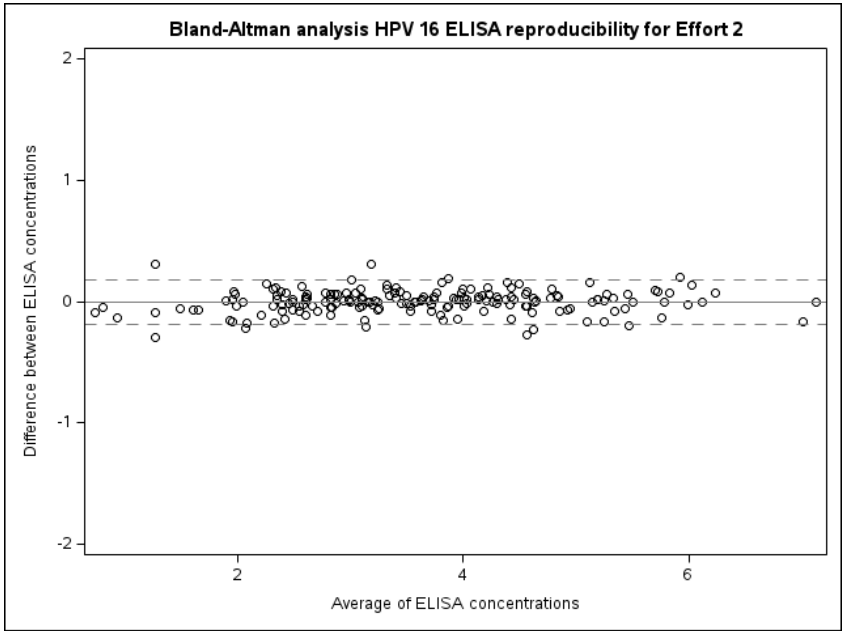

(B)

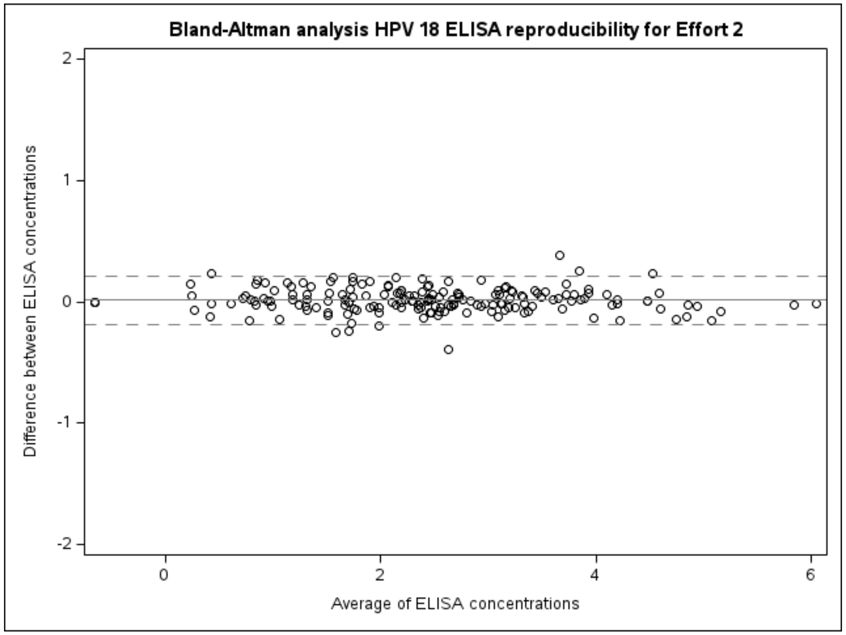

Supplemental Figure 3. Validation of the HPV16 and HPV18 ELISA assay compared to SEAP as the gold standard, evaluated on the 36-month samples among girls and women who were HPV16 and 18 antibody negative on ELISA at enrollment into the PRIMAVERA trial. Deming slopes, Pearson correlations, and their corresponding 95% CI are calculated overall and by arm.

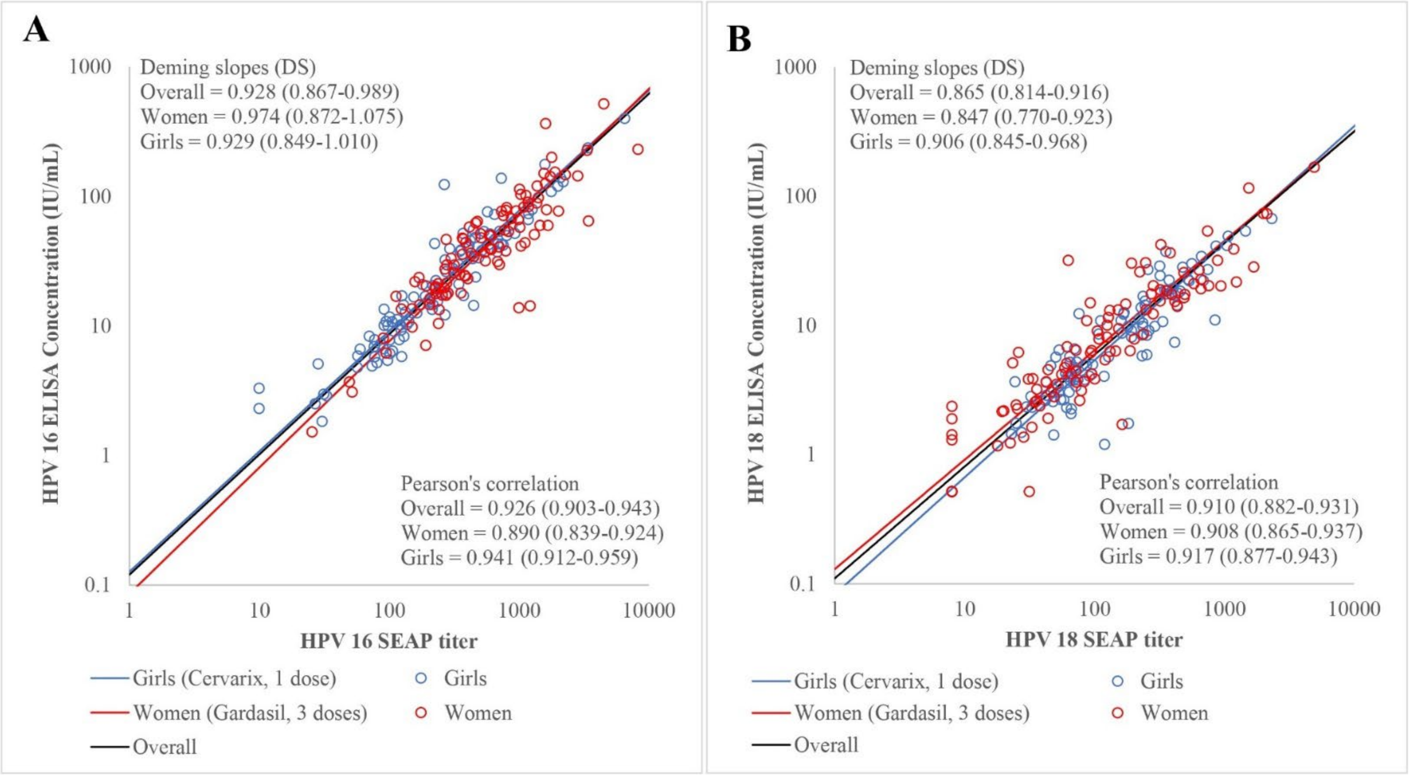

Supplemental Figure 4. One minus the cumulative distribution curves of HPV16 (panel A) and HPV18 (panel B) log antibody concentration at month 24 for girls and women in the ATP cohort.

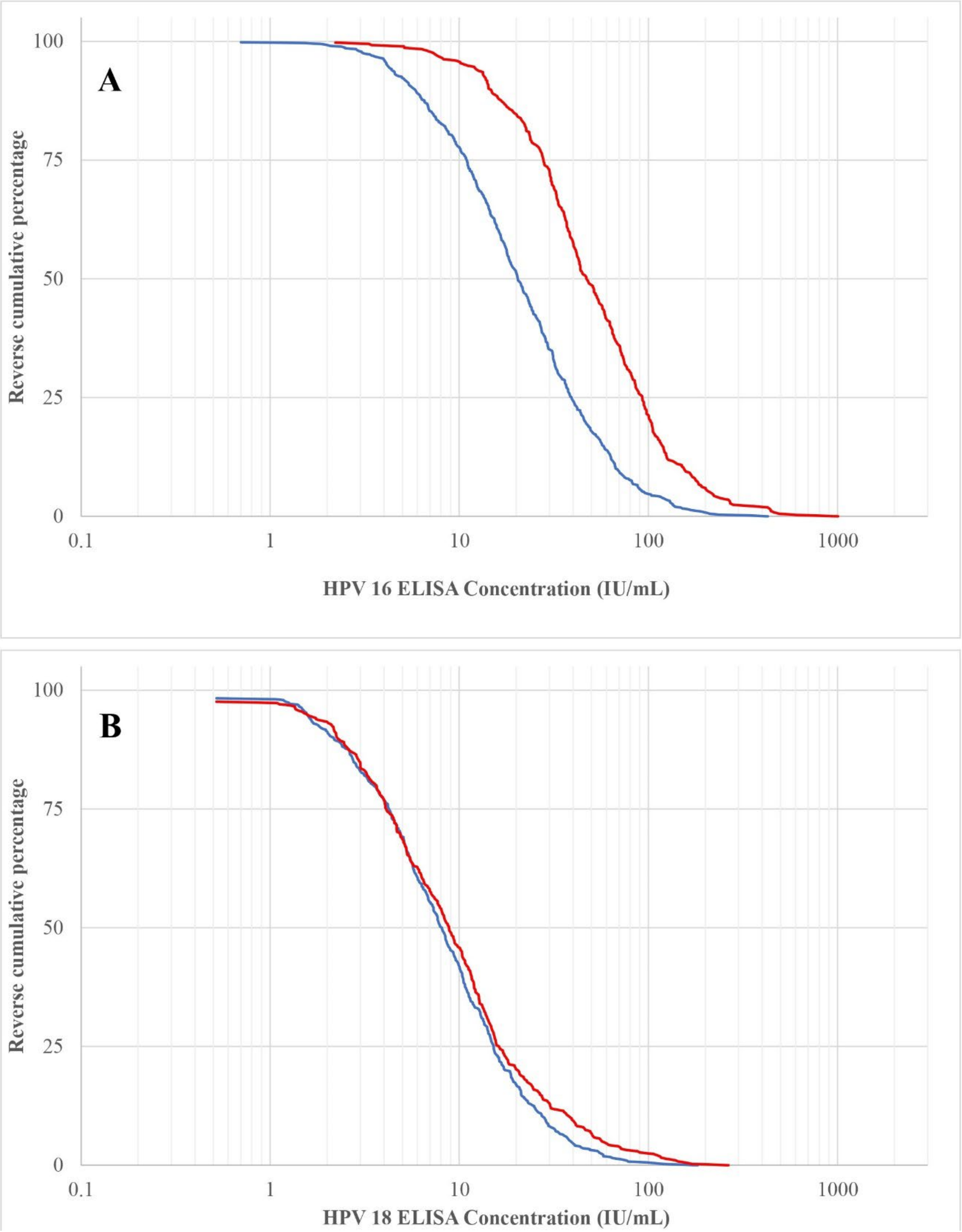

Legend. The blue line indicates girls who received a single dose of the ASO4-adjuvanted HPV vaccine (1D2V) and the red line indicates women who received three doses of the quadrivalent HPV vaccine (3D4V).

Supplementary Figure 5. HPV16 antibody levels 12, 24, and 36-months after initial HPV vaccination in the two trial arms, stratified by HPV serostatus at time of first HPV vaccination. Antibody concentration measured by ELISA.

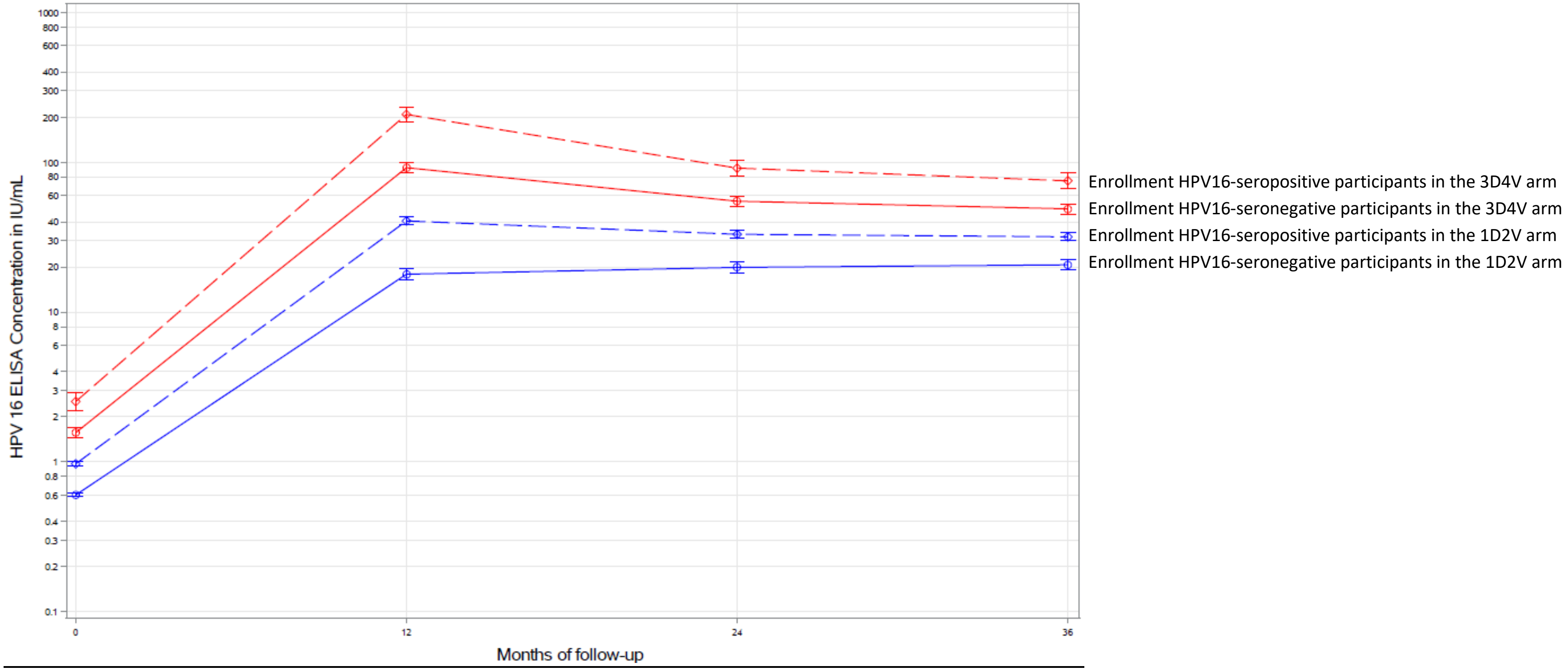

Legend. Women who received three doses of the quadrivalent HPV vaccine (3D4V) are in red and girls who received a single dose of the ASO4-adjuvanted HPV vaccine (1D2V) are in blue. Dashed lines represent participants who were HPV16 seropositive at the time of first HPV vaccination and solid lines represent participants who were HPV16 seronegative at the time of first HPV vaccination. Error bars represent 95% CI of GMC.

Supplementary Figure 6. HPV18 antibody levels 12, 24, and 36-months after initial HPV vaccination in the two trial arms, stratified by HPV serostatus at time of first HPV vaccination. Antibody concentration measured by ELISA.

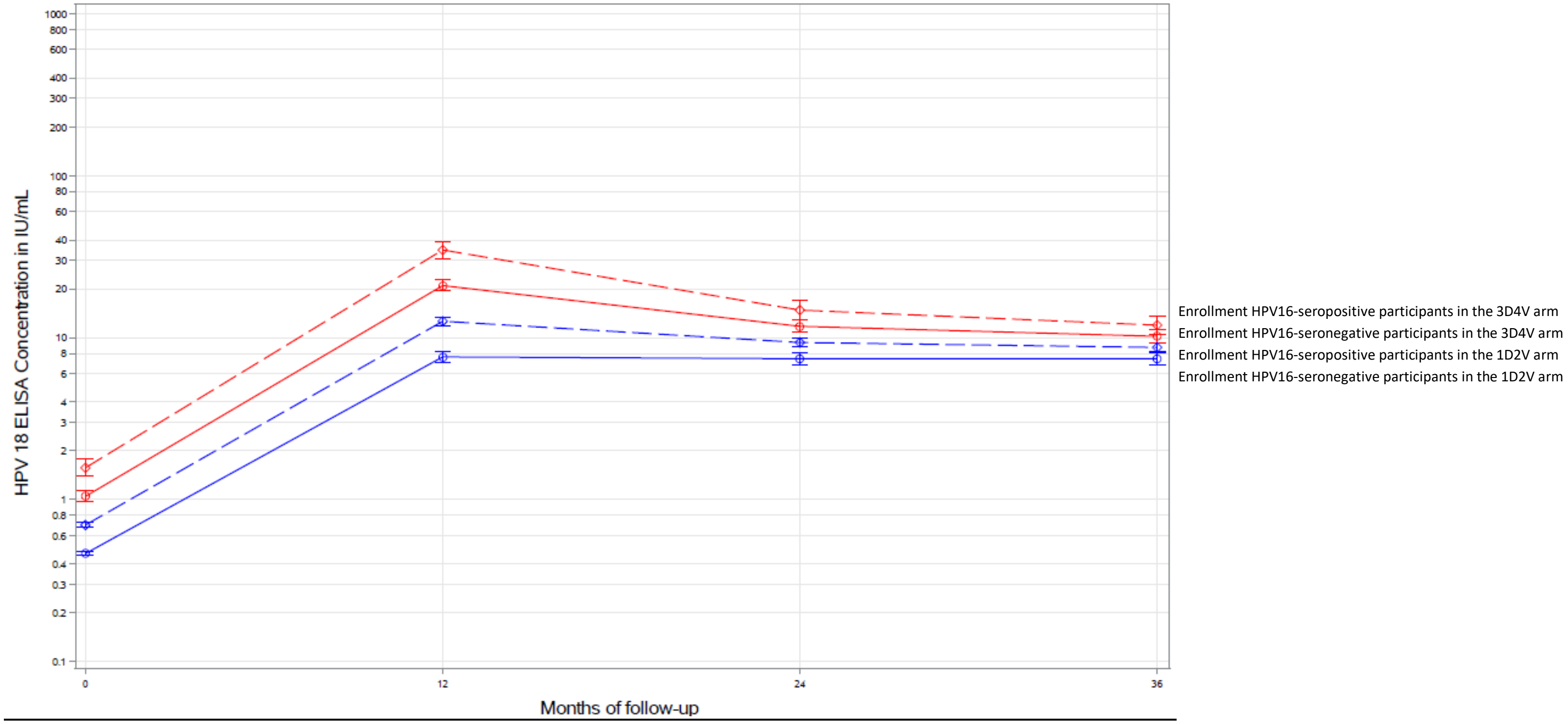

Legend. Women who received three doses of the quadrivalent HPV vaccine (3D4V) are in red and girls who received a single dose of the ASO4-adjuvanted HPV vaccine (1D2V) are in blue. Dashed lines represent participants who were HPV18 seropositive at the time of first HPV vaccination and solid lines represent participants who were HPV18 seronegative at the time of first HPV vaccination. Error bars represent 95% CI of GMC.

Supplemental Table 1. Serious adverse events and relatedness to HPV vaccination reported among girls and women during the active monitoring phase of the PRIMAVERA trial.

	Girls (N=620) N (%)				Women (N=620) N (%)			
	TOTAL	Not Related	Possibly Related	Definitely Related	TOTAL	Not Related	Possibly Related	Definitely Related
Autoimmune	0 (0.0)	0 (0.0)	0 (0.0)	0 (0.0)	1 (0.2)	1 (0.2)	0 (0.0)	0 (0.0)
Congenital abnormalities	0 (0.0)	0 (0.0)	0 (0.0)	0 (0.0)	0 (0.0)	0 (0.0)	0 (0.0)	0 (0.0)
Dermatological	0 (0.0)	0 (0.0)	0 (0.0)	0 (0.0)	0 (0.0)	0 (0.0)	0 (0.0)	0 (0.0)
Gastro-intestinal	1 (0.2)	1 (0.2)	0 (0.0)	0 (0.0)	0 (0.0)	0 (0.0)	0 (0.0)	0 (0.0)
Infections	0 (0.0)	0 (0.0)	0 (0.0)	0 (0.0)	3 (0.5)	3 (0.5)	0 (0.0)	0 (0.0)
Gynecological	0 (0.0)	0 (0.0)	0 (0.0)	0 (0.0)	1 (0.2)	1 (0.2)	0 (0.0)	0 (0.0)
Miscellaneous	0 (0.0)	0 (0.0)	0 (0.0)	0 (0.0)	1 (0.2)	1 (0.2)	0 (0.0)	0 (0.0)
Neurological	0 (0.0)	0 (0.0)	0 (0.0)	0 (0.0)	0 (0.0)	0 (0.0)	0 (0.0)	0 (0.0)
Obstetrics	0 (0.0)	0 (0.0)	0 (0.0)	0 (0.0)	7 (1.1)	7 (1.1)	0 (0.0)	0 (0.0)
Oncological	0 (0.0)	0 (0.0)	0 (0.0)	0 (0.0)	0 (0.0)	0 (0.0)	0 (0.0)	0 (0.0)
Psychiatrics	1 (0.2)	1 (0.2)	0 (0.0)	0 (0.0)	0 (0.0)	0 (0.0)	0 (0.0)	0 (0.0)
Skeletal/Muscular	0 (0.0)	0 (0.0)	0 (0.0)	0 (0.0)	0 (0.0)	0 (0.0)	0 (0.0)	0 (0.0)
Thyroid	0 (0.0)	0 (0.0)	0 (0.0)	0 (0.0)	0 (0.0)	0 (0.0)	0 (0.0)	0 (0.0)

Denominator includes the full study population. Note percentages are computed within each column.

Supplemental Table 2. HPV16 and 18 ELISA results at 36-months (final analysis) and 24-months (interim analysis) in the according to protocol analytical cohort comparing girls outside the age for routine HPV vaccination in Costa Rica in the 1D2V arm to women in the 3D4V arm. We evaluated study aims in this secondary analytical cohort of girls outside the age for eligibility in the National Vaccination Program as there was almost no possibility they would have received external HPV vaccination.

	N seropositive/ N in cohort	% Seropositive at 36 months (95%CI)	GMC (95%CI)	GMC ratio (96%CI)
**36 Months**				
**HPV16**				
11-14 Girls (1D Cervarix)	461/462	99.8 (98.9-100.0)	20.8 (19.0-22.8)	0.49 (0.42-0.56)
Women (3D Gardasil)	366/366	100.0 (99.2-100.0)	42.8 (38.8-47.2)	
**HPV18**				
11-14 Girls (1D Cervarix)	441/447	98.7 (97.2-99.5)	7.9 (7.2-8.7)	1.09 (0.93-1.27)
Women (3D Gardasil)	358/373	96.0 (93.6-97.6)	7.2 (6.4-8.1)	
**24 Months**	**N seropositive/ N in cohort**	**% Seropositive at 24 months (95%CI)**	**GMC (95%CI)**	**GMC ratio (99%CI)**
**HPV16**				
11-14 Girls (1D Cervarix)	463/464	99.8 (98.9-100.0)	20.0 (18.3-21.9)	0.41 (0.35-0.49)
Women (3D Gardasil)	371/371	100.0 (99.2-100.0)	48.8 (44.4-53.7)	
**HPV18**				
11-14 Girls (1D Cervarix)	440/448	98.2 (96.6-99.2)	7.6 (6.9-8.3)	0.86 (0.71-1.04)
Women (3D Gardasil)	367/376	97.6 (95.7-98.8)	8.8 (7.9-9.9)	

Supplemental Table 3. HPV16 and 18 ELISA results at 1- and 12-months post-HPV vaccination in the according to protocol analytical cohort comparing girls who were in and not in the age range for routine HPV vaccination in Costa Rica.

	**N seropositive/ N in cohort**	**% Seropositive at 36 months (95%CI)**	**GMC (95%CI)**	**GMC ratio (95%CI)**
**1 Month**				
**HPV16**				
9-10 Girls (1D Cervarix)	99/99	100.0 (97.0-100.0)	83.4 (71.2-97.8)	1.33 (1.12-1.58)
11-14 Girls (1D Cervarix)	461/461	100.0 (99.4-100.0)	62.6 (58.3-67.3)	
**HPV18**				
9-10 Girls (1D Cervarix)	100/100	100.0 (97.0-100.0)	32.8 (28.2-38.2)	1.29 (1.09-1.54)
11-14 Girls (1D Cervarix)	446/446	100.0 (99.3-100.0)	25.4 (23.3-27.6)	
**12 Months**	**N seropositive/ N in cohort**	**% Seropositive at 24 months (95%CI)**	**GMC (95%CI)**	**GMC ratio (95%CI)**
**HPV16**				
9-10 Girls (1D Cervarix)	85/85	100.0 (96.5-100.0)	25.0 (20.7-30.2)	1.48 (1.20-1.81)
11-14 Girls (1D Cervarix)	462/464	99.6 (98.6-99.9)	17.0 (15.5-18.6)	
**HPV18**				
9-10 Girls (1D Cervarix)	84/84	100.0 (96.5-100.0)	10.6 (8.8-12.9)	1.42 (1.15-1.75)
11-14 Girls (1D Cervarix)	440/4448	98.2 (96.6-99.2)	7.5 (6.9-8.2)	

Supplemental Table 4. HPV16 and 18 ELISA results at 36-months (final analysis) and 24-months (interim analysis) by arm in the according to protocol analytical cohort stratified by enrollment age

	N seropositive/ N in cohort	% Seropositive at 36 months (95%CI)	GMC (95%CI)	GMC ratio (96%CI)
**36 months**				
**HPV16**				p-het=0.079
9-11 Girls (1D Cervarix)	203/203	100.0 (98.5-100.0)	21.7 (19.1-24.6)	0.46 (0.38-0.56)
18-21 Women (3D Gardasil)	226/226	100.0 (98.7-100.0)	47.1 (41.5-53.4)	
12-14 Girls (1D Cervarix)	335/336	99.7 (98.5-100.0)	21.2 (19.0-23.6)	0.58 (0.48-0.70)
22-25 Women (3D Gardasil)	140/140	100.0 (97.9-100.0)	36.7 (31.7-42.5)	
**HPV18**				p-het=0.14
9-11 Girls (1D Cervarix)	199/199	100.0 (98.5-100.0)	8.1 (7.2-9.2)	1.01 (0.82-1.25)
18-21 Women (3D Gardasil)	201/212	94.8 (91.2-97.2)	8.0 (6.8-9.5)	
12-14 Girls (1D Cervarix)	318/324	98.1 (96.2-99.2)	7.9 (7.1-8.9)	1.24 (1.01-1.54)
22-25 Women (3D Gardasil)	157/161	97.5 (94.1-99.2)	6.4 (5.4-7.5)	
**24 months**	**N seropositive/ N in cohort**	**% Seropositive at 24 months (95%CI)**	**GMC (95%CI)**	**GMC ratio (99%CI)**
**HPV16**				p-het=0.28
9-11 Girls (1D Cervarix)	211/211	100.0 (98.6-100.0)	22.0 (19.3-25.0)	0.41 (0.33-0.52)
18-21 Women (3D Gardasil)	229/229	100.0 (98.7-100.0)	53.2 (47.1-60.1)	
12-14 Girls (1D Cervarix)	335/336	99.7 (98.5-100.0)	19.8 (17.8-22.0)	0.47 (0.37-0.60)
22-25 Women (3D Gardasil)	142/142	100.0 (97.9-100.0)	42.0 (36.2-48.7)	
**HPV18**				p-het=0.24
9-11 Girls (1D Cervarix)	205/206	99.5 (97.6-100.0)	8.4 (7.4-9.5)	0.85 (0.65-1.10)
18-21 Women (3D Gardasil)	208/213	97.7 (94.9-99.1)	9.9 (8.5-11.5)	
12-14 Girls (1D Cervarix)	316/324	97.5 (95.4-98.8)	7.6 (6.8-8.5)	1.00 (0.77-1.29)
22-25 Women (3D Gardasil)	159/163	97.5 (94.2-99.2)	7.6 (6.5-8.9)	

Supplemental Table 5. HPV16 and 18 ELISA results at 36-months (final analysis) and 24-months (interim analysis) by arm in the according to protocol analytical cohort stratified by enrollment month

	N seropositive/ N in cohort	% Seropositive at 36 months (95%CI)	GMC (95%CI)	GMC ratio (96%CI)
**36 months**				
**HPV16**				p-het=0.73
Girls (1D Cervarix) enrolled April-May	195/195	100.0 (98.5-100.0)	21.6 (18.8-24.8)	0.52 (0.43-0.64)
Women (3D Gardasil) enrolled April-May	145/145	100.0 (98.0-100.0)	41.6 (36.3-47.7)	
Girls (1D Cervarix) enrolled June-August	343/344	99.7 (98.6-100.0)	21.3 (19.2-23.6)	0.49 (0.41-0.58)
Women (3D Gardasil) enrolled June-August	221/221	100.0 (98.7-100.0)	43.6 (38.2-49.7)	
**HPV18**				p-het=0.15
Girls (1D Cervarix) enrolled April-May	182/184	98.9 (96.5-99.8)	7.9 (6.8-9.1)	1.26 (1.00-1.58)
Women (3D Gardasil) enrolled April-May	149/154	96.8 (93.0-98.8)	6.3 (5.3-7.4)	
Girls (1D Cervarix) enrolled June-August	335/339	98.8 (97.2-99.6)	8.1 (7.3-9.1)	1.01 (0.83-1.24)
Women (3D Gardasil) enrolled June-August	209/219	95.4 (92.0-97.7)	8.0 (6.8-9.4)	
**24 months**	**N seropositive/ N in cohort**	**% Seropositive at 24 months (95%CI)**	**GMC (95%CI)**	**GMC ratio* (99%CI)**
**HPV16**				p-het=0.95
Girls (1D Cervarix) enrolled April-May	196/196	100.0 (98.5-100.0)	20.4 (17.9-23.4)	0.43 (0.33-0.55)
Women (3D Gardasil) enrolled April-May	147/147	100.0 (98.0-100.0)	47.9 (41.8-54.8)	
Girls (1D Cervarix) enrolled June-August	350/351	99.7 (98.6-100.0)	20.7 (18.7-22.9)	0.42 (0.34-0.52)
Women (3D Gardasil) enrolled June-August	224/224	100.0 (98.7-100.0)	49.2 (43.4-55.9)	
**HPV18**				p-het=0.88
Girls (1D Cervarix) enrolled April-May	182/185	98.4 (95.7-99.6)	7.4 (6.5-8.5)	0.91 (0.69-1.19)
Women (3D Gardasil) enrolled April-May	154/155	99.4 (96.9-100.0)	8.2 (7.0-9.6)	
Girls (1D Cervarix) enrolled June-August	339/345	98.3 (96.4-99.3)	8.2 (7.4-9.2)	0.89 (0.69-1.14)
Women (3D Gardasil) enrolled June-August	213/221	96.4 (93.2-98.3)	9.3 (7.9-10.8)	

Supplemental Table 6. HPV16 and 18 ELISA results at 36-months (final analysis) and 24-months (interim analysis) by arm in the according to protocol analytical cohort stratified by enrollment region.

	**N seropositive/ N in cohort**	**% Seropositive at 36 months (95%CI)**	**GMC (95%CI)**	**GMC ratio (96%CI)**
**36 months**				
**HPV16**				p-het=0.46
Coastal Girls (1D Cervarix)	249/250	99.6 (98.0-100.0)	21.5 (19.0-24.3)	0.52 (0.43-0.64)
Coastal Women (3D Gardasil)	169/169	100.0 (98.2-100.0)	41.0 (35.7-47.1)	
Mountainous Girls (1D Cervarix)	289/289	100.0 (99.0-100.0)	21.4 (19.1-24.0)	0.48 (0.40-0.58)
Mountainous Women (3D Gardasil)	197/197	100.0 (98.5-100.0)	44.6 (38.9-51.0)	
**HPV18**				p-het=0.095
Coastal Girls (1D Cervarix)	240/244	98.4 (96.1-99.5)	8.5 (7.5-9.5)	1.27 (1.03-1.57)
Coastal Women (3D Gardasil)	157/163	96.3 (92.5-98.5)	6.7 (5.7-7.8)	
Mountainous Girls (1D Cervarix)	277/279	99.3 (97.7-99.9)	7.7 (6.8-8.7)	0.99 (0.80-1.23)
Mountainous Women (3D Gardasil)	201/210	95.7 (92.3-97.9)	7.7 (6.6-9.1)	
**24 months**	**N seropositive/ N in cohort**	**% Seropositive at 24 months (95%CI)**	**GMC (95%CI)**	**GMC ratio (99%CI)**
**HPV16**				p-het=0.30
Coastal Girls (1D Cervarix)	253/254	99.6 (98.1-100.0)	21.0 (18.5-23.7)	0.45 (0.36-0.58)
Coastal Women (3D Gardasil)	171/171	100.0 (98.3-100.0)	46.3 (40.5-53.0)	
Mountainous Girls (1D Cervarix)	293/293	100.0 (99.0-100.0)	20.3 (18.3-22.7)	0.40 (0.32-0.50)
Mountainous Women (3D Gardasil)	200/200	100.0 (98.5-100.0)	51.1 (44.8-58.4)	
**HPV18**				p-het=0.10
Coastal Girls (1D Cervarix)	242/247	98.0 (95.6-99.3)	8.4 (7.4-9.5)	1.02 (0.79-1.32)
Coastal Women (3D Gardasil)	161/164	98.2 (95.1-99.5)	8.2 (7.0-9.5)	
Mountainous Girls (1D Cervarix)	279/283	98.6 (96.6-99.5)	7.6 (6.7-8.5)	0.81 (0.63-1.04)
Mountainous Women (3D Gardasil)	206/212	97.2 (94.2-98.8)	9.4 (8.0-11.0)	

<u>Supplemental Table 7.</u> Sensitivity analysis: HPV16 and 18 ELISA results at 36-months (final analysis) and 24-months (interim analysis) by arm in the serocombined according to protocol analytical cohort.

	**N seropositive/ N in cohort**	**% Seropositive at 36 months (95%CI)**	**GMC (95%CI)**	**GMC ratio (96%CI)**
**36-month final analysis**				
**HPV16**				
Girls (1D Cervarix)	572/574	99.7 (98.9-99.9)	21.8 (20.0-23.6)	0.40 (0.35-0.45)
Women (3D Gardasil)	544/544	100.0 (99.5-100.0)	54.9 (50.1-60.3)	
**HPV18**				
Girls (1D Cervarix)	565/572	98.8 (97.6-99.5)	8.1 (7.5-8.8)	0.82 (0.71-0.95)
Women (3D Gardasil)	526/542	97.0 (95.4-98.2)	9.9 (8.9-11.0)	
**24-months interim analysis**	**N seropositive/ N in cohort**	**% Seropositive at 24 months (95%CI)**	**GMC (95%CI)**	**GMC ratio (99%CI)**
**HPV16**				
Girls (1D Cervarix)	580/582	99.7 (98.9-99.9)	20.9 (19.3-22.7)	0.33 (0.28-0.39)
Women (3D Gardasil)	550/550	100.0 (99.5-100.0)	63.6 (58.0-69.7)	
**HPV18**				
Girls (1D Cervarix)	570/580	98.3 (96.9-99.1)	8.0 (7.4-8.7)	0.68 (0.57-0.81)
Women (3D Gardasil)	539/548	98.4 (97.0-99.2)	11.8 (10.7-13.1)	

<u>Supplemental Table 8.</u> Sensitivity analysis: HPV16 and 18 ELISA results at 36-months (final analysis) and 24-months (interim analysis) by arm in the modified intention to treat analytical cohort.

	**N seropositive/ N in cohort**	**% Seropositive at 36 months (95%CI)**	**GMC (95%CI)**	**GMC ratio (96%CI)**
**36-month final analysis**				
**HPV16**				
Girls (1D Cervarix)	573/575	99.7 (98.9-99.9)	21.7 (20.0-23.6)	0.40 (0.35-0.45)
Women (3D Gardasil)	548/548	100.0 (99.5-100.0)	54.8 (50.0-60.1)	
**HPV18**				
Girls (1D Cervarix)	568/575	98.8 (97.6-99.5)	8.2 (7.5-8.9)	0.83 (0.72-0.95)
Women (3D Gardasil)	531/548	96.9 (95.2-98.1)	9.9 (8.9-11.0)	
**24-months interim analysis**	**N seropositive/ N in cohort**	**% Seropositive at 24 months (95%CI)**	**GMC (95%CI)**	**GMC ratio (99%CI)**
**HPV16**				
Girls (1D Cervarix)	581/583	99.7 (98.9-99.9)	20.9 (19.3-22.6)	0.33 (0.28-0.38)
Women (3D Gardasil)	554/554	100.0 (99.5-100.0)	63.7 (58.2-69.7)	
**HPV18**				
Girls (1D Cervarix)	573/583	98.3 (97.0-99.1)	8.1 (7.4-8.8)	0.68 (0.58-0.81)
Women (3D Gardasil)	545/554	98.4 (97.0-99.2)	11.8 (10.7-13.1)	

Supplemental Table 9. GMC ratios of HPV16/HPV18 at 24-months after initial HPV vaccination by trial, age, dose and vaccine product.

Study	Age (years)	Dose & Vaccine product	GMC (IU/ml)		Ratio
			HPV 16 (95% CI)	HPV 18 (95% CI)	(HPV16/HPV18)
DoRIS[1]	9-14	1-dose Cervarix®	23 (20-26)	10 (9-11)	2.3
	9-14	2-dose Cervarix®	163 (141-188)	50 (43-58)	3.3
	9-14	3-dose Cervarix®	412 (357-475)	107 (90-126)	3.9
	9-14	1-dose Gardasil-9®	14 (12-16)	6 (5-7)	2.3
	9-14	2-dose Gardasil-9®	125 (107-146)	29 (25-35)	4.3
	9-14	3-dose Gardasil-9®	118 (102-137)	32 (27-38)	3.7
KENSHE[2]	15-20	1-dose Cervarix®	25.3 (21.0-30.6)	9.7 (7.6-12.4)	2.6
	15-20	1-dose Gardasil-9®	9.5 (7.8-11.5)	3.9 (3.2-4.8)	2.4
PRIMAVERA[3]	9-14	1-dose Cervarix®	20.6 (19.0-22.4)	7.9 (7.3-8.6)	2.6
	18-25	3-dose Gardasil®	48.8 (44.4-53.7)	8.8 (7.9-9.9)	5.5

GMC, Geometric mean concentration; IU/ml, International Units per milliliter

[1] 24-month GMC data abstracted from Table 3 in Watson-Jones et al. (*Lancet Glob Health* 2022).
[2] 24-month GMC data abstracted from Table 2 in Baisley et al. (*Lancet Glob Health* 2024).
[3] 24-month GMC from Table 1 in PRIMAVERA manuscript.

Title: Non-inferiority trial comparing immunogenicity from 1-dose of bivalent HPV vaccine in girls to 3-doses of quadrivalent vaccine in women: The PRIMAVERA-ESCUDDO Trial ("Puente de Respuesta Inmunológica para Mejorar el Acceso a Vacunas y ERrAdicar el cancer")

Study Phase: Phase IIIb

Study Vaccines: The FDA approved bivalent HPV16/18 virus-like particle with AS04-adjuvant vaccine (Cervarix) and the FDA approved HPV6/11/16/18 virus-like particles with Alum-adjuvant vaccine (Gardasil)

Candidate regimen: One dose of Cervarix

Control regimen: Three doses of Gardasil

NCI Protocol number: 19-C-009

NCI Principal Investigator: Aimée R. Kreimer PhD (NCI) on behalf of the PRIMAVERA-ESCUDDO Study team

Costa Rica Principal Investigator: Bernal Cortes PharmDr (ACIB-FUNIN) on behalf of the PRIMAVERA-ESCUDDO Study team

Study Sponsor: US National Cancer Institute

Protocol Version/Date:
Version 1.0: 30 October 2018
Version 1.1: 28 November 2018
Version 1.2: 13 December 2018
Version 1.3: 13 March 2019
Version 1.4: 20 June 2019
Version 1.5: 15 June 2022
Version 1.6: 28 February 2023

PRIMAVERA-ESCUDDO Study Team:

US National Cancer Institute, National Institutes of Health

Division of Cancer Epidemiology and Prevention
Mitchell H. Gail
Allan Hildesheim
Aimée R. Kreimer
Ligia Pinto
Danping Liu

Center for Cancer Research
Doug Lowy
John Schiller

Agencia Costarricense de Investigaciones Biomédicas (ACIB) – Fundación INCIENSA
Bernal Cortes
Paula Gonzalez (deceased)
Carolina Porras
Byron Romero
Ariane Castrillo

International Agency for Research on Cancer
Rolando Herrero

Participating Institutions:
US National Cancer Institute, National Institutes of Health, located at 9609 Medical Center Drive, Bethesda, MD 20892, USA.

Agencia Costarricense de Investigaciones Biomédicas (ACIB) – Fundación INCIENSA, located at Solarium, frente Aeropuerto Daniel Oduber, bodega 8C, Liberia, Liberia, Guanacaste, 50101 Costa Rica.

International Agency for Research on Cancer, World Health Organization, located at 150 Cours Albert Thomas 69372 Lyon, France

LIST OF ABBREVIATIONS

ACIB	Agencia Costarricense de Investigaciones Biomédicas
AS04	Adjuvant system 04 (Monophosphoryl lipid A and aluminum phosphate)
ATP	According to protocol
BCR	B cell receptor
°C	Degrees Centigrade
CDC	U.S. Centers for Disease Control and Prevention
CI	Confidence interval
CIN	Cervical intraepithelial neoplasia
CIN2/3	Cervical intraepithelial neoplasia grades 2/3, adenocarcinoma *in situ*, and invasive cervical cancer
PRIMAVERA	Puente de Respuesta Inmunológica para Mejorar el Acceso a Vacunas y ERrAdicar el cancer (PRIMAVERA-ESCUDDO)
CVT	Costa Rica HPV Vaccine Trial
DCEG	Division of Cancer Epidemiology and Genetics
DSMB	Data and Safety Monitoring Board
eCRF	Electronic Case Report Form
ELISA	Enzyme-linked immunoabsorbent assay
EMA	European Medicines Agency
ESCUDDO	A scientific evaluation of one or two doses of vaccine against human papillomavirus: the ESCUDDO study ("Estudio de Comparacion de Una y Dos Dosis de Vacunas Contra el Virus de Papiloma Humano (VPH)")
ERP	DCEG Ethics Review Panel
FDA	U.S. Food and Drug Administration
FWA	Federal-wide assurance
GCP	Good clinical practices
GEE	Generalized estimating equation
GMT	Geometric mean titer
GSK	GlaxoSmithKline
HPV	Human papillomavirus
IARC	International Agency for Research on Cancer
ICD	International Classification of Diseases
IgG	Immunoglobulin G
IgM	Immunoglobulin M
IOM	Institute of Medicine
IRB	Institutional Review Board
ITT	Intent to treat
L1	Papillomavirus structural proteins
LLPC	Long lived plasma cells
LMIC	Low to middle income country
MD	Maryland, USA or Doctor of Medicine
mL	Milliliter

MPL®	Monophosphoryl lipid A (Corixa Corporation, Seattle, Washington, USA [Formerly Ribi])
NCI	U.S. National Cancer Institute
NIH	U.S. National Institutes of Health
OW	Outreach worker
PAHO	Pan American Health Organization Technical Advisory Group
PATRICIA	PApilloma TRIal against Cancer In young Adults
PID	Participant study identification
RBC	Red blood cells
RCT	Randomized controlled trial
SAE	Serious adverse event
SAG	Scientific Advisory Group
SAGE	Strategic Advisory Group of Experts
SAS	Statistical Analysis System
SEAP-NA	Secreted alkaline phosphatase neutralization assay
SOP	Standard operating procedure
TLR-4	Toll-like receptor 4
US or USA	United States of America
VE	Vaccine Efficacy
VAERS	Vaccine Adverse Events Reporting System
VLP	Virus-like particle
WHO	World Health Organization

TABLE OF CONTENTS

TABLE OF CONTENTS **29**

SUMMARY **32**

Background and Rationale **34**

Evidence and biological rationale supporting protection by a single dose of the HPV vaccine 36

Rationale for the proposed Immunobridging Study 37

Antibody response to HPV vaccination is dependent on number of doses, time since last dose, vaccine product, and age and serostatus at vaccination 38

Expectation of Non-inferiority in Immunobridging Study 41

STUDY OBJECTIVES **42**

Primary Objective 43

Secondary Objectives 44

STUDY DESIGN AND METHODS OF TRIAL **44**

Overview 44

Selection of Study Vaccines 46

Selection of Gardasil (and not Gardasil9) as control vaccine product 47

Selection of HPV 16 and HPV 18 antibody levels as outcome 47

Target populations 47

Costa Rica as the study setting 48

Specimen collection 48

Eligibility criteria 48

Inclusion criteria 48

Exclusion criteria 49

Deferral criteria at enrollment: 49

Contraindications to subsequent vaccination (Gardasil group) 49

Concomitant Medications/Vaccinations 50

Outreach and Enrollment Visits for Both Cervarix and Gardasil Groups (0 months) 50

Gardasil Group Followup Vaccination Visits (2 and 6 months) 51

Active Follow-up of Both Groups (Months 1, 12, 24, and 36) 52

Home Visits During Follow-up 52

Procedures and data collection 52

Medical history 52
Assessment of HPV vaccination outside the research study 53
Urine Samples (Vaccination Visits only) 53
Blood collection 53
Vaccine Procedures 54
Blood preparation, storage and shipment 54
Expected Adverse Events and Adverse Event Monitoring and Reporting 54
Protocol Deviation Monitoring and Reporting 56
Data collection and electronic records 56
Serology 57
Efforts to maximize participation and retention 58
Approval of the exiting of participants from the study 59
ANALYSIS PLAN 59
Primary Analytical Cohort 59
Immunogenicity Endpoint 60
Analysis 60
Power and Sample Size Considerations 61
HUMAN SUBJECTS PROTECTIONS 62
Institutional Approvals 62
Justification for exclusions and dose schedule 62
Safeguards for vulnerable populations 63
Consent process and documentation 65
Subject privacy and confidentiality of data, including plans for sharing, future use, and disposition of study materials/biologic specimens 67
Plan for return of individual study results to participants 67
Evaluation of anticipated benefits and risks/discomforts 68
Compensation 69
ETHICAL CONSIDERATIONS AND TRIAL OVERSIGHT 69
Data and Safety Monitoring 69
Delivering trial results to participants and actions if study shows one dose failure. 69
Working Group 70

Regulatory Body Review 70

DATA SHARING PLAN 70

REFERENCES 72

NCI IRB/ERP AMENDMENTS AND APPROVAL DATES 77

SUMMARY

Human papillomavirus (HPV) vaccination successfully prevents targeted HPV infections, related precancerous lesions, and, ultimately, cervical and other HPV-related cancers. Despite the established efficacy of the HPV vaccine, most girls living in areas with the greatest risk for cervical cancer are not being vaccinated. The cost and logistical difficulties of vaccinating girls with the recommended *multiple-dose* administration has been a significant impediment to preventing this often-fatal cancer.

The goal of this research is to provide convincing and actionable evidence for regulatory bodies that a single dose of the HPV vaccine will elicit an immune response sufficient to protect against targeted HPV infections and subsequent neoplasms. This study will provide earlier and complementary results to the definitive 24,000-girl study evaluating the vaccine efficacy for a 1-dose regimen against virologic outcomes, which is underway in Costa Rica and is scheduled to be completed in 2024/5. That study, called ESCUDDO, is being conducted by the U.S. National Cancer Institute (NCI) and the Agencia Costarricense de Investigaciones Biomédicas (ACIB).

Our specific objective is to show that the immune response for 1-dose of the bivalent vaccine Cervarix in girls 9-14 years old is non-inferior to the immune response for 3-doses of the quadrivalent vaccine Gardasil in women 18-25, a dosage/population combination with demonstrated efficacy. We emphasize that we are evaluating a single dose using the more immunogenic vaccine (i.e. Cervarix) in the population (i.e. 9-14-year-old girls) that has the stronger immune response. Therefore, despite a 1-dose regimen eliciting lower titers than a 3-dose regimen when using the same vaccine and population, we hypothesize that 1-dose of Cervarix in girls will be non-inferior to 3-doses of Gardasil in women. We would expect that a successful demonstration of non-inferiority will provide a direct regulatory pathway for label change, which would in turn promote early adoption of and preparation for 1-dose vaccine programs by relevant agencies (i.e. WHO). We note that new vaccines or new administrations of a vaccine have, in the past, been recommended or approved for licensure by demonstrating that the immune response for the new vaccination plan is non-inferior to the immune response from an existing, approved vaccination plan. For the HPV vaccine, these immunobridging studies have informed the recommendation for a two-dose administration, and vaccination in 9-14 year old girls and boys. Scientifically, this study can provide additional evidence that subunit vaccines with repetitive structures elicit sufficient antibody responses to confer protection and will demonstrate the importance of the adjuvant system to maximize plateau titer levels.

From this effort, we anticipate one of two outcomes, which are summarized below along with their implications.

Outcome #1: The HPV-16 and HPV-18 antibody levels at 24 and/or 36 months from 1-dose of Cervarix in girls 9-14 years old are non-inferior to 3-doses of Gardasil in women 18-25 years old. The implication from this result is that 1-dose of Cervarix will be efficacious for protecting against incident HPV 16/18 infection and associated precancerous lesions. The result could lead to provisional approvals supporting a 1-dose regimen of the bivalent HPV vaccine by international and regulatory agencies (e.g., WHO, FDA, and EMA), contingent on the definitive corroboration of efficacy from ESCUDDO, which will deliver its results three to four years later.

Outcome #2: The HPV-16 and/or HPV-18 antibody levels at 24 and 36 months from 1-dose of Cervarix in girls 9-14 years old cannot be demonstrated to be non-inferior to 3-doses of Gardasil in women 18-25 years old. The implication from this result would be that the vaccine efficacy of a single dose will need to be directly evaluated by demonstration of protection against pertinent virologic outcomes, as planned in our ongoing 1-dose ESCUDDO study.

Background and Rationale

Persistent infection with carcinogenic genotypes of the human papillomavirus (HPV) is a necessary causal event in cervical carcinogenesis (1, 2). HPV exposure typically begins when individuals become sexually active, reaching peak incidence during the third decade of life (3). More than one half of carcinogenic HPV infections clear spontaneously within a year and approximately 90% resolve within several years (4, 5); however, infections that persist may progress to cancer precursors, and then to invasive cancer, typically after more than a decade (5). Worldwide, there are approximately 530,000 cervical cancers diagnosed annually and 265,000 deaths, 88% of which occur in low income nations, where cervical cancer is the leading cause of cancer mortality among women (6). Approximately 50% of cervical cancer is caused by HPV16, an additional 20% by HPV18, and the remaining 30% by 10 other carcinogenic types collectively (7). Accordingly, preventing carcinogenic HPV infections, especially HPV16 and 18, would dramatically reduce cervical cancer incidence and mortality. In addition, reducing HPV16 infections would eliminate a substantial proportion of vulvar, vaginal, anal, penile, and oropharyngeal cancers (1, 2).

There are currently three available HPV vaccines: Cervarix® (GlaxoSmithKline, Rixensart, Belgium) directed against HPV16 and 18, Gardasil® (Merck & Co., Whitehouse Station, NJ) directed against HPV16, HPV18 and the two HPV types causing genital warts, HPV6 and 11, and Gardasil-9® (Merck & Co., Whitehouse Station, NJ) directed against HPV 16, 18, 6, 11, 31, 33, 45, 52, and 58. Three doses of the commercially available HPV vaccines were demonstrated to be highly efficacious in preventing targeted carcinogenic HPV infections and related cervical cancer precursors and cancers. The vaccines also protect against infection at other sites at which HPV-related cancers develop (8-10). The HPV vaccines contain non-infectious, non-carcinogenic, virus-like particles (VLPs) consisting of L1 protein, the primary structural component of the HPV capsid. In addition to differences in the HPV types covered, Merck vaccines employ an aluminum hydroxyphosphate sulfate adjuvant, whereas the Cervarix adjuvant is AS04 (monophosphoryl lipid A and aluminum phosphate), which acts as a toll-like receptor-4 agonist that stimulates innate and adaptive immune responses and yields a significantly higher antibody response compared to the quadrivalent HPV vaccine (11).

The bivalent and quadrivalent HPV vaccines have demonstrated high efficacy against cervical intraepithelial neoplasia (CIN) grades 2/3 (CIN2/3) (cervical precancer) attributable to HPV16 and HPV18 when administered as a three-dose series in randomized, double-blind, multi-centric phase III trials conducted among women ages 15-26 years (12-14). Based on immunologic non-inferiority, regulatory agencies have approved and recommended a two-dose schedule of these vaccines (15-18). Specifically, the FDA, EMA, and WHO approvals and

recommendations for two-dose administrations came between December 2013 and October 2016 (15-19).

Analyses of population-level data accumulated in the first five years following implementation of an organized nationwide vaccination program with the quadrivalent HPV vaccine in Australia indicate that rates of condyloma and CIN have rapidly declined, providing an early indicator of vaccine effectiveness, together with evidence of herd immunity (20-23). Similarly, population-level data from Scotland accumulated in the first seven years following implementation of an organized nationwide vaccination program with the bivalent HPV vaccine show prevalence of HPV vaccine type and non-vaccine type, as well as CIN2+, are dropping precipitously in both vaccinated and unvaccinated women (i.e.: demonstration of herd immunity) (24). Consequently, the WHO states that the currently-available HPV vaccines provide similar protection against cervical cancer, and all girls in the recommended age range should be vaccinated with two doses of any of the HPV vaccines (19). Further, the WHO and the CDC provide ongoing monitoring of the HPV vaccines for safety-related outcomes: they report that HPV vaccines have similar safety profiles to other licensed vaccines (25, 26).

HPV vaccination of children prior to sexual initiation has been implemented progressively in high-income countries (and now in many middle-income countries, particularly in Latin America) since the vaccines were first licensed in 2006. More recently, a few low-income countries, such as Bhutan and Rwanda, adopted similar vaccination strategies for pre-adolescents. Yet, while HPV vaccines are currently licensed for use in over 100 countries, active vaccination programs are limited to about 50 countries, most of which are high-income countries (27). Costs and logistical challenges pose barriers to administration of a complete three-dose, or even a two-dose series, especially in countries with limited resources but high cervical cancer burden. In Costa Rica, the three HPV vaccines have been licensed by the Ministry of Health and, following support by the National Immunization Commission, were included in the National Immunization Program (under the Social Security System) starting in 2019. They implemented a school-based approach that administers two doses of the quadrivalent HPV vaccine to girls in fourth grade (typically age 10; one birth cohort per calendar year).

Scope of the Problem. Globally, fewer than 3% of age-eligible girls in low-income countries receive even a single dose of the HPV vaccines (27). Projecting current cervical cancer incidence and mortality trends in the face of low vaccine uptake, deaths from cervical cancer will increase dramatically in low-and middle-income countries over the next several years (28). Even in some wealthier nations, completion of even a two-dose vaccine series continues to be a problem. Thus, there is an urgent need to expedite the evaluation of a single dose of the prophylactic

HPV vaccine, to present the resulting evidence to regulatory and/or recommending bodies, and ultimately to ensure that more girls receive the vaccine.

Evidence and biological rationale supporting protection by a single dose of the HPV vaccine

The first line of evidence to support that HPV vaccines will induce long term protection after a single dose is the finding that the vaccines consistently induce strong and durable neutralizing antibody responses against the targeted types among individuals who received only a single dose. For Cervarix, the GMT antibody titers after four years were only four-fold lower in one dose compared to three dose recipients (29), and this ratio has been maintained out to seven years (30). For Gardasil, a similar four-fold difference between the antibody levels in the one and three dose recipients were reported in a four-year interim analysis (31). Note that we focus on the plateau levels of VLP antibodies and not peak titers immediately after the booster dose because the vast majority of exposures to sexually transmitted HPV infections will occur when vaccinated females are in the plateau phase. Further, in assessments of single- and multi-dose regimens of the HPV vaccines, peak titers do not predict plateau titers. Presently, direct comparison between the levels induced by different vaccine products among one-dose recipients is not possible because of the differences in study design, populations, and serologic assays used.

The second line of evidence that supports the hypothesis that a single dose of the HPV vaccine(s) may be sufficient is from post-hoc analyses of non-randomized groups from RCTS. In these studies, the observed vaccine efficacy (VE) against incident, persistent HPV 16/18 infections appears to be independent of the number of doses. For Cervarix, after four years of the follow-up in the Costa Rica Vaccine Trial, VE against incident infection by HPV16/18 for three doses was estimated to be 87% (95% CI=77 to 89%), while the VE for one dose was non-significantly different (VE 100%; 95% CI=79-100%) (29). Similarly, high VE in both arms was observed at 7 years (30) and in the PATRICIA study (29). For Gardasil, after 7 years of follow-up in the India HPV vaccine trial, the frequencies of cumulative incident HPV 16 and 18 infections were similar and uniformly low regardless of the number of doses; the frequencies of HPV 16 and 18 infections among the 1,180 women who received three doses was 0.9% and among the 1,823 women who received one dose was 1.6%, compared to a frequency of 6.2% in 1,481 unvaccinated women (32).

The exceptional immunogenicity of HPV vaccines can largely be attributed to the structure of the HPV vaccine antigen. Typical subunit vaccines are composed of monomeric or low valency multimeric proteins or carbohydrate/protein conjugates. In contrast, HPV VLPs are composed of 360 ordered protein subunits that form a particulate 55nm structure displaying a repetitive array of epitopes on their surface. The engagement of these repetitive elements by the B cell receptors (BCRs) on naïve B cells is believed to transmit exceptionally strong activation and

survival signals to the cells leading to consistent induction of memory B cells, and, most importantly, long lived plasma cells (LLPCs) that continuously produce antibodies for many years (33). Epitope spacing of 50-100Å appears to be critical. This spacing is commonly found on microbial surfaces but not on body surfaces normally exposed to the systemic immune system. The particulate and repetitive structure of VLPs likely contributes to B cell immunity in several additional ways. Particles of this size efficiently traffic to lymph nodes and are also efficiently phagocytized by antigen presenting cells for the initiation of immune responses and the generation of cognate T helper cells. The poly-valency of VLPs also leads to stable binding of nature low avidity IgM and complement, promoting their acquisition by follicular dendritic cells, which are key components in inducing humoral immune responses in the lymph node. The hepatitis B vaccine is also considered a VLP but appears to be much less immunogenic after a single dose, perhaps because it has a fewer subunits and/or because they float in a lipid bilayer. To summarize, by mimicking the key structural features of authentic viruses, the HPV VLPs consistently induce potent and long lasting humoral responses that more closely resemble the anti-virion responses to an acute virus infection or a single dose of a live-attenuated virus vaccine than the response to simple subunit vaccines (34).

Rationale for the proposed Immunobridging Study

There are pressing reasons to demonstrate that a single dose of the HPV vaccine is efficacious. These reasons led to the NCI's large-scale trial entitled "A scientific evaluation of one or two doses of vaccine against human papillomavirus: the ESCUDDO study" (clinicaltrials.gov identifier: NCT03180034). However, the results from this definitive study will not be available until 2024/2025. There would be significant benefits to provide prospective evidence with a controlled trial prior to 2024 that 1-dose should be efficacious. Furthermore, this proposed study offers a complementary approach, through immunobridging, to obtaining a one-dose HPV vaccine indication, if warranted by the data.

Indeed, the FDA and EMA have approved multiple changes to the vaccine label based on immunologic non-inferiority assessments in immunobridging studies (35). Specifically, the regulatory agencies (i.e. FDA (15, 36, 37) and EMA (8, 15-17, 38)) approved, and the international agencies (i.e. CDC (26) and WHO (19)) recommended, two-doses of the vaccination for boys and girls between the ages of 9 to 14. These changes to the vaccine label— for two-doses (39, 40), a younger age (41-45), and boys (45, 46) — were based on studies demonstrating that the antibody levels, as measured by ELISA, at one month following the last vaccination in the new population or new administration were non-inferior to levels among women 15-25 years old, the 'efficacy population', at one month following the third vaccination (47, 48).

The proposed study will evaluate a single dose of a highly immunogenic vaccine (i.e. Cervarix) in a population (i.e. 9-14-year-old girls) that has the stronger immune response and compare that to the antibody levels in a population with demonstrated efficacy. We now explain how, despite a 1-dose regimen eliciting lower titers than a 3-dose regimen when using the same vaccine and population, we hypothesize that 1-dose of Cervarix in girls will be non-inferior to 3-doses of Gardasil in women.

Antibody response to HPV vaccination is dependent on number of doses, time since last dose, vaccine product, and age and serostatus at vaccination

HPV 16/18 antibody levels induced by the HPV vaccines depend on the number of doses, the time since the vaccine was administered, the vaccine product, and the age and serostatus at initial HPV vaccination. We first discuss the relationship between antibody levels and each of these characteristics. We then show that, based on these relationships, it is reasonable to expect that antibody levels at 36 months after vaccination should be similar in 9 to 14 year old girls receiving a single dose of Cervarix and 18 to 25 year old women receiving three doses of Gardasil. This conclusion is the cornerstone of our proposed study.

Time since last dose: Among the women who receive three doses of Gardasil, the antibody levels peak immediately following the third vaccination and then decline to a more stable plateau. The boosting of the primary response generates a transient burst of antibodies, presumably due to the induction of short-lived plasma blasts from memory B cells, which transiently exaggerates the contribution of the booster doses to antibody titers.

Einstein and colleagues (49) show that, among approximately 100 women 18-26 year old who received three doses of Gardasil, the GMTs for HPV 16 antibodies decrease from 10053 at 7 months after the initial vaccination to 3265, 1183, 894, 653, and 750 at 12, 18, 24, 36, and 48 months after vaccination. Similarly, the GMTs for HPV 18 antibodies decrease from 2258 at 7 months to 596, 249, 175, 128, and 139 at 12, 18, 24, 36, and 48 months after vaccination.

We emphasize that no similar peak is observed after the first dose of Cervarix (Figure 1). To illustrate this, in the Costa Rica HPV Vaccine Trial (CVT) among women who received three doses, the HPV16 GMT one-month post dose one of Cervarix was 598; this increased after the second and third doses to 2035 at 12 months, and then decreased to 1115, 899, and 748 at 24, 36, and 48 months. For HPV18, the 1-month GMT was 224, which increased following the second and third dose to 826 at 12 months, and then decreased to 470, 369, and 334 at similar time points (n.b. antibody titers cannot be compared between studies due to profound differences in immunologic assays). In contrast, among women who received only a single dose, the GMTs for HPV 16 antibodies at 12 months after the first vaccination was 114 and remained stable at 124, 135, and 137 at 24, 36, and 48 months (Figure 1; Table 1). Among these same

women, the GMTs for HPV 18 antibodies at 12, 24, 36, and 48 months after vaccination was 70, 69, 73, and 70 (50). Given the association with time since vaccination and antibody titer, our evaluation is focused on antibody levels in the plateau phase (at least 24 or 36 months after vaccination), and not the peak titers, as we believe these are more relevant and predictive of long-term vaccine protection.

Number of Doses: The immune response to HPV vaccination increases with the number of doses. At 36 months after the initial vaccination in CVT, the GMTs for HPV-16 and HPV-18 antibody levels were respectively 6.6x (95% CI = 4.8-9.0) and 4.8x (3.5-6.4) higher among women who received 3-doses of Cervarix as compared to 1-doses (50). At 24 months in CVT, the GMTs for HPV-16 and HPV 18 were **9.1x** (6.5-12.5) and 6.9x (5.0-9.4) higher, respectively, in 3-dose Cervarix recipients (50).

Vaccine Product: Cervarix elicits a higher immune response because of the AS04 adjuvant in its formulation. At 36 months after the initial vaccination, Einstein and colleagues demonstrated that the GMTs for HPV-16 and HPV-18 were 5.9x (3.9-9.4) and 12.5x (8.0-19.4) higher among women receiving 3-doses of Cervarix as compared to 3-doses of Gardasil (49). At 24 months after the initial vaccination, the GMTs for HPV-16 and HPV-18 were **5.8x** (4.0-8.5) and 9.4x (6-14) higher among women receiving 3-doses of Cervarix as compared to 3-doses of Gardasil (49). Although less relevant for our purposes because they compared Cervarix and Gardasil within three to six months after the third vaccination, two other studies demonstrated GMTs for HPV-16 and HPV-18 were 3.3 to 6.6x higher among women who received Cervarix (51, 52).

Age: The VLP antibody response tends to be higher at younger ages. Unfortunately, there are no available data that directly compares HPV antibody levels at 36 months after the single and only dose of Cervarix between 9-14 year old girls and 18-25 year old women. Instead, we discuss results from related studies evaluating the general relationship between age and immune response.

At 36-months post-dose 1 among 3-dose recipients, the GSK trial HPV-012 found that GMTs for antibodies elicited by Cervarix to HPV-16 and HPV-18 were, respectively, 2.4x (1.6-3.5) and 1.9x (1.3-2.8) higher in girls 10-14 years old as compared to girls/women 15-25 years old (53). Similarly, Einstein and colleagues (49) demonstrated that GMTs for HPV 16 and HPV 18 antibodies were 2.0x (1.3-3.1) and 1.7x (1.1-2.6) higher among 18-26 year olds as compared to 27-35 year olds, again at 36 months among 3-dose recipients of Cervarix, with similar findings for Gardasil (Table 1).

At peak levels one-month after the third dose of Gardasil, Block et al (43) demonstrated that GMTs for HPV-16 and HPV-18 were, respectively, **1.8x** (1.5-2.2) and 2.0x (1.7-2.4) higher in girls 10-15 years old as compared with girls/women 16-23 years old.

When focusing on girls within the 9-14 year old range, there is no evidence to suggest that antibody levels dramatically increase at menarche. Reisenger and colleagues (54) reported that GMTs for HPV-16 and HPV-18 were 1.5x and 1.6x higher in girls 9-12 years old as compared to girls 13-15 years old at 1 month after last vaccination among 3-dose recipients of Gardasil. Others (unpublished data, personal communication) have similarly found that the immune response is similar pre/post-menarche.

HPV serostatus at time of initial vaccination: Finally, HPV serostatus at the time of HPV vaccination (i.e.: due to natural infection) does impact vaccine-induced plateau level. Petersen et al (55) reported that, among women who received three doses of Gardasil-9 in the per protocol population, HPV16 antibody titers 36-months after initial vaccination were 3.2x lower compared to HPV16 seropositive/HPV16 DNA negative women. This latter group comprised ~12% of the total study population and were excluded in the evaluations for licensure of the Merck HPV vaccines.

Figure 1. HPV 16 and 18 GMTs by months and number of doses of Cervarix (adapted from ref (50))

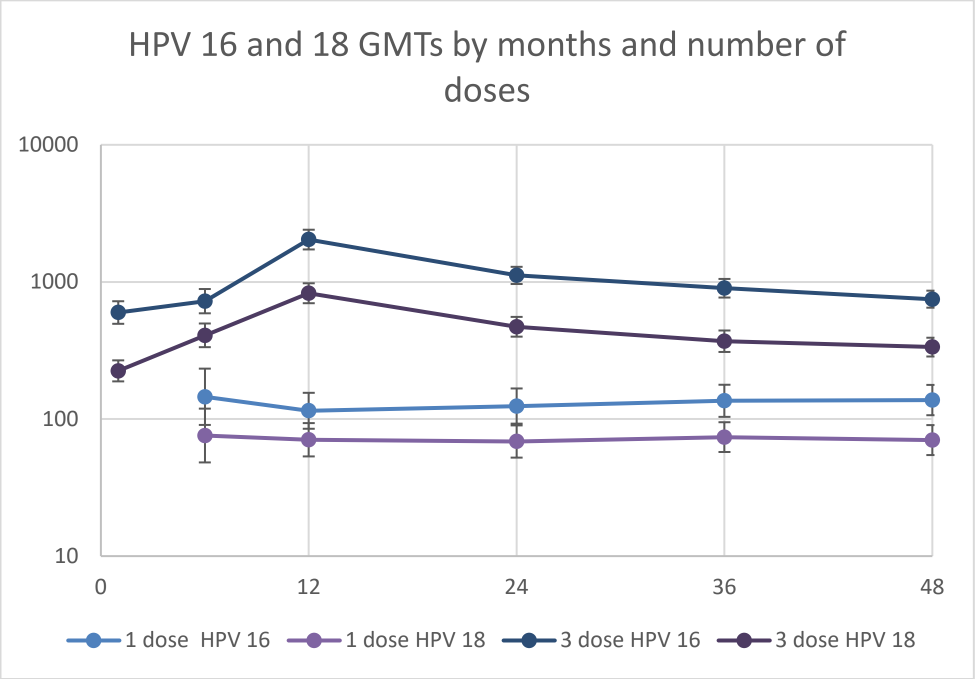

Table 1. GMT ratios of the antibody response to HPV vaccination by number of doses administered, the time since last dose, the vaccine product, and age and serostatus at first vaccination.

HPV type	Vaccines (Source)	# of doses	Comparison groups	# of months post vaccination: 7 Ratio (95% CI)	24 Ratio (95% CI)	36 Ratio (95% CI)
**Studies comparing GMTs between number of vaccine doses**						
HPV-16	Cervarix †	1, 3	3 doses vs. 1	9.1 (6.5-12.5) †	9.1 (6.5-12.5) †	6.6 (4.8-9.0) †
HPV-18	Cervarix †	1, 3	3 doses vs. 1		6.9 (5.0-9.4) †	4.8 (3.5-6.4) †
**Studies comparing GMTs by vaccine product**						
HPV-16	Cervarix & Gardasil (49)	3	Cervarix vs. Gardasil		5.8 (4.0-8.5)	5.9 (3.9-9.4)
	Cervarix & Gardasil (52)	3	Cervarix vs. Gardasil	4.3 ‡**		
HPV-18	Cervarix & Gardasil (49)	3	Cervarix vs. Gardasil		9.4 (6-14)	12.5 (8.0-19.4)
	Cervarix & Gardasil (52)	3	Cervarix vs. Gardasil	6.6 ‡**		
**Studies comparing GMTs across age groups**						
HPV-16	Gardasil (43)	3	10-15 vs. 16-23 y/o	1.8 (1.5-2.2)		
	Cervarix (53)	3	10-14 vs. 15-25 y/o	2.4 (1.6-3.5)		
	Cervarix (49)	3	18-26 vs. 27-35 y/o			2.0 (1.3-3.1)
	Gardasil (54)	3	9-12 vs. 13-15 y/o	1.5 ††		
HPV-18	Gardasil (43)	3	10-15 vs. 16-23 y/o	2.0 (1.7-2.4)		
	Cervarix (53)	3	10-14 vs. 15-25 y/o	1.9 (1.3-2.8)		
	Cervarix (49)	3	18-26 vs. 27-35 y/o			1.7 (1.1-2.6)
	Gardasil (54)	3	9-12 vs. 13-15 y/o	1.6 ††		
**Seropositivity at time of vaccination**						
HPV-16	Cervarix	1	SP vs SN		3.8	3.8
	Cervarix	3	SP vs SN		1.5	1.5
	Gardasil-9 (55)	1	SP vs SN		–	–
	Gardasil-9 (55)	3	SP vs SN		3.2	3.2
HPV-18	Cervarix	1	SP vs SN		2.0	2.0
	Cervarix	3	SP vs SN		1.7	1.7
	Gardasil-9 (55)	1	SP vs SN		–	–
	Gardasil-9 (55)	3	SP vs SN		5.0	5.0

GMT, Geometric mean titers; CI, Confidence interval; y/o, years old, SP seropositive; SN seronegative

† Costa Rica Vaccine Trial, unpublished results

‡ 1-to-6 months after the 3rd vaccine dose

†† 1-month after the 3rd vaccine dose

** Estimated from data provided in the paper, reported p-values for Cervarix vs. Gardasil = 0.0001

Expectation of Non-inferiority in Immunobridging Study

Based on the acceptance of immunogenicity as a surrogate for vaccine efficacy, our objective is to demonstrate that the immune response for 1-dose of Cervarix in girls 9-14 years old is non-inferior to the immune response for 3-doses of Gardasil in women 18-25 years old, a dosage/population combination with demonstrated efficacy. Although there are no direct

comparisons of the immune response in these two groups, we can extrapolate based on the following equation:

$$\frac{\text{GMT(1-D, C, 9-14yo)}}{\text{GMT(3-D, G, 18-25yo)}} = \frac{\text{GMT(1-D, C, 18-25yo)}}{\text{GMT(3-D, C, 18-25yo)}} \times \frac{\text{GMT(3-D, C, 18-25yo)}}{\text{GMT(3-D, G,18-25yo)}} \times \frac{\text{GMT(1-D, C,9-14yo)}}{\text{GMT(1-D, C,18-25yo)}}$$

In terms of notation, 1-D and 3-D abbreviate 1-dose and 3-dose administrations and C and G abbreviate Cervarix and Gardasil. On the right side, we have three ratios that were discussed in the section above (we focus on HPV16, as the predicted ratio is higher for HPV18).

At 24-months, the time of the proposed interim analysis, the 1-Dose to 3-Dose ratio should be greater than 1/9.1=0.11; the Cervarix to Gardasil ratio should be greater than 5.8; the 9-14-year-old to 18-25-year-old, with some assumptions should be greater than 1.8 (see numbers in **bold** above). Taking this evidence together, we predict

$$\frac{\text{GMT(1-D, C, 9-14yo)}}{\text{GMT(3-D, G, 18-25yo)}} > 0.11 \times 5.8 \times 1.8 = 1.14$$

At 36-months, the time of the proposed analysis, the 1-Dose to 3-Dose ratio should be greater than 1/6.6=0.15; the Cervarix to Gardasil ratio should be greater than 5.9; the 9-14-year-old to 18-25-year-old, with some assumptions should be greater than 2.0. Taking this evidence together, we predict

$$\frac{\text{GMT(1-D, C, 9-14yo)}}{\text{GMT(3-D, G, 18-25yo)}} > 0.15 \times 5.9 \times 2.0 = 1.77$$

Therefore, based on the available evidence, we believe that the plateau antibody GMT for 1-dose of Cervarix in girls 9-14 years old is non-inferior to the plateau antibody GMT for 3-doses of Gardasil in women 18-25 years old. Indeed, the above arithmetic suggests the 1-dose Cervarix regimen in adolescents may indeed elicit an even higher antibody response compared to the 3-dose regimen of Gardasil in women.

STUDY OBJECTIVES

The objective of this study is to demonstrate that the immune response for 1-dose of Cervarix in girls 9-14 years old is non-inferior to the immune response for 3-doses of Gardasil in women 18-25, a dosage/population combination with demonstrated efficacy. As immunobridging

studies are an accepted approach for approving new vaccine schedules and indications, we believe that this study, if it successfully demonstrates non-inferiority, will provide a basis for label change by regulatory bodies and inform recommendations by international agencies (i.e., WHO; immunobridging with readout at the time of plateau is addressed by the TRS962). This evidence can arrive 2-3 years prior to the completion of the ESCUDDO study (NCI 17-C-N108, CEC-ICIC-E093-2017).

We hope to provide regulators and public health policy makers with the necessary scientific information required to make evidence-based decisions related to large-scale implementation of sustainable, cost-effective HPV vaccine programs. Specifically, evidence of strong protection from a single-dose of Cervarix may enable nations to achieve extensive population coverage by vaccinating a much larger number of HPV-naïve individuals, which, in combination with herd immunity, will result in decreased cervical cancer incidence and mortality over time, in addition to reductions in other cancers. It would also support implementation policies that prioritize wider population coverage with one dose over initiating and completing a two-dose protocol region-by-region, which inevitably risks delays and gaps in coverage in some areas.

Primary Objective
To demonstrate that the immunogenicity (as determined by ELISA) of a single dose of Cervarix in 9-14 year old girls is non-inferior to the immunogenicity of three doses of Gardasil, administered at 0, 2, and 6 months, in 18-25 year old women 36 months after initial vaccination, with an interim analysis at 24 months after initial vaccination.

Either of following criteria will qualify as non-inferiority:

- The interim analysis will assess non-inferiority at 24 months. Non-inferiority will be demonstrated if the lower limits for the two-sided 99% confidence intervals of the GMT ratios (1-Dose Cervarix divided by 3-Doses of Gardasil at 24 months) for both HPV-16 and HPV-18 antibody levels exceed 0.67.
- The primary analysis will assess non-inferiority at 36 months. Non-inferiority will be demonstrated if the lower limits for the two-sided 96% confidence intervals of the GMT ratios (1-Dose Cervarix divided by 3-Doses of Gardasil at 36 months) for both HPV-16 and HPV-18 antibody levels exceed 0.67.

Endpoint definition for immunogenicity: We will measure HPV-specific serum antibodies using the VLP-based direct enzyme-linked immunoassay (ELISA) on serum collected at 24 and 36 months after initial vaccination. The 36-month timepoint is the primary objective; an interim analysis will be conducted at 24-month to determine if non-inferiority can be achieved earlier, with appropriate penalty to the α-level. In addition to the ELISA, other assays may be utilized to

better understand the response to vaccination, such as the measurement of neutralizing antibodies and avidity.

Cohort for assessment of immunogenicity: Participants who receive the appropriate number of doses during the predefined vaccination windows and do not have serologic evidence for the corresponding HPV type considered in the analysis (i.e., are HPV16 seronegative for HPV16 analysis, and are HPV18 seronegative for HPV18 analysis) at baseline will serve as the analytical cohort for evaluation of the study objective.

<u>Secondary Objectives</u>

1) To compare the distributions of both HPV-16 and HPV-18 antibodies levels, assessed at 24 and 36 months after initial vaccination, following a single dose of Cervarix in 9-14 year old girls with the corresponding distributions following three doses of Gardasil, administered at 0, 2, and 6 months, in 18-25 year old women.
2) To compare proportions with seroconversion based on both HPV-16 and HPV-18 antibody levels, assessed at 24 and 36 months after initial vaccination, following a single dose of Cervarix in 9-14 year old girls and following three doses of Gardasil, administered at 0, 2, and 6 months, in 18-25 year old women.
3) To compare GMTs, distributions, and seroconversion proportions for HPV-16 and HPV-18 antibodies, assessed at 24 and 36 months after initial vaccination, following a single dose of Cervarix in 11-14 year old girls and three doses of Gardasil, administered at 0, 2, and 6 months, in 18-25 year old women.
4) To compare GMTs, distributions, and seroconversion proportions for HPV-16 and HPV-18 antibodies, assessed at 1-month after vaccination and 1-year after vaccination, following a single dose of Cervarix in 9-10 year old girls and following a single dose of Cervarix in 11-14 year old girls.
5) To evaluate whether baseline variables (e.g., age, enrollment date, and district) are associated with GMTs, distributions, and seroconversion proportions for HPV-16 and HPV-18 antibodies, assessed at 24 and 36 months after initial vaccination.

We note that Secondary Objectives 3 and 4 are, in part, included in this study because of the introduction of Costa Rica's national HPV vaccination program in 2019, which aimed to vaccinate 10-year-old girls.

STUDY DESIGN AND METHODS OF TRIAL

<u>Overview</u>

The PRIMAVERA study is a non-randomized, open-label, Phase IIIb clinical trial comparing 1 dose of the bivalent HPV vaccine (Cervarix) in girls to 3 doses of the quadrivalent HPV vaccine

(Gardasil) in women. We will work within selected regions in Costa Rica and invite girls 9-14 years old (N=between 520 and 620; N=between 173 and 206 9-10 years and N=between 347 and 414 11-14 years) and women 18-25 years old (N=between 520 and 620) to visit dedicated study clinics. Randomization will not be utilized in this trial because age indicates vaccine product and number of doses. Therefore, vaccine product blinding is not necessary. Girls will be invited with their parents/legal guardians. At the first (baseline) study visit, the study will be described in detail. Written informed consent will be obtained from adult participants and parents/legal guardians of minor participants. Written assent will be obtained from girls 12-14 years old; girls younger than 12 will be provided with a study information document and their verbal agreement to participate will be recorded in the study management system.

The potential participant's medical history will be assessed by a clinician, including previous HPV vaccination. If needed for eligibility determination and at the clinician's discretion, the clinician can perform a physical examination to confirm good general health. If the potential participant is deemed potentially eligible, she will undergo blood collection. Participants 12 and older will be required to have a negative urine-based pregnancy test prior to vaccination, and sexually active participants will be instructed to use effective birth control methods (including abstinence) for 60 days after the last vaccination.

The initial vaccination will be administered at this baseline study visit; this will be either the first dose of Gardasil or the single dose of Cervarix. Participants will be monitored for adverse events at the clinic for 15 minutes following vaccination. Appropriate medical treatment will be readily available in the rare event that an anaphylactic reaction develops. A real-time contact service (such as a toll-free number or text messaging system) will be staffed for the duration of the trial so that participants may contact the trial's clinical team in case of study-related medical concerns or questions.

Among women scheduled to receive Gardasil, the second and third vaccine visit will occur two and six months following the initial baseline visit. Qualified staff and clinicians will conduct these study visits. After a negative urine-based pregnancy test, the visit will conclude with vaccination and monitoring for adverse events. All adverse events, serious and not serious, will be documented.

All participants will have blood collected at the 12, 24, and 36 month follow-up visits, either in a clinic or home setting (Table 2). For participants in the Cervarix group, blood also will be collected at a follow-up visit one month after vaccination. Each girl is expected to have five study visits, one for vaccination and four follow-up visits. Each woman is expected to have six study visits, three for vaccination and three for follow-up.

Table 2. Proposed study visits for the two groups.

	Visit type (months)						
Age	V00*	V01	V02*	V06*	V12	V24	V36
9-14 years	X	X			X	X	X
18-25 years	X		X	X	X	X	X

*Indicates vaccination visit

Throughout the duration of the trial, we will monitor plans for country-wide implementation of HPV vaccination, which will target fourth-grade girls (usually 10 year olds). At the time when the country begins vaccination, we will initiate procedures for ceasing enrollment of 9- and 10-year olds according to the SOP requested by the DSMB. Note, our recruitment plan for the younger cohort ensures that we will include 347 girls that will not be targeted by the national vaccination program.

Finally, if at 36-months the GMT for 1 dose at the group level is inferior to that of 3 doses, all 1-dose girls will be invited to receive the 2nd dose of Cervarix.

Selection of Study Vaccines

Both vaccines to be used in this study are licensed in the US and Costa Rica. As described, the bivalent HPV vaccine is produced by GSK (Cervarix®) and contains HPV 16 and 18 antigens, the quadrivalent vaccines is produced by Merck (Gardasil®) and additionally contain HPV 6 and 11 antigens. Besides the differences in HPV types included, the bivalent vaccine has a proprietary adjuvant called AS04, consisting of monophosphoryl lipid A (MPL) and aluminum hydroxide. MPL is a detoxified bacterial lipopolysaccharide which is a TLR-4 agonist involved in activation of innate and adaptive immune responses. The quadrivalent vaccine has aluminum hydrophosphate sulfate as adjuvant.

The quadrivalent HPV vaccine is now included in the Costa Rica national vaccine program as a two-dose administration for 10-year old girls, which aligns with the official recommendation of PAHO, which recommended a schedule of two doses for adolescents under the age of 15 years in 2013 (50,51). Beyond the PAHO recommendation, the Cervarix product details (Version number: GDS024/IPI019) state that the two-dose regimen has been approved in Costa Rica for girls younger than 15 ("9-14 years inclusive: Two doses of 0.5 ml each. The second dose given between 5 and 13 months after the first dose or three doses of 0.5 ml each at 0, 1 and 6 months”). The Gardasil product details (quadrivalent vaccine, MSD 70031548/00-1) state that two doses of 0.5 ml each can be given to girls between 9 to 13 years at 0 and 6 months or 0 and 12 months. Per this same document, for all others, three doses of 0.5 ml each should be given at 0, 2 and 6 months.

Selection of Gardasil (and not Gardasil9) as control vaccine product

Efficacy studies using the quadrivalent HPV vaccine were conducted on women; for HPV types 16 and 18, the nonavalent HPV vaccine was subsequently approved based on observed non-inferior immunogenicity among the same aged women. This new trial aims to directly immunobridge to a population with demonstrated efficacy. Bridging to the nonavalent HPV vaccine would, in essence, bridge to a bridged population. This indirect approach would not be as definitive as bridging directly to the efficacy population. Yet, the plateau HPV16 antibody levels for Gardasil9 are not different compared to Gardasil and are 20% higher for HPV18 (56). Thus, if procurement of Gardasil is not possible, we will use Gardasil9 as the control vaccine.

Selection of HPV 16 and HPV 18 antibody levels as outcome

Although Gardasil additionally protects against the non-carcinogenic HPV types 6 and 11, which cause 95% of genital warts, this proposal only aims to evaluate the carcinogenic HPV types 16 and 18. Approximately 50% of cervical cancer is caused by HPV16, an additional 20% by HPV18, and the remaining 30% by 10 other carcinogenic types collectively (7). While reducing the burden of genital warts is beneficial, our aim is to dramatically reduce cervical cancer incidence and mortality globally, hence the focus on the two most cancer-causing HPV types.

Target populations

Two groups will be investigated in the current proposal: 1) the efficacy population (women between the ages of 18 and 25, in which efficacy was previously demonstrated) and 2) the adolescent population (girls 9 to 14 years). The efficacy population was defined based on the trials documenting efficacy, which led to licensure. The FUTURE-II trial of GARDASIL established vaccine efficacy in 15-26 year-old females, the PATRICIA trial of Cervarix established efficacy in 15-25 year-old females, and the Costa-Rica Vaccine Trial of Cervarix established efficacy in 18-25-year-old women. Hence, we believe the oldest acceptable bridging population is 18-25 year olds.

The World Health Organization (WHO) states that the primary target group in most of the countries recommending the HPV vaccination is young adolescent girls. Specifically, the WHO recommends a multi-age cohort during the first year of programmatic HPV vaccine implementation from 9-14 years with 2 doses of any of the licensed HPV vaccines. Hence, our objective is to establish vaccine efficacy for girls in this age range.

Participant eligibility will not consider sexual history. Participants with evidence of prior HPV exposure, assessed retrospectively by HPV serology at baseline, will not be excluded from the study but will be excluded from the primary analysis.

Costa Rica as the study setting
This study will be conducted using the established infrastructure used in the ongoing ESCUDDO study.

This study will occur outside the catchment area for ESCUDDO. Therefore, we have identified twenty districts that combined, based on census data, have ~1600 girls and ~2300 women in the eligible age ranges for this study. These districts are in Guanacaste and we expect high rates of participation and follow-up.

Specimen collection
Blood is the only biospecimen that will be collected; it will be used for research purposes. Urine will be collected from participants 12 and older and tested for pregnancy at vaccination visits to assess eligibility for vaccination, but not stored.

Eligibility criteria
Within the selected PRIMAVERA catchment area (i.e., the 20 selected districts) all girls and women who are in the study age ranges will be invited to participate. Self-referral is permitted, as long as the potential participant is a resident of the catchment area.

Inclusion criteria
Potential participants will be deemed eligible if they are:

- Female;
- Aged between:
  - 9 and 14 years inclusive for Cervarix group;
  - 18 and 25 years inclusive for Gardasil group;
- Living in the study area without plans to move outside the country in the next six months;
- Able to communicate with study personnel;
- Able and willing to provide a blood sample;
- Willing to permit export of blood samples to the United States;
- Willing to participate in the study and:
  - If Cervarix group and less than 12 years old, receive study information and be supported in study participation by at least one of parent (or guardian), who is willing to sign the informed consent document;
  - If Cervarix group and 12 years old or older, sign the informed assent and be supported in study participation by at least one parent (or guardian), who is willing to sign the informed consent document;
  - If Gardasil group, sign the informed consent.
- In good health as determined by a medical history (physical exam will be conducted if necessary per the doctor’s criterion).

Exclusion criteria

Potential participants will be excluded from enrollment if:

- They have a diagnosis of an autoimmune, degenerative, or neurological disease without treatment or adequate control; a progressive or severe neurological disease; a genetic immunodeficiency; or any other serious chronic disease without treatment and / or adequate control that, according to the principal investigator or designee, for which vaccination is contraindicated (NOTE: Potential participants with these conditions can be included after consultation with the external medical advisor of the study or with an appropriate specialist);
- They are allergic to one of the vaccine components, including yeast (if Gardasil group);
- They have received immunoglobulins within 90 days preceding enrollment/vaccination visit;
- They are unwilling to provide a blood sample;
- Unwilling to permit export of blood samples to the United States;
- They have a positive urine pregnancy test result;
- They are pregnant;
- They are planning to become pregnant.
- The clinician determining eligibility in agreement with the principal investigator considers that there is a reason that precludes participation;
- They have been vaccinated against HPV;
- The participant or her parent/legal guardian, as applicable, does not have an identification document.

Deferral criteria at enrollment:

The enrollment visit will be deferred for either study arm if:

- They have an acute disease that precludes vaccination (though vaccines can be administered to potential participants with a minor illness such as diarrhea and mild upper respiratory infection);
- Are being evaluated by a clinician to rule out or confirm a diagnosis of a chronic disease;
- The blood sample is not able to be collected.

Contraindications to subsequent vaccination (Gardasil group)

Participants in the Gardasil Group will not receive additional vaccine doses if they:

- Present a significant hypersensitivity reaction following the first study vaccine administration;
- Are diagnosed with any medically-documented immunodeficiency, autoimmune, or degenerative conditions;
- Are diagnosed with any other newly acquired chronic condition which, in the opinion of the principal investigator, precludes further administration of the study vaccine;

- Present any SAE judged to be possibly, probably, or definitely related to study vaccine that may pose a risk to the participant should she receive another vaccine dose; or
- Present any other significant reactions including severe pain, severe swelling, severe limitation of motion, persistent high fever, severe headache or other systemic or local reactions, which in the opinion of the study doctor and in consultation with the investigator preclude further administration of the study vaccine.

Gardasil Group participants who do not receive the second or third vaccine dose will continue to be followed for the full length of study.

Concomitant Medications/Vaccinations
Concomitant medications administered during this study will be assessed in the context of AEs assessment only, as described in the adverse event reporting section.

<u>Outreach and Enrollment Visits for Both Cervarix and Gardasil Groups (0 months)</u>
We will use multiple methods to reach potential participants within the PRIMAVERA catchment area. The study will be publicized through attending community events, distributing outreach materials in commercial and residential areas, making announcements, and other means. Potentially eligible participants and parents/guardians of potentially eligible participants who are present during outreach activities will be informed about the study.

If a potentially eligible participant (and her parent/guardian, as applicable), is interested, a participant study ID (PID) will be assigned and an enrollment appointment will be scheduled for her to attend a study clinic.

During the enrollment visit at the clinic, the study will be explained in detail to the prospective participant and her parent/ guardian, as applicable. The informed consent and assent processes will be completed for those willing to participate. Consent for accessing the participant's medical records from any medical institutions will be administered using separate consent and assent forms. If preferred, due to participant or parent's/guardian's needs, a visit may be requested to complete the informed consent/assent processes at home, prior to the enrollment appointment.

After administration of the informed consent, the clinic team will proceed with eligibility determination. Among participants younger than 12 years old, the parent/guardian must be permitted to be present during all of the visit components. Among participants 12 years and older, the parent or guardian will be asked to leave the examination room while the pregnancy test is performed.

Participants 12 and older will be asked to provide a urine sample for a pregnancy test, a component of the eligibility determination.

To continue the eligibility assessment, a clinician will assess the participant's general health and rule out contraindications for receiving the vaccine, such as history of a chronic condition or allergies to any vaccine components. For girls 9-14 years old, if necessary, the clinician will consult with the participant's parent/legal guardian. If the medical doctor considers necessary, based on the medical assessment, a physical examination will be performed to confirm good general health. If the medical assessment suggests an undiagnosed medical condition, the doctor may perform a directed physical examination to confirm a diagnosis, rule out a condition, or refer the potential participant for further medical evaluation.

Finally, a blood sample will be collected. If the sample is not able to be drawn, the visit will be rescheduled. If the participant refuses the blood sample, she will be excluded from the study and will not receive the vaccine.

An eligibility checklist will be completed during the eligibility determination phase of the enrollment visit to document whether the participant meets all the study and vaccine eligibility criteria.

Once a participant meets the eligibility criteria and can be vaccinated, she will proceed with the vaccination procedures. After vaccination, participants will be observed for 15 minutes, per CDC guidelines, and as stated in the prescribing information for Cervarix and Gardasil. Appropriate medical treatment will be readily available in the rare event that an anaphylactic reaction develops. A real-time contact service (such as a toll-free number or text messaging system) will be staffed for the duration of the trial so that participants may contact the trial's clinical team in case of emergency.

Participants will be instructed to not become pregnant for about two months following vaccination and will be encouraged to use an effective birth control method if sexually active. This aligns with the labels for these vaccines, which state that the safety and effectiveness of the vaccines has not been established among pregnant women. Participants will be informed that while no risks have been observed for vaccination during pregnancy, the subject has not been well studied. For participants 12 years old and older, information about birth control methods will be provided upon participant request; however, we will not promote use of specific birth control methods.

<u>Gardasil Group Followup Vaccination Visits (2 and 6 months)</u>

Among women scheduled to receive Gardasil, the second and third vaccine visit will occur two and six months following the initial baseline visit. At these visits, vaccine eligibility will be assessed by the doctor or other qualified health professional, including assessment of any occurrence of serious adverse event reactions or newly chronic conditions diagnosed after the first vaccine.

As in the enrollment visit, a negative urine-based pregnancy test and report of no plans to get pregnant will also be required. Additionally, the health professional will complete a brief assessment to confirm that there are no vaccine contraindications and the participant is eligible to receive the second or third vaccine dose.

If, during eligibility determination at the two- or six-month visit, a woman has a positive pregnancy test, she will be deferred, and her visit discontinued. If a woman is found to be ineligible for the second or third vaccine for another reason (i.e., related to vaccination only), she will be excluded from vaccination and will proceed with the other two- or six-month visit data collection procedures.

If a woman meets the two- and six-month visit criteria to be vaccinated, she will proceed with vaccination. After vaccination, observation for 15 minutes will be completed and monitoring for adverse events performed as in the enrollment visit.

Active Follow-up of Both Groups (Months 1, 12, 24, and 36)
All participants will have follow-up visits every 12 months, and participants in the Cervarix group will have an additional visit one month after the vaccination. These visits will be conducted either in the home or a clinic setting. At each follow-up visit, the doctor or other qualified health professional will assess eligibility to complete the visit and collect updated HPV vaccination data. The 12-month visit for Gardasil group participants will include assessment of any occurrence of serious adverse event reactions or newly chronic conditions diagnosed after the final vaccination. Finally, blood will be collected from all willing participants. These visits can be completed at home.

Home Visits During Follow-up
All follow-up visits may be conducted at either the clinic or at home (at the convenience of the participant and/or her parent/guardian, as applicable), if an appropriate environment for performing blood collection is provided. The clinic team member who schedules the home visit will determine, based on an SOP created by the Investigators, whether the home setting is appropriate.

Procedures and data collection

Medical history
At the enrollment visit, we will conduct a complete medical history (and physical examination if considered necessary by the clinician evaluating the eligibility) to determine the participants' overall health status and eligibility for the study.

Among younger participants, the medical history will initially be completed with the participant's parent or guardian present to facilitate collection of information about any aspect

of her medical history with which the participant may not be familiar. After the first assessment of the medical history, the parent/guardian is asked to wait in the outer clinic area only if the participant is 12 years old or older. Then, the doctor will review the results of the pregnancy test.

Along with determining whether a physical examination is necessary, the clinician will need to decide whether the parent or guardian should be present for the examination (if the participant is 12 years old or older). Participants under the age of 12 may remain with their parent/legal guardian during the entire visit. In any case, the nurse will always be present during physical examination.

Should any condition be detected or suspected by the study clinician, we will inform the participant (and her parents if a minor and deemed necessary by the attending medical doctor) of the possible/diagnosed condition and refer the participant to her doctor. Specific SOPs/instructive and forms to conduct and document the enrollment medical history and general physical exam if performed will be used.

Assessment of HPV vaccination outside the research study
During the enrollment visit and the follow-up visits, participants will be queried about additional HPV vaccinations received outside the research study. To bolster and confirm self-reporting, antibodies against HPV 6 and 11, the additional types included in the quadrivalent HPV vaccine that is provided as part of the Costa Rica National Vaccine Program, will be measured on a subset of participants as a biomarker to assess external HPV vaccination.

Urine Samples (Vaccination Visits only)
Along with the medical history, and before each vaccination, participants 12 and older will be asked to collect a urine specimen for a urine pregnancy test. Among participants 12 and older, only those with a negative pregnancy test will be enrolled.

The participant will be directed to collect her urine in the bathroom with the doctor and nurse aide available to help her if she chooses. After the urine pregnancy test is completed, any residual urine sample will be discarded.

Blood collection
Blood specimens will be collected from all participants at every enrollment and follow-up visit. The purpose of these blood collections will be to monitor immune response to vaccination, to address study objectives. Less than 10ml of blood will be collected from all participants at each visit.

In the clinic or home (in the case of in-home follow-up visit), during transport, and prior to processing, the samples will be stored according to standard operating procedures (SOPs) in a

cooler designed to safely and securely store and transport biological material according to local and international regulations. The blood will be stored at a temperature between 2˚C and 10˚C. Established SOPs provide instruction regarding the preparation and packing of the coolers, and proper operation and monitoring of the thermometers, and the actions needed to assure the required temperature for both storage and transportation.

Vaccine Procedures
Participants will be vaccinated intramuscularly in the deltoid muscle (upper arm), typically of the non-dominant arm, and will receive doses at enrollment for the Cervarix group, and, for the Gardasil Group, at enrollment and 2 and 6 months.

All study vaccines will be stored at ~4°C per manufacturer instructions. Established SOPs will be followed to ensure proper chain of custody covering both custody of the pharmaceutical product and cold chain compliance.

To assure adequate vaccine storage and custody, redundant power and refrigeration systems will be in place, in addition to temperature and access monitoring/recording systems. Vaccines exposed to damage or lack of custody will not be used in the trial and the event will be communicated to the Investigators.

Blood preparation, storage and shipment
We have experience collecting blood in Costa Rica from thousands of women over the last two decades as part of previous studies (57). Blood will be stored in cold boxes until transport to the local repository for preparation, freezing, and interim storage. Blood will be processed according to specified time requirements. For all blood samples, at enrollment and during follow-up, we will separate and store serum aliquots.

Following processing, serum will be prepared and stored temporarily at the local repository, frozen at ultra-low or cryogenic temperature, until shipment to the NCI biorepository for long-term storage. All serum aliquots will be shipped to the NCI repository. Further distribution will be done as needed, to collaborating laboratories for testing. A shipping and customs agent will be responsible for these shipments. MTAs between NCI and Costa Rican investigators complying with US and CR regulations will be in place before any shipment is conducted.

Expected Adverse Events and Adverse Event Monitoring and Reporting
There is considerable accumulated data on the safety of these FDA- and EMA-approved vaccines. Given the design of the trial, the study will lack reference rates of occurrence of specific events among unvaccinated women. Nevertheless, we will monitor all trial participants for adverse events (AEs) at the clinic for at least 15 minutes after vaccination with appropriate medical treatment readily available in case of a rare anaphylactic reaction. Adverse events (AEs)

occurring during this period will be documented and followed to resolution. In case participants present non-serious expected adverse events like site reactions (mild pain), dizziness, or headache they will receive recommendations and Paracetamol will be available.

The consent and assent forms include a discussion of possible adverse events and will advise participants and their parents/guardians about what will happen if they experience an adverse event during the clinic visit. The consent and assent forms will also give guidance on how for participants to report adverse reactions that occur after the participant leaves the visit. The consent forms explain the study’s insurance coverage and that, per Costa Rican Human Research Law, the study may pay for the care of conditions determined to be related to the study vaccines or procedures.

In addition, following each vaccination, participants will be told that they should report to study staff any serious problems they have in the month after each vaccine administration that require medical attention, whether they think the problem is related to vaccination or not.

Finally, participants will be actively queried at the study visit that follows each vaccine administration regarding SAEs that occurred after vaccination. Additionally, Cervarix group participants will be contacted at 6 months, either by phone or personally to inquire about vaccine reactions and to maintain contact to promote compliance with follow-up visits. In either case, the clinician will probe for adverse events by asking the participant whether she has been diagnosed with an illness, disabled for more than one day, or hospitalized since her last visit. These questions are included in the eligibility screener at the second and third vaccination visit, and the 12-month visit for the Gardasil Group, and in the six-month contact script for Cervarix group.

For the full duration of the trial, AEs spontaneously reported by study participants will be documented. All deaths will be documented irrespective of timing between the occurrence of the event and vaccination.

All the adverse events, serious and not serious, reported by participants as part of active monitoring during the visit after vaccination completion (i.e., the 6-month phone contact for Cervarix group participants, and the 2-, 6- and -12-month visits for Gardasil group participants), or as part of passive monitoring throughout the duration of the study (i.e., reported spontaneously by the participant) will be documented. All events that are not resolved at the time of reporting will be followed up to document their resolution; if the SAE results in a chronic condition, it will be followed until medical management of the condition is documented. Should the event result in hospitalization, or otherwise require hospital record review according to the criteria of the Principal Investigator or designee, trained staff will review the medical records at the hospital, if necessary, and will extract relevant information to

clarify the evolution, diagnosis, treatment and resolution of the event. In a separate informed consent/assent form, participants and their parents/guardians, as applicable, will be asked to provide authorization for review of hospital charts if necessary.

Non-serious AEs directly related to study procedures other than the vaccine (e.g., blood collection) also will be documented and followed to resolution.

The Costa Rica Principal Investigator will report SAEs to the responsible regulatory authorities in Costa Rica, and to the NCI Principal Investigator. The reporting of AEs to regulatory entities in the United States will be the responsibility of the NCI Principal Investigator. Procedures for reporting AEs in the United States will be determined according to the NCI's Ethical Review Panel (ERP) guidance.

The Costa Rica Principal Investigator will report AEs to the Costa Rica IRB as stipulated in the regulation of the Human Subject Research Regulatory Act (Law 9234). Additionally, the Costa Rica Principal Investigator will report all AEs related to vaccination to the Pharmacovigilance office of the Costa Rica Minister of Health.

The DSMB will also monitor AEs for safety. The DSMB will review AEs according to their reporting and meeting schedule, as specified in the DSMB Charter.

Protocol Deviation Monitoring and Reporting
Serious and non-serious protocol deviations will also be reported to the Costa Rica and NCI IRBs. The Costa Rica Principal investigator will report protocol deviations to the local IRB in interim reports in compliance with IRB and national regulations.

It will be the NCI Principal Investigator's responsibility to report to entities in the U.S. Procedures for reporting of serious and non-serious PDs will be determined according to the ERP guidance.

Internal and external trial monitoring will be conducted.

Data collection and electronic records
Electronic case report forms (eCRFs) will be used in lieu of paper case report forms. Data will be collected using devices with custom-developed applications and programs. Real time edits and validation checks will be implemented into the eCRF system. All data that are considered personally identifiable will be stored encrypted, if stored locally on a cellular device. All data that are sent from any electronic device to the main servers will be sent encrypted. Physical security measures will be placed around all devices that are to be used to collect data electronically at the clinic. The main servers will be stored in a restricted access location and will only be able to be accessed by systems administrators and other authorized IT personnel.

A strict and rigorous permission-based editing system will be implemented to allow only certain personnel with specific permissions to modify data once that data has been committed to the main database. A full audit trail will be kept on all data modifications, which will include the user making the change, date and time, old value, new value and reason for the change. The modifications will be viewable to monitoring personnel in order to ensure full traceability.

Serology

HPV binding is measured by ELISA. The quantity of the HPV16 and 18 IgG antibody response measured by VLP ELISA will be utilized to address immunogenicity endpoints.

To further define the primary analytic cohort, HPV6 and HPV11 antibodies will be measured by VLP ELISA in a subset of participants to confirm, beyond self-report, that the according to protocol analytic cohort includes only girls who did not receive external HPV vaccination through the national program, which uses quadrivalent vaccine (HPV 6, 11, 16, 18). This will assure that girls in the single-dose HPV vaccination arm did not get additional doses.

HPV neutralization is measured by SEAP-NA. Antibody levels measured by ELISA will be compared to neutralizing antibody levels measured by the pseudovirion-based neutralization assay, SEAP-NA. This nested study will select 36-month serum samples from a random set of 100 18-25 year old women who are HPV 16/18 seronegative at baseline and a random set of 100 9-14 year old girls who are HPV 16/18 seronegative at baseline, and then evaluate the HPV-16 and HPV-18 antibody levels in each of those 200 samples using both ELISA and SEAP-NA. For each HPV type, we will perform a Deming regression comparing log(ELISA) levels with log(SEAP) levels, where log(ELISA) is the dependent variable and log(SEAP) is the independent variable. We will consider ELISA as an acceptable assay for the primary endpoint if the 95% confidence intervals of the regression slope excludes $1-\Delta$ and $1+\Delta$ for both HPV types, with $\Delta = 0.3$. If the true slope is $b=0.9$, the variance of the measurement error for both log(ELISA) and log(SEAP) is $\sigma^2 = 0.4$, and the total variance of log(SEAP) = 1.9, then the power to achieve this serology objective is 0.94. Preliminary data suggests that $0.9 < b < 1$ and $0.10 < \sigma^2 < 0.45$. The power for other combinations of sample sizes, slopes, and variances are provided in Table 3. Note the power is significantly reduced if we consider each group of females separately. Furthermore, we will estimate the intra-assay measurement error by measuring 30 samples, 15 from 9-14 year old girls and 15 from 18-25 year old women, twice on each of the assays. **Therefore, for the purposes of the serology study, the total number of samples run on each assay will be 230**. If we do not achieve the serology objective, we will evaluate the data with partners at regulatory agencies to determine whether ELISA can be still be considered as an acceptable assay for measuring the primary endpoint.

Table 3: Power calculations. The table lists the power to demonstrate that the 95% confidence interval for the slope describing the relationship between ELISA and SEAP-NA measures, on the log scales, excludes 0.7 and 1.3 for the specified sample sizes (N = 50, 100, 150, or 200), slopes (b = 0.9, 1.0), and variances (σ^2 = 0.25, 0.45).

	b = 0.9			
	N = 50	N = 100	N = 150	N = 200
σ^2 = 0.25	0.54	0.92	>0.99	>0.99
σ^2 = 0.45	0.12	0.48	0.73	0.88
	b = 1			
	N = 50	N = 100	N = 150	N = 200
σ^2 = 0.25	0.85	>0.99	>0.99	>0.99
σ^2 = 0.45	0.17	0.79	0.97	>0.99

Efforts to maximize participation and retention

Efforts aimed at promoting participation during enrollment, and minimizing withdrawal and losses during follow-up, include:

- Intensive training on communication skills for staff who will be conducting outreach activities to potential participants and their parents/guardians, as applicable.
- Multiple efforts at different times will be made to contact potential participants and their parents/legal guardians.
- Flexible clinic hours to accommodate scheduling needs of participants and parents/guardians.
- The option to complete the informed consent/assent procedures and/or follow-up visits (i.e., the 24-month and 36-month visits) in the home in order to accommodate participant's and parent's/guardian's scheduling needs (provided that a suitable space is available, according to established SOPs).
- Free phone line for potential participants to call and request or change an appointment.
- Reminder phone calls and text messages about appointments.
- Reimbursement for transportation costs, if needed, and, if requested, transportation to the clinic in a study vehicle.
- Exclusion of participants who report (or whose parents report) they have definite plans to move out of the country within six months of enrollment.

Efforts described above should minimize withdrawals from the study prior to the completion of 36 months of follow-up. In the event that a girl or woman withdraws from the study, the reason for withdrawal will be documented.

Discontinuation of vaccination (i.e., withdrawal from vaccination) will not constitute grounds for withdrawal from study follow-up. The occurrence of an AE will also not necessarily constitute grounds for withdrawal.

Approval of the exiting of participants from the study

The study is proposed to last three years after initial vaccination for each participant. Once the last study visit is completed, the participant chart will be reviewed to ensure that all serious adverse events are resolved or, if chronic conditions are present, that they are under medical care. The participant will then be closed out from the study. As mentioned above, if the GMT for 1 dose at the group level is inferior to that of 3 doses based on the 36-month analysis, all 1-dose girls will be invited to receive the 2nd dose of an HPV vaccine. Previous trials documented that delayed administration of a booster dose did not diminish the subsequent increase in antibody levels (58).

ANALYSIS PLAN

Primary Analytical Cohort

To evaluate the non-inferiority objective in the trial, an According to Protocol (ATP) analytical cohort will be defined. The ATP cohort for a specified HPV type (i.e. HPV 16 or HPV 18) and timing (i.e. 24-month or 36-month) are the participants who satisfy the following set of requirements:

- Received the correct number of doses within the predefined ATP vaccination windows. Denoting T_1 as the time of the first vaccination, the analytical window for the second vaccination is between T_1+30 days and T_1+90 days. Denoting T_2 as the time of the second vaccination, the analytical window for the third vaccination is between T_2+90 days and T_1+360 days.
- Not seropositive at baseline for the specified HPV type.
- Blood collected within the predefined ATP follow-up windows.
- No HPV vaccination outside of the study prior to blood collection. The participant did not report receiving an HPV vaccine and, among those tested, did not have HPV6 and HPV11 vaccine-level antibodies.

The cutoff for HPV16 seropositivity is greater than 1.41 international units (IU)/mL and for HPV18 is greater than 1.05 IU/mL. All serological measurements will be presented in international units Restricting to HPV16 or 18 seronegatives using this cutoff also aligns the Gardasil cohort in this study with the efficacy cohort used in the licensure of Gardasil (Future I and II) (12).

Immunogenicity Endpoint

The four endpoints for the primary objective are HPV-16 and HPV-18 specific antibody results obtained from blood specimens (serum) collected at the month 24 (interim) and 36 (primary) month visit (i.e. 4 endpoints = 2 HPV types x 2 time-points). All antibody levels are measured by HPV type-specific ELISA. Two additional endpoints, HPV-16 and HPV-18 antibody levels measured from serum collected at the 1-month visit and the 1-year visit, will also be included to achieve Secondary Objective 4.

Analysis

We will estimate the GMT ratios for four primary endpoints: HPV-16 and HPV-18 at 24 and 36 months.

- We define $R^H(t) = GMT_1^H(t)/GMT_3^H(t)$ to be the GMT ratio for HPV-H (i.e. HPV-16 or HPV 18) at time point t (i.e. 24 or 36 months) comparing type-specific antibody levels in 9-14 year old girls receiving 1 dose of Cervarix to levels in 18-25 year old women receiving 3 doses of Gardasil.

- We will estimate $R^H(t)$ by fitting a generalized estimating equations (GEE) model where the dependent variable is the log-antibody level and the independent variable is study-group. Denoting the estimated coefficient for study-group by $\hat{\beta}^H(t)$, we define our estimate of the desired ratio by $\hat{R}^H(t)$ = $\exp(\hat{\beta}^H(t))$. In the model, we will group girls into clusters of full siblings and use an exchangeable correlation matrix.

- We will estimate the two-sided 99% confidence interval of the GMT ratios for HPV-16 and HPV-18 at 24 months and the two-sided 96% confidence intervals of the GMT ratios for HPV-16 and HPV-18 at 36 months.

- Denoting the corresponding robust standard error of $\hat{\beta}^H(t)$ by $\hat{\sigma}^H(t)$, we define the two-sided (1-α)% confidence interval (CI), $(\hat{R}^H_{\alpha/2}(t), \hat{R}^H_{1-\alpha/2}(t))$, by $\exp(\hat{\beta}^H(t) \pm z_{1-\alpha/2}\hat{\sigma}^H(t))$, where $z_{1-\alpha/2}$ is the 1-α/2 quantile of a normal distribution.

We will declare non-inferiority if $\min\left(\hat{R}^{16}_{0.005}(24), \hat{R}^{18}_{0.005}(24)\right) > 0.67$ or, upon failure of that criterion, $\min\left(\hat{R}^{16}_{0.02}(36), \hat{R}^{18}_{0.02}(36)\right) > 0.67$. Note, under the null hypothesis that both $\min(R^{16}(24), R^{18}(24)) < 0.67$ and $\min(R^{16}(36), R^{18}(36)) < 0.67$, the probability of declaring non-inferiority or the Type I error rate is less than 0.025 for HPV-16 and HPV-18 endpoints, respectively.

Because the national vaccination program has been initiated, there is the potential that some girls enrolled at ages 9-10 years may have received an additional vaccination dose but have failed to report it. Therefore, we plan to conduct the following secondary/sensitivity analysis. We will compare GMTs, distributions, and seroconversion rates for HPV-16 and HPV-18 antibodies, assessed at 24 and 36 months after initial vaccination, between 11-14 year old girls receiving 1 dose of Cervarix and 18-25 year old women receiving 3 doses of Gardasil. We will also calculate the GMT ratios, comparing girls 9-10 years old at vaccination with girls 11-14 years old at vaccination, and their 95% confidence intervals for HPV-16 and HPV-18 antibody levels at 1 month and 1-year post-vaccination. Additionally, we will test for HPV6/HPV11 antibodies in a subset of participants who are at-risk for external HPV vaccination or show serologic evidence of additional HPV vaccine doses.

Power and Sample Size Considerations

We calculate the power to show non-inferiority at 24 months and the power to show non-inferiority at 36 months given that we enroll 520 women 18-25 year old and 347 girls 9-14 year old. Specifically, we assume that $\hat{\sigma} = 1.38$, 20% of all 18-25 year old women do not receive all 3 doses, 100% of 9-10 year old girls (i.e. worst case scenario) receive a vaccination outside the study, 20% of all participants are lost-to-follow up, 10% of the 18-25 year old women are seropositive at baseline, 5% of the 11-14 year old girls are seropositive at baseline, the common true ratio, R, for HPV-16 and HPV-18 is 1, and the non-inferiority threshold (t) is 0.67, in accordance with the recommendation of the WHO Expert Committee on Biological Standardization(60). Based on these assumptions, we will have 65% power to demonstrate non-inferiority at 24 months and 84% power to demonstrate non-inferiority at 36 months. Power calculations for other values of R and t are listed in table 4. Note the power reported for 36 months is $\Pr\left(\min\left(\hat{R}^{16}_{0.02}(36), \hat{R}^{18}_{0.02}(36)\right) > t\right)$, a lower bound for the probability of rejecting non-inferiority at either the 24- or 36- month analysis. In the more optimistic scenario, where 9-10 year old participants do not receive vaccinations outside the study, our power increases to 78% at 24 months and 91% at 36 months.

For the potential secondary analysis, we would calculate the GMT ratios, comparing girls 9-10 years old at vaccination with girls 11-14 years old at vaccination, and their 95% confidence intervals for HPV-16 and HPV-18 antibody levels at 1 month and 1 year post-vaccination. Assuming that enrollment includes 173 girls 9-10 years old and 347 girls 11-14 years old, 5% of all girls are seropositive at baseline, 10% of all girls do not return for the 1 month visit, R = 1, and $\hat{\sigma} = 1.38$, the expected length of the 95% confidence interval for $\hat{R}^{H}(1)$ is 0.55 (i.e. if $\hat{R}^{H}(1) = 1$, the expected 95% confidence interval would be 0.76 – 1.31).

Table 4: Power calculations. The table lists the power to demonstrate non-inferiority at the 24- and 36-month analyses for a non-inferiority threshold of 0.67 and three values of the common true ratio (R=0.8, 1.0, 1.2), with other parameters set at the values described in the text.

	Threshold = 0.67	
	Power (24 months)	Power (36 months)
R = 0.8	0.02	0.09
R = 1.0	0.65	0.84
R = 1.2	>0.99	>0.99

HUMAN SUBJECTS PROTECTIONS

This trial will be conducted in compliance with regulations for human subject research in effect in Costa Rica (Costa Rican Human Research Law 9234) and the United States (The Belmont Report & Title 45 Code of Federal Regulations, Part 46, Protection of Human Subjects, and Declaration of Helsinki). The principles of respect for persons, beneficence, and justice will be paramount in planning our trial with all the necessary considerations related to inclusion of minors.

In additional to specialized training associated with their roles, all study staff involved in study interactions with participants, their confidential data or samples (e.g., outreach workers, clinic staff, relevant systems staff, laboratory staff, sub-investigators and principal investigators), will complete GCP training in accordance with the requirements of the Costa Rican Ministry of Health, which authorizes them to conduct human research. They will sign a confidentiality agreement as part of their contract.

Institutional Approvals

The initial protocol was approved by the Senior Advisory Group (SAG) Committee of the Division of Cancer Epidemiology and Genetics on September 17, 2018. All comments and suggestions which came out of the SAG review were incorporated. In addition to the NCI IRB, we will obtain IRB approval from a Costa Rican Ministry of Health-approved institutional review board designated by Fundación INCIENSA under an appropriate FWA.

Justification for exclusions and dose schedule

Most vaccine programs seek to immunize 9- to 12-year-old girls. However, since individual level benefit has been documented at older ages, catch up vaccination through the age of 26 years is also recommended in many countries. Our study will enroll women up to age 25.

Although HPV vaccination has been shown to be equally effective in boys, this study will focus only on females because, in LMIC, women bear the overwhelming burden of HPV-driven disease. To our knowledge, none of these countries is considering gender-neutral vaccination strategies. Thus, it is important to maximize the amount of information on the target population.

Only girls and women with a negative pregnancy test will be enrolled as study participants. If a potential participant has an equivocal or positive pregnancy test, she will be excluded from enrollment and vaccination according to the vaccine use and recommendations approved by the Ministry of Health. This is according to the labels for these vaccines, which state that the safety and effectiveness of the vaccines has not been established among pregnant women.

Safeguards for vulnerable populations
For women who are 18 or older, only a signed consent from the participant is required. For participants 12-14 years old, permission from one of the parents or legal guardian will be required, along with a signed assent from the girl. For girls younger than 12 years old, permission from one of the parents or legal guardian will be required, along with the girl's verbal agreement to participate. This is in accordance with the Costa Rica law and the NCI IRB policy for consenting minors. A girl's failure to give assent is binding, and girls who are incapable of providing assent will be excluded. The permission will be documented by obtaining a signed consent form from a parent or legal guardian according to regulation, along with a signed assent form from the girl.

Different forms will be used for younger participants, one for ages 9-11 and one for ages 12-14. The length and complexity of the language in these forms has been reduced and simplified to aid comprehension but includes all the information required by law. In addition to the different language and content used in these forms, the way that willingness to participate is provided will differ. This is according to Article 17 of Costa Rican Regulatory Law of Biomedicine 9234, which specifies that signed informed assent is required starting at age 12. Girls 12-14 will be asked to provide written assent. Girls 9-11 will be administered a study information document and their verbal agreement to participate will be recorded in the study management system.

The result of the pregnancy test will be provided to the participant only, in private. Participants age 12-14 years old who have a positive pregnancy test result will be notified as follows:

- The clinician will discuss this with the participant, and in discussion with the participant, the clinician will select one of the following options:
    - Have the doctor inform her parents,
    - Inform her parents by herself, or
    - Not inform her parents at all.

- In all cases, the doctor will make sure the participant understands that PANI will be informed, that PANI will follow-up with her, and that ACIB will follow-up with her and/or PANI to ensure she receives PANI follow-up.

These procedures will be followed in order to avoid exposing participants to risk due to disclosure of pregnancy test results to their parents in cases where this represents a risk of aggression and in order to protect the participant's rights to knowing her information and to preserve confidentiality. This policy is consistent with Costa Rican regulations (Código de la Niñez y la Adolescencia - Law 7184, Código Procesal Penal - Law 7594, and Ley General de Protección a la Madre Adolescente - Law 7735).

The topic of safety during pregnancy has been evaluated using CVT data (61, 62). Results from these analyses have been reassuring with respect to the safety of vaccination during pregnancy. Although we will conduct urine pregnancy tests in participants 12 and older to rule out pregnancy prior to vaccination, in the event a participant is vaccinated while pregnant (i.e. due to a false negative pregnancy test) or becomes pregnant after the first dose, the second and third doses will be deferred until after pregnancy resolution (depending on whether the applicable study visit is still open). No special follow up is required and participants who become pregnant will continue in the study and will be followed for the full 3 years.

We note, however, that the expected number of vaccine exposures in pregnant women will be small and no internal control group exists in our study, so the information obtained from this study is expected to provide limited new information above and beyond what has already been published regarding adverse events associated with immunization during pregnancy.

All participants with positive pregnancy test results at the enrollment or at one of the other vaccine visits, as applicable, regardless of their age, will be informed about the need for pre-natal care and will be referred to the appropriate health care service for prenatal care and to the PANI for social follow-up in the case of the Cervarix group, which includes ensuring access to pre-natal care due to the high-risk nature of pregnancy during adolescence. We will support the participant (i.e., providing information, education, appointments coordination, etc.) in obtaining prenatal care at the appropriate health care service. Follow-up contact will be conducted to determine if the participant is attending prenatal care and to emphasize its importance. Additionally, if the doctor believes the participant needs immediate support, the doctor will contact by phone a study psychologist, who will guide the clinician on how to proceed, including immediate referral to appropriate institutions that provide support.

The Costa Rican government implemented school-based HPV vaccination with two doses of the quadrivalent vaccine in fourth grade girls (typically age 10) in 2019. Consequently, a subset of

study participants may receive, in addition to the one dose of Cervarix as part of the trial, one or two additional doses of Gardasil outside the study. Studies on mixed-dosing schedules of the HPV vaccines have confirmed its safety and immunogenicity (58, 63-65). In particular, researchers in Quebec conducted a trial evaluating the immunogenicity and safety of a mixed vaccination schedule randomizing girls into the following three arms: 1) one dose of the bivalent HPV vaccine followed by one dose of the nonavalent HPV vaccine, 2) one dose of the nonavalent HPV vaccine followed by one dose of bivalent HPV vaccine, and 3) two doses of the nonavalent HPV vaccine (64). From this work, mixed HPV vaccination schedules were documented to be sufficiently immunogenic (all participants were seropositive) and have an acceptable safety profile. The safety of a mixed dosing schedule is further supported by the recommendations of the CDC Advisory Committee on Immunization Practices, which states: “If vaccination providers do not know or do not have available the HPV vaccine product previously administered, or are in settings transitioning to 9vHPV, any available HPV vaccine product may be used to continue or complete the series for females for protection against HPV 16 and 18” (66).

The study team is sensitive to the issues associated with the vulnerable younger population of this study, in terms of parental consent and participant assent, and procedures that may cause discomfort. Together with the DSMB, we will conduct periodic reassessments of the protocol with regard to these considerations.

Consent process and documentation

The study will conduct community outreach in commercial and residential areas, including door-to-door contacts, to identify households with potential participants, and approach them. If the participant and parent/guardian, as applicable, are present and available to discuss the study, the objectives of the study will be explained to them. When possible, potentially eligible participants will be scheduled for a clinic appointment at a time that is convenient for the participant and her parent/guardian, as applicable, and outreach materials will be left with them to review and discuss.

In order to accommodate the participant or parent’s/guardian’s scheduling needs, members of the study staff who are trained to administer the informed consent forms will be available to complete the informed consent and assent administration procedures at the home. In such cases, even if a younger potential participant is unable to ask questions privately in the home, she will have the opportunity to ask questions, and withdraw or modify her assent status when she comes to the clinic to complete the enrollment visit.

Formal administration of the consent forms will typically take place at the clinic appointment. A trained consent/assent form administrator will welcome the participant and parent/guardian (if

applicable), seat them, and invite them to view a video on a tablet that provides a visual presentation of the assent and consent form contents; there will be a version for girls, a version for parents/guardians, and a version for adult participants. The team member administering consent will then present the consent, assent, and information forms, as applicable, and assist the potential participant and parent/guardian, as applicable, to read and review the content. The consent administrator will address any questions the participant and her parent or guardian may have and then assist them to complete the informed consent/assent and signature procedures. A trained consent/assent form administrator will be present at all times at the study clinic to address questions and concerns and keep study subjects informed about the study procedures and progress. The study clinician will be available to answer more medical questions. As described, we will use different forms according to the age of Cervarix group participants, as well as a consent form for parental consent of Cervarix group participants, who are all younger than 18; girls who become 12 during the follow-up must sign an informed assent. The Gardasil group will all be adults and thus require only a consent form for adult participants.

In accordance with Costa Rican research regulations, a witness signature will be collected. This witness will not be a member of the study staff and will not be given a copy of the assent/consent form, although a copy will be given to the girl and parent/guardian. The format of the witness signature implemented on the consent/assent forms will be as required by these regulations (Article 10 of Law 9234). In practice, the witness is usually a family member/parent of another participant, or a neighbor, not related to the research team, and who will have not access to the confidential information of the participant (Regulation of Law 9234: - Article 8, part a. “In all biomedical research in which human beings participate, except for those indicated in articles 7 and 12 of Law No. 9234, the researcher must obtain informed consent, individual, voluntary, express, specific, written and signed or with the fingerprint of the participant or his legal representative on all sheets, and an impartial witness on the final sheet. In the case of the legal representative: A copy of documentation should be included where the right to legal representation is registered.”; Article 8, part f. “The process of signing informed consent must be made in the presence of an impartial witness, who may be a family member, selected by the participant or his/her legal representative, without contact with the research team, researchers, Sponsors, OACs and ICOs. The witness must be a person with sufficient capacity to understand the scope of his performance, will not have access to the confidential information of the participant, nor be present when it is analyzed.”)

The consent forms will include separate yes/no fields for indicating consent or assent for long term storage of samples and data sharing, as well as contact for future studies. Consent for accessing the participant’s medical records from any medical institutions will be administered using separate consent and assent forms.

Subject privacy and confidentiality of data, including plans for sharing, future use, and disposition of study materials/biologic specimens

Study procedures will be conducted in an enclosed private environment. All study staff will be trained in ethical conduct of research. We propose to retain the data and specimens from the study for a period of up to 50 years. However, study subjects have the right to withdraw at any time by informing a member of the outreach or clinic staff, calling the central study number, or contacting the local PI to request consent withdrawal. Upon receiving such a request, study staff will document the consent withdrawal, including the source of the request (e.g., participant, parent/guardian, or PI/Designee); the specific components of consent withdrawal (e.g., Biobanking, Medical Chart Review, and Contact for Future Studies); and the reason for withdrawal, if known. If the participant wants to withdraw from the study this will be documented as a study discontinuation. All consent withdrawals and study discontinuations are reviewed by the PI or a designee and, if considered necessary, an additional contact will be attempted to provide missing information or clarify issues before approving the withdrawal. As applicable, the requestor's data will be excluded from the study and/or biological samples be discarded for future analyses.

Only informed assent and consent forms will be kept in hard copy in locked cabinets, with access limited to study personnel. Electronic data will be stored in a password protected centralized database, and access to these data will be granted to only authorized study staff and authorized IT personnel. Whenever these data are transferred out of the centralized database for analysis purposes or any other purpose, all personally identifying information that is not necessary for analysis will be removed, and transfer of the data will be done using a web-based secure file transfer service.

Information provided by study participants will never be used to directly identify them by NCI or outside investigators. Additionally, each study participant will be assigned a unique study number and a link will be maintained with personal identifiers. Information on personal identifiers will include name, date of birth, and a unique national ID number. This information is stored only for the purposes of subject follow-up and will not be shared with NCI.

Serum collected as part of the study will be retained for use in the future. Such use will be restricted to assays required to address questions related to immune response to HPV and HPV vaccination. Note that future of use of serum does not include any genetic analyses. The participant or her parent or legal guardian can refuse bio-banking of biospecimens without affecting her participation.

Plan for return of individual study results to participants

Results from the molecular assays will be used only for research purposes.

Evaluation of anticipated benefits and risks/discomforts

Participants will receive the benefit of HPV vaccination. Additionally, women who are within the age range to receive screening for cervical cancer in the Costa Rica healthcare system, will receive education concerning the importance of screening for secondary prevention.

The risks associated with this research are believed to be minimal. Both vaccines are approved products and known risks include injection site reactions (mainly pain), dizziness, and headache. A series of reviews of safety data have been conducted by both the Institute of Medicine (IOM) and the WHO Global Advisory Committee on Vaccine Safety (GACVS). These reviews continue to affirm that no safety issues that would alter the recommendation for use of the HPV vaccines have been found (25, 67, 68).

We will monitor all study participants for adverse events (AEs) at the clinic for at least 15 minutes after vaccination with appropriate medical treatment readily available in case of a rare anaphylactic reaction.

While no risks have been observed for vaccination during pregnancy (61, 62), if a potential trial participant is planning to become pregnant, or has an equivocal or positive pregnancy test, we will defer her enrollment and vaccination according to the vaccines use and recommendations approved by the Costa Rican Ministry of Health. This is according to the labels for these vaccines, which state that the safety and effectiveness of the vaccines has not been established among pregnant women. Pregnancy test results will also be handle as described above to protect participants (See section "*Safeguards for vulnerable populations*").

HPV vaccination has no effect of behavior or sexual risk taking. There are extensive data from more than 20 studies that occurred in multiple world regions, and across a broad age range including girls and women (69). Outcomes in these studies included measured changes in sexual behaviors such as age at sexual debut, risky sexual behaviors, use of condoms and contraception. More importantly, clinical indicators such as rates of sexually transmitted infections, HIV, and pregnancy have also demonstrated no differences between vaccinated and unvaccinated populations. In the event that one dose does not elicit strong immune response and girls do not obtain adequate protection, data suggest that these vaccinated girls/women would not be at higher risk of HPV acquisition due to increased sexual risk taking.

From venipuncture, risks include a small bruise and the minor pain associated with a needle-stick. Every effort will be made by the qualified phlebotomists to be hired by the Costa Rican collaborators, to minimize the pain from needle-stick and subsequent bruising or bleeding.

Compensation

Study participants will not be compensated for their participation and time, but they will be reimbursed for transportation costs. Participants will be reimbursed their actual costs, which will depend upon where they travel from and whether they came accompanied or unaccompanied by additional persons such as parents or other family members. The actual cost is determined by self-report and staff knowledge of the standard local fares (e.g., local bus fares), and expected taxi fares, for example from bus station to home. Finally, if the participant is present at the clinic during breakfast, lunch, or dinner time, food will be offered; throughout the day, coffee, water, and light snacks also will be available.

ETHICAL CONSIDERATIONS AND TRIAL OVERSIGHT

The trial will make use of several methods for data and safety monitoring, and oversight.

Data and Safety Monitoring

A Data Safety Monitoring Board (DSMB) will meet during the vaccination phase of the trial and as required during the follow-up phase. After each review, members of the DSMB will summarize their findings and provide recommendations to the NCI Principal Investigator, the DCEG Scientific Director, the Costa Rican Principal Investigator, and the NCI Ethics Review Panel (ERP) and Costa Rican IRB. Should the NCI or local IRB have any concerns regarding trial progress or participant safety, these will be addressed directly to the DSMB with a copy to the Costa Rica and NCI Principal Investigators. Full communication between the ERP, IRB, and the DSMB is essential and will be the responsibility of the NCI Principal Investigator. A DSMB charter document will detail the charge to the committee, membership, implementation and reporting plans.

Delivering trial results to participants and actions if study shows one dose failure.

As previously mentioned, the efficacy of a single dose will only be known towards the end of the three-year follow-up. At the conclusion of this study, if single-dose vaccination is immunologically inferior compared to the standard arm in this study, the investigators will inform single-dose recipients, and offer the second dose of the HPV vaccine.

In the event that delivery of vaccine information to participants is necessary, we will seek the permission of the local IRB to make contact with participants who have withdrawn from the study. This authorization is required according to GCP and laws governing research in Costa Rica, which otherwise bar study personnel from making contact with this population. Based on our past experiences with the CVT cohort, we estimate that fewer than 2 percent of participants will be truly lost to follow-up (i.e., we cannot find or contact them), so we are confident we will be able to follow-up with most participants, as needed, provided that we have the IRBs authorization. Nevertheless, as an added precaution, we will include language in the

consent and assent form urging all participants to retain the consent form so that they are able to contact the study in the future even if they discontinue participation. At the point of withdrawal, we will also emphasize these instructions and will provide participants who are withdrawing from the study with whatever information we can at the time they withdraw, if possible (i.e., if they agree to receive the information).

Working Group
In addition to the DSMB, an independent Scientific Working Group (WG) will be established by NCI to advise the study investigators and the Division Director on scientific and policy issues surrounding the trial. The WG membership will consist of individuals with expertise in areas relevant to the conduct of the trial, including clinical trials, epidemiology, biostatistics, virology, vaccinology, immunology, medical ethics, and health advocacy. Members of the WG will be external to NIH to assure independent evaluation. WG members will be vetted for conflicts of interest and will be required to disclose any potential conflict that arises during the study. The WG will be composed of both United States and Costa Rican nationals. The WG will be broadly charged with advising the NCI Principal Investigator and DCEG Scientific Director on scientific and policy issues that arise during the study.

The WG will meet yearly during the vaccination phase of the trial and as required during the follow-up phase. After each review, members of the WG will summarize their findings and provide recommendations and will report them to the NCI Principal Investigator, the DCEG Scientific Director, and the NCI Board of Scientific Councilors (BSC). A WG charter document will detail the charge to the committee, membership, implementation and reporting plans.

Regulatory Body Review
NCI presented the trial concept to the EMA Vaccine Working Party on June 8, 2018 (London, England for preliminary assessment). The concept was favorably received, and investigators were encouraged to proceed and formally seek feedback from the EMA CHMP, along with GSK, the marketing authorization holder of Cervarix. The goal of engaging with EMA is to achieve a one dose label change for Cervarix, if the PRIMAVERA results meet the criteria for non-inferiority.

DATA SHARING PLAN

Sharing research data supports the mission of the National Institutes of Health (NIH) and is essential to facilitate the translation of research results into knowledge, products, and procedures that improve human health. Whenever possible, data collected and/or generated from this study will be made available for secondary research purposes and will be made available no later than at the time of publication. Note that no genetic analyses will be conducted or made available.

Provisions will be taken to protect the subjects' privacy and the confidentiality of the data, including but not limited to, de-identification of subject-level data and limiting secondary research use to what is deemed appropriate in the protocol and informed consent and/or assent forms signed by research participants and their guardians. If there is a question about whether a proposed analysis is authorized within the signed IC, the Working Group, and/or Costa Rican IRB and NCI ERP may be consulted.

When data are shared outside of the NIH for collaborative and non-collaborative research, all data will be anonymized; transfers will be recorded and appropriate approvals will be obtained and documented for human subject and Privacy Act compliance. At minimum written documentation between the NIH and the recipient of the data must be made to memorialize what is being shared and under what conditions. This may be accomplished via Data Transfer Agreements (DTAs) between the NCI and the recipient institution(s). If data are to be shared via a publicly accessible research repository, additional documentation and application requirements may apply.

The study investigators reserve the right to require payment to its contractors to cover their costs to produce data sets and accompanying documentation.

REFERENCES

1. Bouvard V, Baan R, Straif K, Grosse Y, Secretan B, El Ghissassi F, et al. A review of human carcinogens--Part B: biological agents. Lancet Oncol. 2009;10(4):321-2.
2. Cogliano V, Baan R, Straif K, Grosse Y, Secretan B, El Ghissassi F, et al. Carcinogenicity of human papillomaviruses. Lancet Oncol. 2005;6(4):204.
3. Winer RL, Feng QH, Hughes JP, O'Reilly S, Kiviat NB, Koutsky LA. Risk of female human papillomavirus acquisition associated with first male sex partner. Journal of Infectious Diseases. 2008;197(2):279-82.
4. Rodriguez AC, Schiffman M, Herrero R, Wacholder S, Hildesheim A, Castle PE, et al. Rapid clearance of human papillomavirus and implications for clinical focus on persistent infections. Journal of the National Cancer Institute. 2008;100(7):513-7.
5. Rodriguez AC, Schiffman M, Herrero R, Hildesheim A, Bratti C, Sherman ME, et al. Longitudinal Study of Human Papillomavirus Persistence and Cervical Intraepithelial Neoplasia Grade 2/3: Critical Role of Duration of Infection. Journal of the National Cancer Institute. 2010;102(5):315-24.
6. Ferlay J, Shin HR, Bray F, Forman D, Mathers C, Parkin DM. Estimates of worldwide burden of cancer in 2008: GLOBOCAN 2008. International Journal of Cancer. 2010;127(12):2893-917.
7. de Sanjose S, Quint WGV, Alemany L, Geraets DT, Klaustermeier JE, Lloveras B, et al. Human papillomavirus genotype attribution in invasive cervical cancer: a retrospective cross-sectional worldwide study. Lancet Oncology. 2010;11(11):1048-56.
8. Giuliano AR, Palefsky JM, Goldstone S, Moreira ED, Penny ME, Aranda C, et al. Efficacy of Quadrivalent HPV Vaccine against HPV Infection and Disease in Males. New England Journal of Medicine. 2011;364(5):401-11.
9. Munoz N, Kjaer SK, Sigurdsson K, Iversen OE, Hernandez-Avila M, Wheeler CM, et al. Impact of Human Papillomavirus (HPV)-6/11/16/18 Vaccine on All HPV-Associated Genital Diseases in Young Women. Jnci-Journal of the National Cancer Institute. 2010;102(5):325-39.
10. Palefsky JM, Giuliano AR, Goldstone S, Moreira ED, Aranda C, Jessen H, et al. HPV Vaccine against Anal HPV Infection and Anal Intraepithelial Neoplasia. New England Journal of Medicine. 2011;365(17):1576-85.
11. Giannini SL, Hanon E, Moris P, Van Mechelen M, Morel S, Dessy F, et al. Enhanced humoral and memory B cellular immunity using HPV16/18 L1 VLP vaccine formulated with the MPL/aluminium salt combination (AS04) compared to aluminium salt only. Vaccine. 2006;24(33-34):5937-49.
12. Future II Study Group. Quadrivalent vaccine against human papillomavirus to prevent high-grade cervical lesions. N Engl J Med. 2007;356(19):1915-27.
13. Wheeler CM, Castellsague X, Garland SM, Szarewski A, Paavonen J, Naud P, et al. Cross-protective efficacy of HPV-16/18 AS04-adjuvanted vaccine against cervical infection and precancer caused by non-vaccine oncogenic HPV types: 4-year end-of-study analysis of the randomised, double-blind PATRICIA trial. Lancet Oncol. 2012;13(1):100-10.
14. Herrero R, Wacholder S, Rodriguez AC, Solomon D, Gonzalez P, Kreimer AR, et al. Prevention of Persistent Human Papillomavirus Infection by an HPV16/18 Vaccine: A Community-Based Randomized Clinical Trial in Guanacaste, Costa Rica. Cancer Discovery. 2011;1(5):408-19.
15. Food and Drug Administration. Prescribing information [package insert]. Gardasil 9 [human papillomavirus 9-valent vaccine, recombinant]. http://wwwfdagov/downloads/BiologicsBloodVaccines/Vaccines/ApprovedProducts/UCM426457pdf. 2016.

16. European Medicines Agency. Product information. Gardasil 2017 [cited 2018 7/14/2018]. Available from: http://www.ema.europa.eu/docs/en_GB/document_library/EPAR_-_Product_Information/human/000703/WC500021142.pdf.
17. European Medicines Agency. Product information. Cervarix. http://wwwemaeuropaeu/docs/en_GB/document_library/EPAR_-_Product_Information/human/000721/WC500024632pdf. 2017.
18. Centers for Disease Control and Prevention. Grading of Recommendations Assessment, Development and Evaluation (GRADE) of a 2-dose schedule for human papillomavirus (HPV)vaccination. https://wwwcdcgov/vaccines/acip/recs/grade/hpv-2-dosehtml. 2016.
19. World Health Organization. Human papillomavirus vaccines: WHO position paper, May 2017. http://appswhoint/iris/bitstream/handle/10665/255353/WER9219pdf;jsessionid=1019E3C7EADD04FCA8DC7B03F979E47B?sequence=1. 2017.
20. Brotherton JML, Fridman M, May CL, Chappell G, Saville AM, Gertig DM. Early effect of the HPV vaccination programme on cervical abnormalities in Victoria, Australia: an ecological study. Lancet. 2011;377(9783):2085-92.
21. Crowe E, Pandeya N, Brotherton JML, Dobson AJ, Kisely S, Lambert SB, et al. Effectiveness of quadrivalent human papillomavirus vaccine for the prevention of cervical abnormalities: case-control study nested within a population based screening programme in Australia. Bmj-British Medical Journal. 2014;348.
22. Gertig DM, Brotherton JML, Budd AC, Drennan K, Chappell G, Saville AM. Impact of a population-based HPV vaccination program on cervical abnormalities: a data linkage study. Bmc Medicine. 2013;11.
23. Read TRH, Hocking JS, Chen MY, Donovan B, Bradshaw CS, Fairley CK. The near disappearance of genital warts in young women 4 years after commencing a national human papillomavirus (HPV) vaccination programme. Sexually Transmitted Infections. 2011;87(7):544-7.
24. Kavanagh K, Pollock KG, Cuschieri K, Palmer T, Cameron RL, Watt C, et al. Changes in the prevalence of human papillomavirus following a national bivalent human papillomavirus vaccination programme in Scotland: a 7-year cross-sectional study. Lancet Infectious Diseases. 2017;17(12):1293-302.
25. World Health Organization. Meeting of the Global Advisory Committee on Vaccine Safety, 7–8 June 2017. Weekly Epidemiological Record= Relevé épidémiologique hebdomadaire. 2017;92(28):393-404.
26. Markowitz LE, Dunne EF, Saraiya M, Chesson HW, Curtis CR, Gee J, et al. Human papillomavirus vaccination: recommendations of the Advisory Committee on Immunization Practices (ACIP). MMWR Recomm Rep. 2014;63(RR-05):1-30.
27. Bruni L, Diaz M, Barrionuevo-Rosas L, Herrero R, Bray F, Bosch FX, et al. Global estimates of human papillomavirus vaccination coverage by region and income level: a pooled analysis. Lancet Global Health. 2016;4(7):E453-E63.
28. Ferlay J, Soerjomataram I, Ervik M, Dikshit R, Eser S, Mathers C, et al. Cancer Incidence and Mortality Worldwide: IARC CancerBase No. 11 [Internet]. GLOBOCAN. 2012;1.0.
29. Kreimer AR, Struyf F, Del Rosario-Raymundo MR, Hildeshim A, Skinner SR, Wacholder S, et al. Efficacy of fewer than three doses of an HPV-16/18 AS04-adjuvanted vaccine: combined analysis of data from the Costa Rica Vaccine and PATRICIA trials. Lancet Oncology. 2015;16(7):775-86.
30. Safaeian M, Sampson JN, Pan YJ, Porras C, Kemp TJ, Herrero R, et al. Durability of Protection Afforded by Fewer Doses of the HPV16/18 Vaccine: The CVT Trial. Journal of the National Cancer Institute. 2018;110(2).

31. Sankaranarayanan R, Joshi S, Muwonge R, Esmy PO, Basu P, Prabhu P, et al. Can a single dose of human papillomavirus (HPV) vaccine prevent cervical cancer? Early findings from an Indian study. Vaccine. 2018.
32. Sankaranarayanan R, Prabhu PR, Pawlita M, Gheit T, Bhatla N, Muwonge R, et al. Immunogenicity and HPV infection after one, two, and three doses of quadrivalent HPV vaccine in girls in India: a multicentre prospective cohort study. Lancet Oncology. 2016;17(1):67-77.
33. Schiller J, Lowy D. Explanations for the high potency of HPV prophylactic vaccines. Vaccine. 2018.
34. Schiller JT, Lowy DR. Raising expectations for subunit vaccine. J Infect Dis. 2015;211(9):1373-5.
35. Centers for Disease Control and Prevention. Human Papillomavirus Vaccination: Recommendations of the Advisory Committee on Immunization Practices (ACIP). Morbidity and Mortality Weekly Report. 2014;63:1-30.
36. Food and Drug Administration. Prescribing information [package insert]. Gardasil [human papillomavirus quadrivalent vaccine, recombinant]. https://wwwfdagov/downloads/BiologicsBloodVaccines/Vaccines/ApprovedProducts/UCM111263pdf. 2006.
37. Food and Drug Administration. Prescribing information [package insert]. Cervarix [human papillomavirus bivalent vaccine, recombinant]. https://wwwfdagov/downloads/BiologicsBloodVaccines/Vaccines/ApprovedProducts/UCM240436pdf. 2009.
38. European Medical Agency. Product information. Gardasil-9. http://wwwemaeuropaeu/docs/en_GB/document_library/EPAR_-_Product_Information/human/003852/WC500189111pdf. 2017.
39. Meites E, Kempe A, Markowitz LE. Use of a 2-Dose Schedule for Human Papillomavirus Vaccination-Updated Recommendations of the Advisory Committee on Immunization Practices. Am J Transplant. 2017;17(3):834-7.
40. Meites E, Kempe A, Markowitz LE. Use of a 2-Dose Schedule for Human Papillomavirus Vaccination - Updated Recommendations of the Advisory Committee on Immunization Practices. MMWR Morb Mortal Wkly Rep. 2016;65(49):1405-8.
41. Centers for Disease Control and Prevention. FDA licensure of bivalent human papillomavirus vaccine (HPV2, Cervarix) for use in females and updated HPV vaccination recommendations from the Advisory Committee on Immunization Practices (ACIP). MMWR Morb Mortal Wkly Rep. 2010;59(20):626-9.
42. Pedersen C, Petaja T, Strauss G, Rumke HC, Poder A, Richardus JH, et al. Immunization of early adolescent females with human papillomavirus type 16 and 18 L1 virus-like particle vaccine containing AS04 adjuvant. The Journal of adolescent health : official publication of the Society for Adolescent Medicine. 2007;40(6):564-71.
43. Block SL, Nolan T, Sattler C, Barr E, Giacoletti KE, Marchant CD, et al. Comparison of the immunogenicity and reactogenicity of a prophylactic quadrivalent human papillomavirus (types 6, 11, 16, and 18) L1 virus-like particle vaccine in male and female adolescents and young adult women. Pediatrics. 2006;118(5):2135-45.
44. Centers for Disease Control and Prevention. FDA licensure of quadrivalent human papillomavirus vaccine (HPV4, Gardasil) for use in males and guidance from the Advisory Committee on Immunization Practices (ACIP). MMWR Morb Mortal Wkly Rep. 2010;59(20):630-2.
45. Centers for Disease Control and Precention. Grading of Recommendations, Assessment, Development, and Evaluation (GRADE) for HPV vaccine for males. http://wwwcdcgov/vaccines/acip/recs/GRADE/hpv-vac-maleshtml. 2012.

46. Centers for Disease Control and Prevention. Recommendations on the use of quadrivalent human papillomavirus vaccine in males—Advisory Committee on Immunization Practices (ACIP). MMWR. 2011;60:1705-8.
47. Dobson SRM, McNeil S, Dionne M, Dawar M, Ogilvie G, Krajden M, et al. Immunogenicity of 2 Doses of HPV Vaccine in Younger Adolescents vs 3 Doses in Young Women A Randomized Clinical Trial. Jama-Journal of the American Medical Association. 2013;309(17):1793-802.
48. Romanowski B, Schwarz TF, Ferguson LM, Peters K, Dionne M, Schulze K, et al. Immunogenicity and safety of the HPV-16/18 AS04-adjuvanted vaccine administered as a 2-dose schedule compared with the licensed 3-dose schedule: results from a randomized study. Human Vaccines. 2011;7(12):1374-86.
49. Einstein MH, Levin MJ, Chatterjee A, Chakhtoura N, Takacs P, Catteau G, et al. Comparative humoral and cellular immunogenicity and safety of human papillomavirus (HPV)-16/18 AS04-adjuvanted vaccine and HPV-6/11/16/18 vaccine in healthy women aged 18-45 years: follow-up through Month 48 in a Phase III randomized study. Hum Vaccin Immunother. 2014;10(12):3455-65.
50. Safaeian M, Porras C, Pan Y, Kreimer A, Schiller JT, Gonzalez P, et al. Durable antibody responses following one dose of the bivalent human papillomavirus L1 virus-like particle vaccine in the Costa Rica Vaccine Trial. Cancer Prev Res (Phila). 2013;6(11):1242-50.
51. Toft L, Tolstrup M, Muller M, Sehr P, Bonde J, Storgaard M, et al. Comparison of the immunogenicity of Cervarix(R) and Gardasil(R) human papillomavirus vaccines for oncogenic non-vaccine serotypes HPV-31, HPV-33, and HPV-45 in HIV-infected adults. Hum Vaccin Immunother. 2014;10(5):1147-54.
52. Barzon L, Squarzon L, Masiero S, Pacenti M, Marcati G, Mantelli B, et al. Neutralizing and cross-neutralizing antibody titres induced by bivalent and quadrivalent human papillomavirus vaccines in the target population of organized vaccination programmes. Vaccine. 2014;32(41):5357-62.
53. GlaxoSmithKline (GSK). Immunogenicity: 3 consecutive lots of GSK Biologicals' HPV-16/18 vaccine administered intramuscularly at 0,1,6 month schedule in healthy females aged 10–25 years and demonstrate non-inferiority of candidate HPV vaccine manufactured by modified production process 2016 [updated Sep 09, 2016. Available from: https://gsk-clinicalstudyregister.com/search/all/1/?search_terms=580299%2F012.
54. Reisinger KS, Block SL, Lazcano-Ponce E, Samakoses R, Esser MT, Erick J, et al. Safety and persistent immunogenicity of a quadrivalent human papillomavirus types 6, 11, 16, 18 L1 virus-like particle vaccine in preadolescents and adolescents: a randomized controlled trial. Pediatr Infect Dis J. 2007;26(3):201-9.
55. Petersen LK, Restrepo J, Moreira ED, Jr., Iversen OE, Pitisuttithum P, Van Damme P, et al. Impact of baseline covariates on the immunogenicity of the 9-valent HPV vaccine - A combined analysis of five phase III clinical trials. Papillomavirus Res. 2017;3:105-15.
56. Joura EA, Giuliano AR, Iversen OE, Bouchard C, Mao C, Mehlsen J, et al. A 9-valent HPV vaccine against infection and intraepithelial neoplasia in women. N Engl J Med. 2015;372(8):711-23.
57. Cortes B, Schiffman M, Herrero R, Hildesheim A, Jimenez S, Shea K, et al. Establishment and operation of a biorepository for molecular epidemiologic studies in Costa Rica. Cancer Epidemiol Biomarkers Prev. 2010;19(4):916-22.
58. Toh ZQ, Russell FM, Reyburn R, Fong J, Tuivaga E, Ratu T, et al. Sustained Antibody Responses 6 Years Following 1, 2, or 3 Doses of Quadrivalent Human Papillomavirus (HPV) Vaccine in Adolescent Fijian Girls, and Subsequent Responses to a Single Dose of Bivalent HPV Vaccine: A Prospective Cohort Study. Clinical Infectious Diseases. 2017;64(7):852-9.

59. Safaeian M, Porras C, Schiffman M, Rodriguez AC, Wacholder S, Gonzalez P, et al. Epidemiological study of anti-HPV16/18 seropositivity and subsequent risk of HPV16 and -18 infections. Journal of the National Cancer Institute. 2010;102(21):1653-62.
60. WHO Expert Committee on Biological Standardization. Recommendations to assure the quality, safety and efficacy of recombinant human papillomavirus virus-like particle vaccines, Technical Report Series, No. 999, Annex 4. 2016.
61. Wacholder S, Chen BE, Wilcox A, Macones G, Gonzalez P, Befano B, et al. Risk of miscarriage with bivalent vaccine against human papillomavirus (HPV) types 16 and 18: pooled analysis of two randomised controlled trials. BMJ. 2010;340.
62. Herrero R, Quint W, Hildesheim A, Gonzalez P, Struijk L, Katki HA, et al. Reduced Prevalence of Oral Human Papillomavirus (HPV) 4 Years after Bivalent HPV Vaccination in a Randomized Clinical Trial in Costa Rica. Plos One. 2013;8(7).
63. Garland SM, Cheung TH, McNeill S, Petersen LK, Romaguera J, Vazquez-Narvaez J, et al. Safety and immunogenicity of a 9-valent HPV vaccine in females 12-26 years of age who previously received the quadrivalent HPV vaccine. Vaccine. 2015;33(48):6855-64.
64. Gilca V, Sauvageau C, Panicker G, De Serres G, Ouakki M, Unger ER. Immunogenicity and safety of a mixed vaccination schedule with one dose of nonavalent and one dose of bivalent HPV vaccine versus two doses of nonavalent vaccine - A randomized clinical trial. Vaccine. 2018;36(46):7017-24.
65. Gilca V, Sauvageau C, Boulianne N, De Serres G, Crajden M, Ouakki M, et al. The effect of a booster dose of quadrivalent or bivalent HPV vaccine when administered to girls previously vaccinated with two doses of quadrivalent HPV vaccine. Hum Vaccin Immunother. 2015;11(3):732-8.
66. Petrosky E, Bocchini JA, Jr., Hariri S, Chesson H, Curtis CR, Saraiya M, et al. Use of 9-valent human papillomavirus (HPV) vaccine: updated HPV vaccination recommendations of the advisory committee on immunization practices. MMWR Morb Mortal Wkly Rep. 2015;64(11):300-4.
67. Committee to Review Adverse Effects of Vaccines, Institute of Medicine. Adverse effects of vaccines: evidence and causality. Stratton K, Ford A, Rusch E, Wright Clayton E, editors: National Academies Press; 2011.
68. World Health Organization. WHO Strategic Advisory Group of Experts (SAGE) on immunization 2015 [updated March 2018. 631-2]. Available from: http://www.who.int/immunization/policy/sage/en/.
69. Madhivanan P, Pierre-Victor D, Mukherjee S, Bhoite P, Powell B, Jean-Baptiste N, et al. Human Papillomavirus Vaccination and Sexual Disinhibition in Females: A Systematic Review. Am J Prev Med. 2016;51(3):373-83.

NCI IRB/ERP AMENDMENTS AND APPROVAL DATES

Approved	Description of Amendment
13 Feb 2019	Versions 1.1 and 1.2 (submitted to the NCI IRB as one amendment): Clarifications and revisions made to the protocol in response to review comments and changes requested by the Costa Rican IRB, as well as some issues identified by the investigators. Some of changes involved corresponding modifications to the assent and consent forms, as well as new materials, namely the consent video scripts.
27 March 2019	Version 1.3: Revisions to the methods, analysis plan, and procedures related to a new follow-up visit one month post-vaccination to collect blood from the Cervarix group (girls ages 9-14). This change was recommended by the CVT Working Group as a means to bridge the 9-10 year olds to the 11-14 year olds in the event that initiation of HPV vaccination in fourth graders, as part of the National Immunization Program, requires exclusion of the 9-10 year olds from the primary analysis. Corresponding modifications also were made to the forms and videos for assent and parental consent, and the information form. The new re-assent form for participants who turn 12 during follow-up was also included, as was the new invitation PowerPoint for recruitment.
22 May 2019	Version 1.4: Revisions to the objectives, overview, and analysis plan requested by the EMA, a clarification to the content regarding the field response to the national vaccine program, and a change in the exclusion criteria to remove latex, and an increase in the accrual ceiling to permit the enrollment of up to 100 additional participants per study group.
7 July 2022	Version 1.5: Revisions to the analysis plan to incorporate evolving knowledge about the impact of the national vaccine program, and changes to reflect that NCI ethical oversight will be conducted by an ethics review panel, no longer the NCI IRB.
3 March 2023	Version 1.6: Revisions to the analysis plan to add HPV6/11 testing of selected participants and move SEAP testing to 36-month visit.

Statistical Analysis Plan to Address the Primary and Secondary Objectives for the Trial Entitled

"Non-inferiority trial comparing immunogenicity from 1-dose of bivalent HPV vaccine in girls to 3-doses of quadrivalent vaccine in women: The PRIMAVERA-ESCUDDO Trial ('Puente de Respuesta Inmunológica para Mejorar el Acceso a Vacunas y ERrAdicar el cancer')"

(NCI Protocol 19-C-009**)**

Version 1.0

Date: June 7, 2022

Table of Contents

1. Introduction 80

2. Objectives 80

2a. Primary Objective 80

2b. Secondary Objectives 80

3. Analytical Cohorts 81

3a. Primary Analytical Cohort 81

3b. Secondary/Exploratory Analytical Cohorts 81

4. Endpoints 82

5. Implementation of Analysis 82

6. Controlling the Type-I error rate 82

7. Statistical Analyses for Immunogenicity 82

7a. Methods for The Primary Objective 82

7b. Methods for The Secondary Objectives 83

7c. Methods for Sensitivity and Exploratory Analyses 83

8. Statistical Analyses for Evaluating ELISA assays 84

1. Introduction

This document describes the pre-planned analysis for the trial entitled "**Non-inferiority trial comparing immunogenicity from 1-dose of bivalent HPV vaccine in girls to 3-doses of quadrivalent vaccine in women**".

2. Objectives

2a. Primary Objective

Primary Objective (PO): To demonstrate that the immunogenicity (as determined by ELISA) of a single dose of Cervarix in 9-14 year old girls is non-inferior to the immunogenicity of three doses of Gardasil, administered at 0, 2, and 6 months, in 18-25 year old women 36 months after initial vaccination, with an interim analysis at 24 months after initial vaccination.

Either of following criteria will qualify as non-inferiority:

- The interim analysis will assess non-inferiority at 24 months. Non-inferiority will be demonstrated if the lower limits for the two-sided 99% confidence intervals of the GMT ratios (1-Dose Cervarix divided by 3-Doses of Gardasil at 24 months) for both HPV-16 and HPV-18 antibody levels exceed 0.67.
- The primary analysis will assess non-inferiority at 36 months. Non-inferiority will be demonstrated if the lower limits for the two-sided 96% confidence intervals of the GMT ratios (1-Dose Cervarix divided by 3-Doses of Gardasil at 36 months) for both HPV-16 and HPV-18 antibody levels exceed 0.67.

2b. Secondary Objectives

Secondary Objective 1 (SO1): To compare the distribution of both HPV-16 and HPV-18 antibodies levels, assessed at 24 and 36 months after initial vaccination, following a single dose of Cervarix in 9-14 year old girls and following three doses of Gardasil, administered at 0, 2, and 6 months, in 18-25 year old women.

Secondary Objective 2 (SO2): To compare rates of seroconversion based on both HPV-16 and HPV-18 antibody levels, assessed at 24 and 36 months after initial vaccination, following a single dose of Cervarix in 9-14 year old girls and following three doses of Gardasil, administered at 0, 2, and 6 months, in 18-25 year old women.

Secondary Objective 3 (SO3): To compare GMTs, distributions, and seroconversion rates for HPV-16 and HPV-18 antibodies, assessed at 24 and 36 months after initial vaccination, following a single dose of Cervarix in 11-14 year old girls and following three doses of Gardasil, administered at 0, 2, and 6 months, in 18-25 year old women.

Secondary Objective 4 (SO4): To compare GMTs, distributions, and seroconversion rates for HPV-16 and HPV-18 antibodies, assessed at 1-month and 1-year after vaccination, following a single dose of Cervarix in 9-10 year old girls and following a single dose of Cervarix in 11-14 year old girls.

Secondary Objective 5 (SO5): To evaluate whether baseline variables (i.e. age, enrollment date, district) are associated with GMTs, distributions, and seroconversion rates for HPV-16 and HPV-18 antibodies, assessed at 24 and 36 months after initial vaccination.

We note that SO3 and SO4 are, in part, included in this study because of Costa Rica's national vaccination program that aimed to vaccinate 10-year-old girls starting in 2019.

3. Analytical Cohorts

3a. Primary Analytical Cohort

The According to Protocol (ATP) cohort for a specified HPV type (i.e. HPV 16 or HPV 18) and timing (i.e. 24-month or 36-month) are the participants who satisfy the following set of requirements:

● Received the correct number of doses within the predefined vaccination windows. Denoting T_1 as the time of the first vaccination, the window for the second vaccination is between T_1+30 days and T_1+90 days. Denoting T_2 as the time of the second vaccination, the window for the third vaccination is between T_2+90 days and T_1+360 days.

● Not seropositive at baseline for the specified HPV type.

● Blood collected within the predefined follow-up window. Denoting T_1 as the time of the first vaccination, the window for the 24-month visit is between T_1+700 days and T_1+912 days and the window for the 36-month window is T_1+1065 days and T_1+1278 days.

● No HPV vaccination outside of the study prior to blood collection. The participant did not report receiving an HPV vaccine and, among those tested, did not have HPV6 and HPV11 vaccine-level antibodies.

Note, the ATP cohort for a given analysis depends on both the timing of the analysis and the specified HPV type.

3b. Secondary/Exploratory Analytical Cohorts

We will also consider the: (i) modified Intention-to-Treat (ITT) cohort, including all participants receiving the correct number of doses and with blood collected during the predefined follow-up window; (ii) Seropositive-ATP (SP-ATP) cohort that includes participants who are seropositive at baseline, but otherwise satisfy the ATP requirements; and (iii) Serocombined-ATP (SC-ATP)

cohort that combines the ATP and SP-ATP cohorts. Furthermore, for SO4, we will consider all girls who are not seropositive at baseline, had blood collected between T_1 + 14 and T_1 + 90 days for the 1 month analysis and between T_1 + 274 and T_1 + 547 days for the 1 year analysis, and did not receive an HPV vaccination outside of the study prior to their blood collection. Additionally, we will test for HPV6/HPV11 antibodies in a subset of participants who are at-risk for external HPV vaccination or show serologic evidence of additional HPV vaccine doses.

4. Endpoints

The four endpoints are HPV-16 and HPV-18 specific antibody results obtained from blood specimens (serum) collected at the 24- (interim) and 36-(primary) month visit (i.e. 4 endpoints = 2 HPV types x 2 time-points). All antibody levels are measured by HPV type-specific ELISA. Two additional endpoints, HPV-16 and HPV-18 antibody levels measured from serum collected at the 1-month visit and the 1-year visit, will also be included to achieve Secondary Objective 4 (SO4).

5. Implementation of Analysis

The final 24- and 36-months analyses will be performed once the corresponding follow-up window for the last enrolled participant closes

6. Controlling the Type-I error rate

The analysis defined in the following section ensures that under the null hypothesis of inferiority, the probability of declaring non-inferiority (i.e. type I error rate) is less than 0.025 for HPV-16 and HPV-18 endpoints, respectively.

7. Statistical Analyses for Immunogenicity

7a. Methods for The Primary Objective

We will estimate the GMT ratios for HPV-16 and HPV-18 at 24 and 36 months in the corresponding ATP cohort.

We define $R^H(t) = GMT_1^H(t)/GMT_3^H(t)$ to be the GMT ratio for HPV-H (i.e. HPV-16 or HPV-18) at time point t (i.e. 24 or 36 months) when comparing antibody levels in 9-14 year old girls receiving 1 dose of Cervarix to levels in 18-25 year old women receiving 3 doses of Gardasil.

We will estimate $R^H(t)$ by fitting a generalized estimating equation (GEE) model where the dependent variable is the log-antibody level and the independent variable is study-group. Denoting the estimated coefficient for study-group by $\hat{\beta}^H(t)$ and the corresponding robust standard error by $\hat{\sigma}^H(t)$, we define our estimate by $\hat{R}^H(t)$ = exp $(\hat{\beta}^H(t))$ and define the two-sided (1-α)% confidence interval (CI), $(\hat{R}^H_{\alpha/2}(t), \hat{R}^H_{1-\alpha/2}(t))$, by exp $(\hat{\beta}^H(t) \pm z_{1-\alpha/2}\hat{\sigma}^H(t))$, where $z_{1-\alpha/2}$ is the 1-α/2 quantile of a normal distribution. In the model, we will group girls into clusters of full siblings and use an exchangeable correlation matrix.

We will declare non-inferiority if $\min\left(\hat{R}^{16}_{0.005}(24), \hat{R}^{18}_{0.005}(24)\right) > 0.67$ or, upon failure of that criterion, $\min\left(\hat{R}^{16}_{0.02}(36), \hat{R}^{18}_{0.02}(36)\right) > 0.67$. Note, under the null hypothesis that both $\min\left(R^{16}(24), R^{18}(24)\right) < 0.67$ and $\min\left(R^{16}(36), R^{18}(36)\right) < 0.67$, the probability of declaring non-inferiority or the Type I error rate is less than 0.025.

7b. Methods for The Secondary Objectives

Secondary Objective 1 (SO1): For each combination of HPV-type, time-point, and study group, we will describe the distribution of antibody levels by showing the reverse cumulative distributions (i.e. 1-cumulative distribution function) in the ATP cohort.

Secondary Objective 2 (SO2): For each combination of HPV-type, time-point, and study group, we will report the proportion of the ATP cohort who seroconvert (i.e. achieve HPV-16 or HPV-18 antibody levels greater than 1.41 international units (IU)/mL or 1.05 IU/mL, respectively) and exact 95% confidence intervals. For each HPV-type and time-point, we will assess whether seroconversion rates differ between study groups using fisher's exact test.

Secondary Objective 3 (SO3): We will repeat the analyses described for the primary objective and secondary objectives 1 and 2 restricted to 11-14-year-old girls. We will also perform a direct comparison of the antibody levels in 9-10 and 11-14 year old girls (e.g. compare GMT in GEE w/ age group as the independent variable, compare seroconversion rates by fisher's exact test).

Secondary Objective 4 (SO4): We will repeat the analyses described for the primary objective and secondary objectives 1 and 2 to compare antibody levels at 1-month and 1-year post-vaccination in girls 9-10 years old girls and in girls 11-14 years old.

Secondary Objective 5 (SO5): We will consider the following baseline covariates: age, enrollment date, and district. Within each study-group and at each time point, we will report the GMTs and their 95% CIs for HPV-16 and HPV-18 antibody levels in subgroups defined by these baseline covariates. Moreover, we will use GEEs to assesses the association between GMTs and these baseline covariates and show both reverse cumulative distributions and seroconversion rates by subgroup.

7c. Methods for Sensitivity and Exploratory Analyses

We will repeat the methods for the primary and secondary objectives using the ITT, SP-ATP, and SC-ATP cohorts instead of the ATP cohort.

We will repeat the methods for the primary and secondary objectives after removing potential outliers (e.g. participants potentially exposed to a vaccination outside of study) where the antibody level is 2xIQR above the 75th percentile.

We will assess whether antibody levels are changing over time. For each combination of study group and HPV type, we will compare GMTs at 24 and 36 months.

8. Statistical Analyses for Evaluating ELISA assays And Comparability with SEAP-NA

HPV binding is measured by ELISA. The quantity of HPV16 and 18 IgG antibody response measured by VLP ELISA will be utilized to address immunogenicity endpoints. To further define the primary analytic cohort, HPV6 and HPV11 antibodies will be measured by VLP ELISA in a subset of participants to confirm, beyond self-report, that the according to protocol analytic cohort includes only girls who did not receive external HPV vaccination through the national program, which uses quadrivalent vaccine (HPV 6, 11, 16, 18). This will assure that girls in the single-dose HPV vaccination arm did not get additional doses.

HPV neutralization is measured by SEAP-NA. Antibodies levels measured by ELISA will be compared to neutralizing antibody levels measured by the pseudovirion-based neutralization assay, SEAP-NA. This nested study will select 24- month serum samples from a random set of 100 baseline seronegative 18-25 year old women and a random set of 100 baseline seronegative 9-14 year old girls, and then evaluate the HPV-16 and HPV-18 antibody levels in each of those 200 samples using both ELISA and SEAP-NA. For each HPV type, we will perform a Deming regression comparing log(ELISA) levels with log(SEAP) levels, where log(ELISA) is the dependent variable and log(SEAP) is the independent variable. We will confirm that ELISA is an acceptable assay for the primary endpoint if the 95% confidence intervals of the regression slope excludes $1-\Delta$ and $1+\Delta$ for both HPV types, with $\Delta = 0.3$. If that criterion is not achieved, then we will initiate a discussion with relevant parties to assess acceptability of the assay.
